# Masked Phenolic-Selenium Conjugates: Potent and Selective Antiproliferative Agents Overcoming P-gp Resistance

**DOI:** 10.3390/ph13110358

**Published:** 2020-10-31

**Authors:** Paloma Begines, Lucía Sevilla-Horrillo, Adrián Puerta, Rebecca Puckett, Samuel Bayort, Irene Lagunes, Inés Maya, José M. Padrón, Óscar López, José G. Fernández-Bolaños

**Affiliations:** 1 Departamento de Química Orgánica, Facultad de Química, Universidad de Sevilla, Apartado 1203, E-41071 Seville, Spain; pbegines@us.es (P.B.); samuel_etegarrobo@hotmail.com (S.B.); imaya@us.es (I.M.); 2 Escuela Politécnica Superior, Universidad de Sevilla, Virgen de África 7, E-41011 Seville, Spain; lucia_sh_93@msn.com (L.S.-H.); rebpucper@hotmail.com (R.P.); 3 BioLab, Instituto Universitario de Bio-Orgánica “Antonio González” (IUBO-AG), Universidad de La Laguna, c/ Astrofísico Francisco Sánchez 2, E-38206 La Laguna, Spain; apuertaa@ull.es (A.P.); roslagunes@uv.mx (I.L.)

**Keywords:** phenolics, organoselenium, antioxidant, antiproliferative, P-gp

## Abstract

Cancer accounts for one of the most complex diseases nowadays due to its multifactorial nature. Despite the vast number of cytotoxic agents developed so far, good therapeutic approaches are not always reached. In recent years, multitarget drugs are gaining great attention against multifactorial diseases in contraposition to polypharmacy. Herein we have accomplished the conjugation of phenolic derivatives with an ample number of organochalcogen motifs with the aim of developing novel antiproliferative agents. Their antioxidant, and antiproliferative properties (against six tumour and one non-tumour cell lines) were analysed. Moreover, in order to predict P-gp-mediated chemoresistance, the P-glycoprotein assay was also conducted in order to determine whether compounds prepared herein could behave as substrates of that glycoprotein. Selenium derivatives were found to be significantly stronger antiproliferative agents than their sulfur isosters. Moreover, the length and the nature of the tether, together with the nature of the organoselenium scaffold were also found to be crucial features in the observed bioactivities. The lead compound, bearing a methylenedioxyphenyl moiety, and a diselenide functionality, showed a good activity (GI_50_ = 0.88‒2.0 µM) and selectivity towards tumour cell lines (selectivity index: 14‒32); moreover, compounds considered herein were not substrates for the P-gp efflux pump, thus avoiding the development of chemoresistance coming from such mechanism, commonly found for widely-used chemotherapeutic agents.

## 1. Introduction

Organoselenium chemistry has provided modern organic synthesis with valuable intermediates and catalysts [1], but it has also emerged as a powerful tool in Medicinal Chemistry [2]. A plethora of selenium-containing derivatives have been designed and tested, eliciting quite diverse bioactivities, ranging from antimicrobial [3], anti-inflammatory [4], antidiabetic [5], anti-Alzheimer [6] to particularly anticancer [7,8,9]; concerning the latter activity, selenium derivatives not only exhibit relevant antiproliferative features, but have also been used in adjuvant therapies in mice [10], or as photosensitizers in photodynamic therapy [11], and are therefore considered as candidates for the development of new anticancer therapies. Interestingly, inorganic selenium sources usually provoke higher genotoxic stress, leading to more hazardous effects than their organic counterparts [12].

Organoselenium motifs have been combined with many other pharmacophores, like phenolic compounds [13,14], iminosugars [15], steroids [16,17], non-steroidal anti-inflammatory drugs [18], heterocycles [19,20,21], nucleosides [22], quinones [23], or metal coordination complexes [24] among others, or incorporated into the structures of natural products, like carbohydrates, giving access to the so called selenosugars [25]. The combination of chitosan and selenium nanoparticles has recently been reported as a vector for the transportation of mRNA for amplifying immune response in the potential development of cancer vaccines [26].

The mechanism involved in the anticancer properties of organoselenium derivatives still remains unclear. Some of them have proved to inhibit key enzymes which are abnormally overexpressed in tumour cells, like carbonic anhydrases (IX and XII isoforms) [27] or histone deacetylases [28]. However, what makes selenium unique, compared to its chalcogen analogue sulfur is, besides differences in bond lengths and energies, its strong redox character [29]. In fact, organoselenium compounds might behave as antioxidant agents, thus with a chemopreventive utility, or as pro-oxidant agents, provoking cell death probably through an apoptotic pathway upon exacerbation of oxidative stress [30,31]. In that sense, selenium-containing drugs can act as redox modulators, many of them showing preference to malignant cells [12]; pro-oxidant activity can be attributed to reactive oxygen species (ROS) induction [32], oxidation of thiol functionalities in proteins, and modification of chromatin. As a result, a series of biological downstream effects take place, affecting the activity of kinases, caspases, DNA repair machinery, or transcription factors [12].

**Figure 1:**
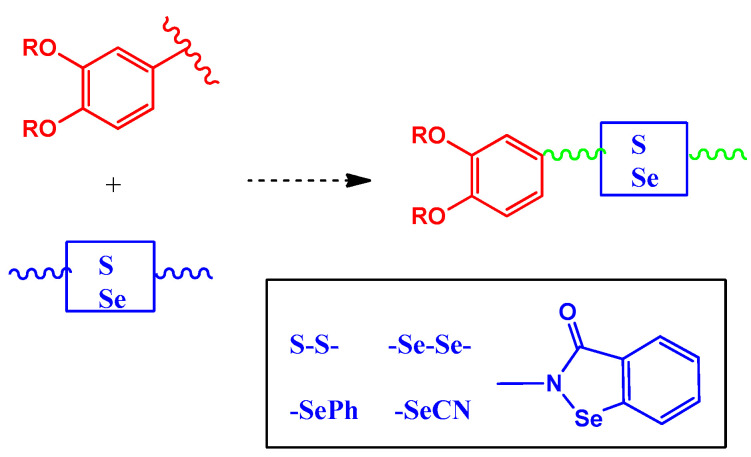
General structure of the chalcogen-polyphenol conjugates prepared herein.

The main purpose pursued herein has been the access to new chalcogen-based multifunctional drugs (Figure 1) with potential utility against cancer as redox chemotherapeutics. We have combined a series of organosulfur and organoselenium motifs (e.g., disulfide, diselenide, selenide, selenocyanato, benzoselenazolone) with an *o*-dihydroxyphenyl residue (catechol moiety), a functionality commonly found in phenolic structures produced as secondary metabolites by plants [33] and endowed with relevant biological properties, like anti-inflammatory [34], neuroprotective [35], or antiproliferative [36,37,38], mainly due to their strong antioxidant properties [33]. The combination of the organoselenium and phenolic motifs, both showing either antioxidant (ROS-user) or pro-oxidant (ROS-enhancer) properties will provide compounds with two redox centres. Both pharmacophores can complement each other, and have a synergic effect in the modulation of the bioactivities of the compounds, in particular, their anticancer properties. For instance, these two redox centres might be involved in the generation of high levels of ROS in malignant cells, thus leading to cell death through an apoptotic pathway. This pattern, with two redox centres is a motif that has been reported for organoselenium/quinones [23] or ferrocene/phenols [39] as an interesting approach for the development of new chemotherapeutic agents targeting the redox dysregulation in cancer; compounds with even three and four redox centres combining chalcogen atoms and quinones have also been reported [40] to be strong antiproliferative agents. Modification of the nature of the chalcogen atom, the nature and the length of the tether and substituents on the aryl moiety can provide valuable structure-activity relationships. Besides targeting redox status, incorporation of certain selenium functionalities (e.g., selenocyanato, diselenide, Figure 1) might diversity the potential targets when tackling cancer, as such scaffolds could interfere overexpressed enzymes in tumour cells, like histone deacetylases. Moreover, we also envisioned the possibility of blocking the catechol functionality with a methylenedioxy function, a structural motif that might afford a better bioavailability when crossing cell membranes, and might also allow the inhibition of cytochrome P450 [41], a therapeutic target against cancer.

## 2. Results

### 2.1. Chemistry

We envisioned the possibility of linking dichalcogenide and catechol moieties through an ester tether; for that purpose, we attempted coupling naturally-occurring hydroxytyrosol **1**, the most remarkable phenolic derivative in olive tree [33] with 4,4’-dithiobisbutyric acid.

Several conditions were assayed, like conversion of the dicarboxylic acid into the corresponding acid dichloride with refluxing neat SOCl_2_ followed by coupling with **1** in the presence of tetrabutylammonium hydrogensulfate [42] at rt and using THF as solvent. We also attempted coupling of the transient acid chloride coming from the dicarboxylic acid, generated again with SOCl_2_, with **1** at rt and THF as solvent, in the presence of Ce(III) (0.5 equiv.); this catalyst has been previously used for the chemoselective esterification of hydroxytyrosol **1** on the aliphatic hydroxyl group with alkyl halides with different chain lengths [43]. A biocatalyzed process with different ratio of *Candida antarctica B* [44] involving **1** and the dicarboxylic acid **2** was also tested (THF, 50 °C). Unfortunately, all these procedures proved to be unsuccessful, as no conversion was observed by TLC.

**Scheme 1:**
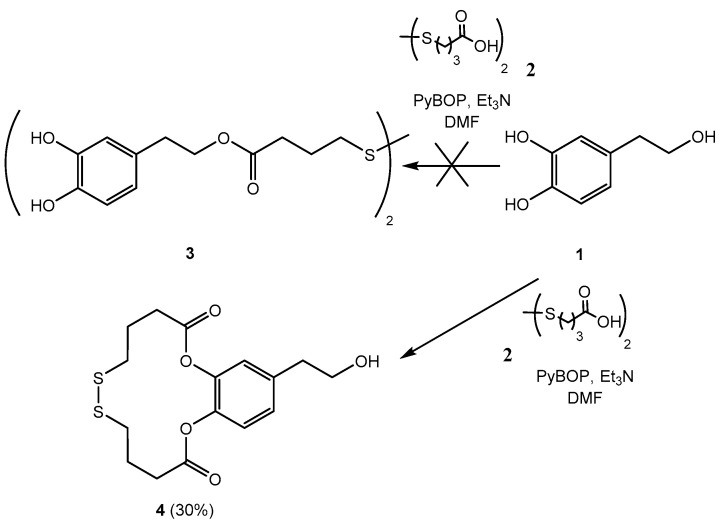
Attempted esterification of hydroxytyrosol **1.**

Unexpectedly, treatment of **1** with the same disulfide in the presence of PyBOP, a well-known peptide coupling reagent, did not furnish **3**, but the 14-membered ring bicyclic derivative **4** in moderate yield (Scheme 1); presumably, the mild basic conditions provided by Et_3_N enhanced nucleophilicity of the phenolic hydroxyl groups compared to the aliphatic counterpart. ^1^H-NMR unambiguously proved that phenolic hydroxyl groups had been esterified, as protons H-2, H-5 and H-6 resonated above 7 ppm, as previously observed for acetylated olive polyphenols [45].

**Scheme 2:**
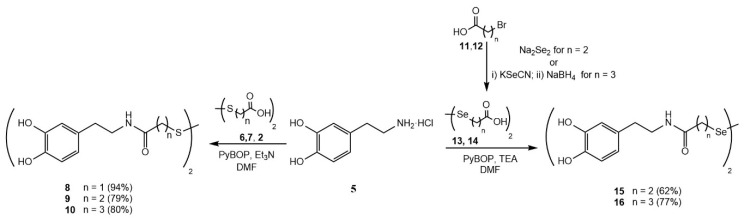
Synthesis of symmetrical phenolic disulfides and diselenides.

Due to the difficulties encountered for linking dichalcogens with an ester-based scaffold, we decided to shift to an amide-type linker, and no other coupling reagents or different reactions were attempted. For that purpose, naturally-occurring dopamine **5** seemed to be a good building block. Combination of dopamine with commercially-available disulfides **6**, **7** and **2** (n = 1, 2 and 3, respectively) in the presence of PyBOP as the peptide coupling reagent, furnished the corresponding symmetrical disulfides **8**‒**10** in excellent yields (79–94%, Scheme 2); DMF was used as solvent, and Et_3_N as base.

In order to analyse the influence of the chalcogen atom on the biological properties, the corresponding seleno-isosters **15**, **16** were accessed (Scheme 2); they key diselenides **13** and **14** (n = 2, 3, respectively, Scheme 2) were obtained from the corresponding ω-bromo carboxylic acids by nucleophilic displacement with freshly-prepared Na_2_Se_2_ (reduction of elemental selenium black with NaBH_4_, for **13**), or with KSeCN, followed by reduction with NaBH_4_ (for **14**, Scheme 2). PyBOP-mediated peptide coupling with dopamine **5** afforded diselenides **15**, **16**, which were isolated in good yields (62% and 77%, respectively, Scheme 2).

**Scheme 3:**
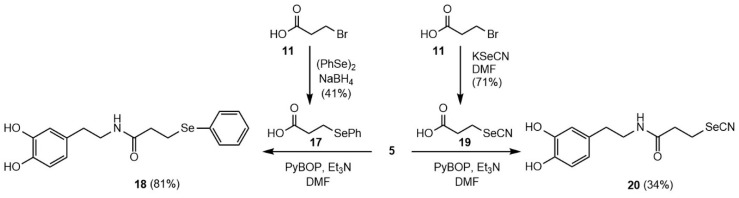
Synthesis of phenolic aryl selenide **18** and selenocyanate **20.**

In order to diversify the functionality of the phenolic derivatives, phenyl selenide **18** and selenocyanate **20** were also prepared (Scheme 3); in both cases, 3-bromopropanoic acid **11** was the key synthetic intermediate. For accessing **18**, compound **11** was subjected to a nucleophilic displacement with in situ generated sodium phenyl selenide (obtained by reduction of diphenyl diselenide with NaBH_4_), followed by peptide coupling with dopamine **5** (Scheme 3).

Similarly, nucleophilic displacement of **11** with KSeCN, followed by coupling with **5**, furnished **20** in a moderate yield (34%) (Scheme 3).

**Scheme 4:**
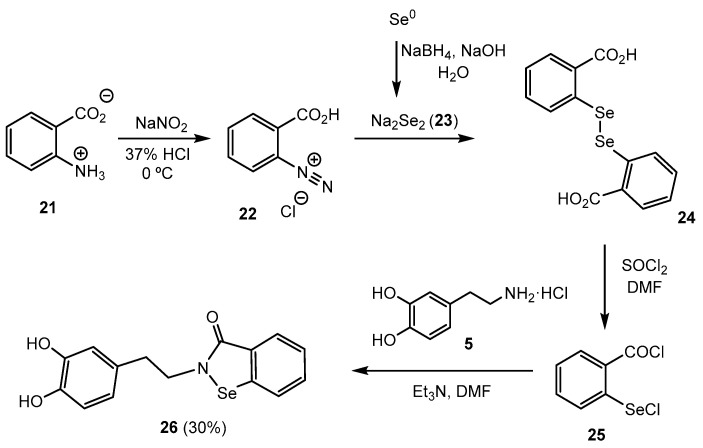
Synthesis of catechol-ebselen hybrid **26**.

We have also carried out the synthesis of the hitherto unknown catechol-ebselen hybrid **26** (Scheme 4). Ebselen (2-phenyl-1,2-benzoselenazol-3-one) is a relevant selenoheterocycle which behaves as an excellent glutathione peroxidase (GPx) mimic [46], a selenoenzyme that exerts a natural defence against oxidative stress. Ebselen has also been proved to ameliorate neural degeneration after cerebral artery occlusion [47], and recently, it has also been found to act as a mimetic of lithium [48], what might be useful against bipolar disorder. 

Treatment of anthranilic acid with sodium nitrite and HCl afforded diazonium salt **22**, which was in turn coupled with in situ generated sodium diselenide to afford diselenide **24** (Scheme 4); reaction with thionyl chloride and DMF as a catalyst furnished transient aryl selenyl choride **25** [49]. Final coupling with dopamine hydrochloride **5** under basic conditions (Et_3_N) furnished ebselen derivative **26** in a moderate yield (30%) (Scheme 4).

**Scheme 5:**
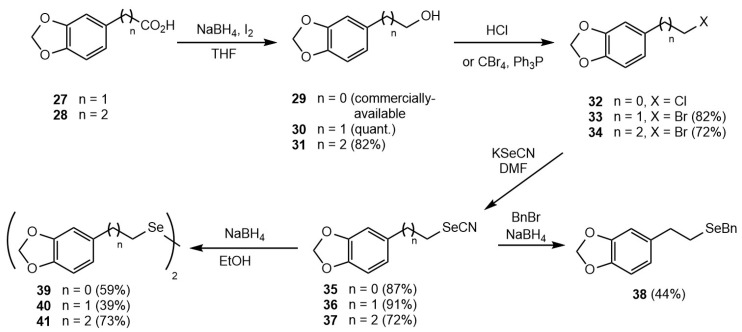
Preparation of *O*-protected phenolic-based selenides, selenocyanates and diselenides.

**Scheme 6:**
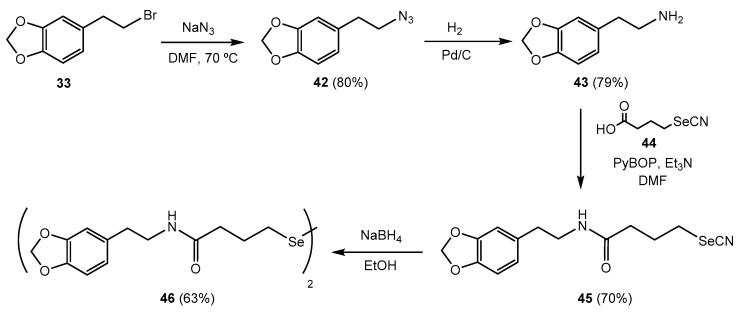
Preparation of *O*-protected selenocyanato **45** and diselenide **46**.

Next structural modification involved the use of *O*-protected phenolic derivatives, and the removal of the amide-type linker (Scheme 5 and Scheme 6). For that purpose, we chose the methylenedioxyphenyl moiety, which is present in natural antioxidants known as sesame lignans (the major components of sesame oil), and also in species, like black pepper [50]. Numerous compounds containing such acetal functionality act as inhibitors of cytochrome P450, a family of heme-containing enzymes that catalyse the oxidative metabolization of hydrophobic substrates, either endogenous or exogenous, into hydrophilic derivatives [50]. Cytochrome P450 has been considered as a target in cancer therapies [41] so incorporation of a methylenedioxyphenyl scaffold in the compounds prepared herein might improve the profile of the selenium-containing derivatives by interacting simultaneously with another key target of tumour cells.

Thus, reduction of *O*-protected carboxylic acids **27**, **28** achieved by the combination of NaBH4 and I_2_ [51] afforded the corresponding alcohols (Scheme 5); Appel-type reaction (Ph_3_P + tetrahaloalkanes) on **29**‒**31** was attempted for inserting a bromine atom on the ω-position of the linker (Scheme 5). Although such reaction proceeded with high yields for the preparation of **33**, **34** (n = 1, 2, 82% and 72%, respectively), when applied to piperonyl alcohol **29** (n = 0), a spontaneous decomposition of the brominated derivative took place, as revealed by TLC; accordingly, in that case, bromine atom was replaced by chlorine as an attempt to reduce the intrinsic reactivity of the benzyl bromide derivative (Scheme 5); crude **32** was used without any further purification.

Nucleophilic substitution accomplished with KSeCN in DMF (Scheme 5) furnished the expected ω-selenocyanato derivatives **35**‒**37** in good to excellent yields (72‒91%); such compounds were the key synthetic intermediates for the preparation of *Se*-Bn derivative **38** upon NaBH4-promoted reduction of the selenocyanato moiety (Scheme 5) and in situ alkylation of the transient sodium selenide with benzyl bromide, and also for the preparation of symmetrical diselenides **39**‒**41** upon reduction with NaBH_4_ in the presence of oxygen (Scheme 5).

Finally, a combination of the amide functionality contained in diselenides **15**/**16** and the *O*-methylidene acetal scaffold of derivatives **39**‒**41** was envisioned (Scheme 6). Thus, nucleophilic displacement of NaN_3_ on brominated derivative **33**, followed by reduction of the azido moiety, and PyBOP-promoted peptide coupling with 4-selenocyanato butyric acid **44** furnished **45** in a 44% overall yield (three steps, Scheme 6). Subsequent reduction gave symmetrical diselenide **46** in a 63% yield, which incorporated a protected catechol moiety, an amide-based tether and a diselenide functionality as the key structural motifs.

### 2.2. Biological Assays

#### 2.2.1. Antioxidant Properties

In vitro antioxidant properties of key unprotected phenolic derivatives were analyzed using two different methodologies: free radical scavenging capacity, using the DPPH method (2,2-diphenyl-1-picrylhydrazyl), a HAT (hydrogen atom transfer) procedure, widely-used for both natural and synthetic derivatives, and also the capacity for scavenging H_2_O_2_, one of the most common ROS in degenerative diseases, including cancer.

##### Free Radical Activity (DPPH Method)

DPPH is a commercially-available stable free radical commonly used for in vitro testing of radical scavenging properties of antioxidants [52]. Its methanolic solution exhibits a deep purple color (λ_max_ = 515 nm); the strong absorption is consequently decreased upon reaction with an antiradical agent, being followed spectrophotometrically. Table 1 depicts the EC_50_ values for the compounds prepared herein, including hydroxytyrosol (HT **1**, an olive tree antioxidant [33]) and dopamine (used herein as the building block for the synthesis of the majority of the compounds) as reference compounds. 

##### H_2_O_2_ Scavenging Properties

Herein, following Bahorun’s methodology [53], in vitro levels of H_2_O_2_ were measured in an indirect fashion, by registering the H_2_O_2_-mediated oxidation of phenol red in the presence of horseradish peroxidase, to give a chromophore with a maximum absorption at 610 nm. Accordingly, in the presence of an antioxidant agent, there is an inverse correlation between the observed absorbance, and the scavenging properties. Table 2 depicts the H_2_O_2_-scavenging properties of compounds prepared herein, and tested at a final 50 µM concentration.

#### 2.2.2. Antiproliferative Activity

Organochalcogen derivatives prepared herein were evaluated in vitro as potential antiproliferative agents, using a panel of six human tumour cell lines: A549 (non-small cell lung), HBL-100 (breast), HeLa (cervix), SW1573 (non-small cell lung), as drug sensitive lines, T-47D (breast) and WiDr (colon) as drug resistant lines. In addition to precursors **2**, **6**, **7**, **13**, **14**, **17**, **19**, ebselen and cisplatin (CDDP) were used as reference drugs. Besides tumour cells, the most active compounds were also tested against the non-tumour cell line BJ-hTert (fibroblasts). In both cases, the NCI protocol, with minor modifications [54], was followed; selected data, including the selectivity index (S.I.) can be found in Table 3 and full data in Supporting Information (Table S1).

#### 2.2.3. P-glycoprotein Assay

This assay allows prediction of whether a certain chemotherapeutic agent could develop chemoresistance when behaving as a P-gp substrate [55], one of the most common efllux-pumps used for the extrusion of cell xenobiotics. For that purpose, a cell line-based assay was used, in particular the lung cancer cell line in its wild type (SW1573), and its variant overexpressing P-gp (Sw1573/Pgp) [56]. Moreover, compounds were tested in the presence and in the absence of verapamil, which is known to inhibit P-gp [57]. Chemotherapeutic agents paclitaxel (PTX) and vinblastine (VB) are used herein as reference compounds. In this assay, the resistance factor (Rf) for a certain compound is defined as the ratio of GI_50_ in the cell line overexpressing P-gp and the wild one (Table 4), high values of Rf denoting that the compound behaves as a P-gp substrate, and thus, chemoresistance through this mechanism will probably take place.

## 3. Discussion

The role of ROS in cancer cells is highly debated nowadays; although they are known to be present at high levels during carcinogenesis and cancer progression, it is claimed that they have a dual activity [58]. Thus, on the one hand, they can facilitate cell proliferation by acting as signalling molecules, a capacity that is increased by rising ROS levels, and a concomitant improved antioxidant machinery; nevertheless, on the other hand, ROS can also provoke cell death by inducing apoptosis [59].

Free phenolic derivatives can scavenge free radicals upon reaction with free phenolic hydroxyl groups, to give quinone-type intermediates [33] (sacrificial antioxidants), converting ROS into non-radical species, like alcohols or water; in this sense, phenolic compounds act as chain-breaking or primary antioxidants, probably the most important family of natural compounds having such antioxidant mechanism. The antioxidant properties of organochalcogen, particularly organoselenium derivatives, are diverse, and therefore, they might be useful against different targets in oxidative stress, an activity elicited by their exceptional redox properties. Such activities include scavenging of free radicals [60], metal complexation [61], or mimicry of natural antioxidant enzymes for the removal of deleterious hydrogen peroxide and alkyl peroxides (e.g., GPx), the so-called bioinspired antioxidants [46]. Interestingly, diselenides and selenides can be substrates in vivo for mammalian thioredoxin reductase (TrxR), furnishing selenols/selenolates, stronger nucleophiles than their sulfur counterparts; as a result, the corresponding selenol has a stronger reducing power than isosteric thiol [62,63].

Concerning the antiradical properties, data depicted in Table 1 clearly show the influence of the length of the hydrocarbon chain of disulfides **8**‒**10** (See structures in Scheme 2). Thus, compound **9**, with a three-carbon length tether, was the best compound in the series (EC_50_ = 4.2 µM). The same tendency, although to a lower extent, was found for selenoisosters **15**, **16** (See structures in Scheme 2). Accordingly, it seems that the combination of a phenolic scaffold with a dichalcogenide motif provokes a synergic effect in the antiradical properties, leading to improved derivatives compared to reference compounds. Furthermore, the ebselen-dopamine hybrid **26** (See structure in Scheme 4) showed stronger antiradical activity compared with phenyl selenide **18** and selenocyanate **20** (See structures in Scheme 3). Replacement of the phenolic structure with a methylidene moiety led to a complete impairment of activity (compounds **39**, **46**, See structures in Scheme 5 and Scheme 6, respectively), indicating that the diselenide moiety lacks antiradical properties itself, but through a synergic effect, improves that of the phenolic motif; in fact, disulfide **9** exhibited a roughly three-fold increased activity compared to reference compounds (hydroxytyrosol and dopamine).

H_2_O_2_ is considered to be one of the most common ROS in living organisms, and in particular in tumor cells [64], being produced by the mitochondria-based respiratory chain cascade through a complex enzymatic machinery [65]. For that purpose, we also envisioned the possibility of analyzing the scavenging properties of compounds prepared herein against such ROS. In a similar way to what was observed in the antiradical assay, disulfide **9** (See structure in Scheme 2) was again the most potent antioxidant within the series, capable of scavenging roughly 80% of the initial H_2_O_2_ level. Although to a lower extent, diselenide **16** (See structure in Scheme 2) and ebselen analogue **26** (See structure in Scheme 4) were also remarkable examples, capable of scavenging roughly 50% of the ROS. Removal of the catechol functionality (compounds **4**, **39**, **46**, See structures in Scheme 1, Scheme 5 and Scheme 6) led to a complete loss of ROS scavenging properties.

This capacity for scavenging ROS could suggest a potential chemoprotective action of title compounds; as it can be observed, some of the sulfur-containing derivatives exhibited a similar or even a slightly better antioxidant profile than selenium-counterparts. Accordingly, it could be claimed that some of the sulfur derivatives prepared herein could act as good chemoprotective agents, with probably reduced toxicological concerns than isosteric selenium. However, when shifted to the antiproliferative activity, as below discussed, the sulfur derivatives showed a negligible activity, unlike selenium-containing compounds. Interestingly for some other selenoderivatives we have previously detected a pro-oxidant activity in the presence of tumour cells, with a significant increase in ROS levels, and thus, potentially a pro-apoptotic effect [13].

With regards to antiproliferative activity, derivatives prepared herein can be categorized into a series of families; the first of them is comprised of disulfides **4**, **8**‒**10** (See structures in Scheme 1 and Scheme 2), their selenium isosters **15**, **16** (See structures in Scheme 2), selenides **18**, **20** (See structures in Scheme 3) and ebselen analogue **26** (See structure in Scheme 4). All of them have in common some structural motifs: the presence of an unprotected catechol moiety, and the connection of the phenolic and organoselenium fragments via an amide tether. According to data depicted in Table 3 and Table S1, a series of valuable structure-activity relationships can be obtained; firstly, the length of the tether was found to be again crucial for the bioactivities, although with different behaviour for every family of compounds; thus, whereas disulfides **8** and **10**, with a 2- and 4-atom carbon tether, respectively, turned out to have negligible activities at the highest tested concentration (100 µM), derivative **9** (with a 3-carbon tether) exhibited a moderate activity, with GI_50_ values lower than 45 µM for 5 cell lines. 

Interestingly, isosteric replacement of sulfur with selenium furnished considerable stronger derivatives (**15**, **16**), again with a remarkable influence on the tether length; thus, diselenide **16**, with a longer tether, exhibited GI_50_ values lower than 6.0 µM for all the cell lines, although lacking selectivity (GI_50_ = 3.5 µM for fibroblasts). Moreover, the nature of the organoselenium motif was also found to clearly influence the activity, as selenides **18**, **20** and ebselen analogue **26** only showed moderate antiproliferative properties.

With these data in hand, we attempted to improve both, the activity and selectivity by accomplishing structural modifications. For that purpose, we decided to install a methylidene acetal protecting group on the phenolic scaffold, and to eliminate the amide tether, giving access to derivatives **35**‒**41** (See structures in Scheme 5). Remarkably, such structural modification afforded low micromolar‒submicromolar activities for selenocyanates **35**, **36** and diselenides **39**, **40**. Again, distance between both pharmacophores, the protected phenolic residue, and the organoselenium motif were found to be crucial structural elements, as diselenide **41**, unlike its counterparts **39**, **40**, was found to be only a moderate antiproliferative agent. Therefore, activity increased from a 2 to 3-carbon linker, but was clearly diminished for the four-carbon structure (**41**). It is also important to highlight that a rather good increase in selectivity was achieved for selenocyanate **36** (4‒13-fold), and particularly for diselenide **40** (up to 32-fold).

We next designed a family containing the key pharmacophoric motifs present in the previous two families of derivatives, giving access to selenocyanate **45** and diselenide **46** (See structures in Scheme 6). In both cases, a methylidene acetal, and an amide-type tether are present simultaneously in the molecule. Such compounds turned out to be the most potent compounds of the series, with activities in the submicromolar range for most of the tested cell lines (0.36–1.6 µM for **45** and 0.12–0.30 µM for **46**), although reduced selectivity in comparison to **40**.

Remarkably, diselenide **46** exhibited up to a 104-fold increase in activity compared to CDDP in multidrug resistant cell lines (T-47D, WiDr), showing also a selectivity index of 3‒8 compared to non-tumor cell lines.

**Figure 2:**
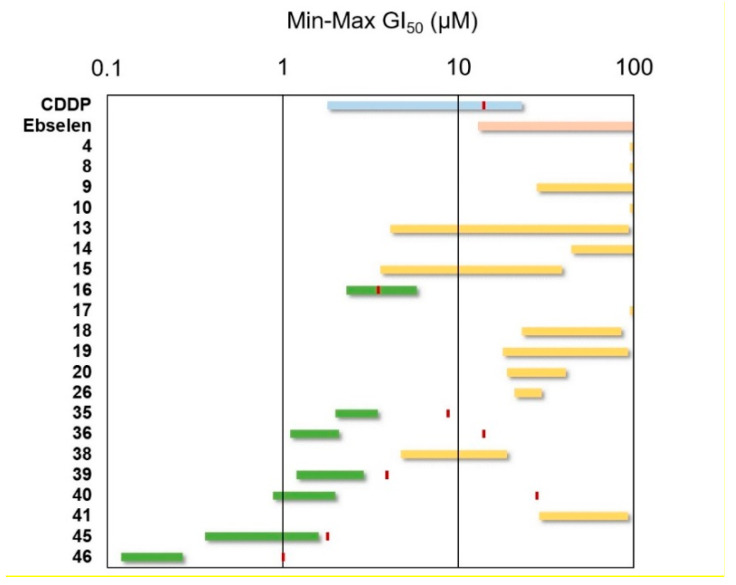
GI_50_ range plot of active compounds against human solid tumor cell lines and GI_50_ values against human fibroblasts (red bars). Cisplatin (CDDP) was used as reference anticancer drug. Ebselen was used as reference parental drug. Green colour indicates GI_50_ < 10 µM for all cell lines. Yellow colour indicates GI_50_ > 10 µM for some or all cell lines.

Accordingly, in view of GI_50_ plot range (Figure 2), the three most potent derivatives were found to be **40**, **45** and **46**, now with the longest tether, the former exhibiting the best profile in terms of selectivity. The influence of the free or protected phenolic scaffold can be clearly analysed by comparing the activities of **16** (free catechol) and **46** (acetal-containing derivative), both with the same tether and the same selenium functionality (diselenide). Although toxicity towards non-tumour cells is slightly increased, the potency against tumour cell lines is improved up to 30-fold, so furnishing a better overall selectivity index for protected **46**. Probably, a reduced polarity of **46** in comparison to **16** enables transportation through cell membranes, thus, improving the bioavailability of the compound. Some of the least cytotoxic compounds towards Bj-h-Tert lack the amide functionality, so it seems that removal of such moiety might contribute to the improvement of the selectivity index.

So, with all data in hand, diselenide **40** can be considered as the lead compound of the series; although it is not featured with the highest potency, GI_50_ values are still rather good, within the low micromolar-submicromolar concentration, particularly interesting against the two multidrug resistant cell lines (T-47D, breast) and WiDr (colon). Moreover, the selectivity index (SI) was found to be 14‒32, thus showing a clear improvement of the antiproliferative profile in comparison to the reference drug (cisplatin), whose reduced selectivity is partially responsible for its numerous side-effects.

It is also interesting to mention that Desai and co-workers designed a selenocyanate and a diselenide as SAHA analogues [28], an hydroxamic acid that behaves as a strong histone deacetylase inhibitor, enzymes overexpressed in certain tumours for regulating cancer progression [66]. Such selenoderivatives, more potent histone deacetylase inhibitors than parent SAHA, were reported to be particularly active against melanoma [67], and it was hypothesized that both functionalities could be reduced under cell conditions to give a transient selenide that binds histone deacetylase [68].

In connection with this fact, it is important to mention that the most relevant antiproliferative properties found herein are coming from selenocyanates and diselenides; dialkyl diselenides have been found to exert pro-oxidant properties at low concentrations [69]. Besides targeting ROS level, it could be hypothesized that title compounds could be reduced [63] in the tumour microenvironment, giving a transient selenol/selenolate that might inhibit histone deacetylases in a similar way found for the Se-analogues of SAHA.

One major challenge when developing chemotherapeutic agents for the treatment of cancer progression is to avoid chemoresistance. Although there are many mechanisms of resistance to drugs, like changes in mitochondria, induced DNA-repair, oncogenes or tumor suppressor genes, among others, as indicated previously, a classical one is comprised of transporter pumps (e.g., P-glycoproteins), that act by extruding xenobiotics from the cell in an ATP-dependent manner; such glycoproteins are overexpressed in a number of tumors. Therefore, knowledge if chemotherapeutic agents are P-gp substrates or not becomes a crucial aspect in order to predict chemoresistance mediated by these efflux pumps [70]. Although they do not constitute the only mechanism developed by cancer cells to eliminate drugs, it is one of the most studied ones, and now considered to be one the key target to overcome chemoresistance [71], either by inhibition or by evasion; many of the most widely-used chemotherapeutic agents (e.g., doxorubicin, etoposide, paclitaxel, etc.) are susceptible to P-gp-mediated efflux.

Table 4 depicts GI_50_ values after a 48 h-exposure of compounds with wild SW1573 cell line, or the one overexpressing P-gp, with (w) or without (w/o) the presence of the P-gp inhibitor verapamil. Overall, the low values found for Rf denote that our compounds do not act as substrates for P-gp and thus cannot develop chemoresistance via this pump efflux mechanism, in clear contrast with the reference compounds used herein.

## 4. Experimental Section

### 4.1. Materials and Methods

#### 4.1.1. General Procedures

^1^H (300.1 and 500.1 MHz) and ^13^C (75.5 and 125.7 MHz) NMR spectra were recorded at 25 °C on a Bruker Avance 300, or on a Bruker Avance III 500 MHz spectrometer; the latter, equipped with a TCI cryoprobe). 2D NMR experiments (COSY, HSQC) were used for the assignment of ^1^H and ^13^C signals. Mass spectra (ESI) were recorded on a QExactive mass spectrometer. TLCs were performed on aluminium pre-coated sheets (E. Merck silica gel 60 F_254_); spots were visualized by UV light, and by charring with 1% phosphomolybdic acid in EtOH, 1.5% vanillin in EtOH containing 1% of H_2_SO_4_, or with 0.3% ninhydrin in EtOH. Column chromatography was performed using E. Merck silica gel 60 (40–63 μm). The antioxidant assays were performed in a Hitachi U-2900 spectrophotometer with a thermostated cuvette holder, using PS cuvettes (1 cm × 1 cm × 4.5 cm).

#### 4.1.2. Antioxidant Assays

##### DPPH Assay

Reported methodology [52] was used with minor modifications. To a 60 µM methanolic DPPH solution (1.17 mL), the antioxidant solution (5–7 different solutions, 30 µL) or solvent (control, 30 µL) were added. After 30 min. of incubation in the dark, absorbance was measured at 515 nm, and EC_50_ values were calculated.

##### H_2_O_2_ Scavenging Assay

Reported methodology [53] was used with minor modifications [60].

#### 4.1.3. Antiproliferative Activity

The in vitro antiproliferative activity was assayed using minor modifications of the protocol of the National Cancer Institute (NCI) of the United States against the six human solid tumor cell and the non-tumor cell lines tested [54]. 

#### 4.1.4. Statistical Analysis

For antioxidant assays, all tests were run in triplicate. Values are expressed as the confidence interval, which was calculated for *p* = 0.95 using the Student’s *t*-distribution. For antiproliferative assays, the median and standard deviation (SD) for 2‒3 measurements were calculated.

### 4.2. Chemistry

#### 4.2.1. [4”-(2”‘-Hydroxyethyl)-1”,2”-phenylene]-4,4’-dithiobisbutyrate (**4**)

To a solution of 2-(3′,4′-dihydroxyphenyl)ethanol (**1**) (50.2 mg, 0.33 mmol) in anhydrous DMF (4 mL) were added 4,4’-dithiobisbutyric acid (26.2 mg, 0.11 mmol), PyBOP (114.5 mg, 0.22 mmol) and anhydrous Et_3_N (60 μL, 0.43 mmol). The mixture was stirred in the dark, at rt and under Ar overnight. The crude reaction was then concentrated to dryness and the residue was purified by column chromatography (cyclohexane→1:1 cyclohexane‒EtOAc) to give **4**. Yield: 11.8 mg, 30%; *R_F_* 0.62 (1:1 cyclohexane‒EtOAc); ^1^H-NMR (300 MHz, CD_3_OD) δ 7.16 (m, 2H, Ar‒H), 7.13 (m, 1H, Ar‒H), 3.77 (t, 2H, *J*_H,H_ = 6.8 Hz, C*H*_2_OH), 2.88‒2.77 (m, 10H, 5CH_2_), 2.10 (m, 4H, 2CH_2_S) ppm; ^13^C-NMR (75.5 MHz, CD_3_OD) δ 172.1, 172.0 (2CO), 143.2, 141.8 (C-1′’, C-2′’), 139.7 (C-4′’), 128.2 (C-5′’), 125.0, 124.3 (C-3′’, C-6′’), 63.7 (CH_2_OH), 39.5, 39.4 (x2), 33.1 (x2) (5CH_2_), 25.1, 25.0 (C*H*_2_CH_2_S) ppm; HRESI‒MS calcd for C_16_H_20_NaO_5_S_2_ ([M+Na]^+^): 379.0644, found: 379.0641.

#### 4.2.2. General Method for the Preparation of Disulfides **8‒10** and Diselenides **15,16**

To a solution of the corresponding dithiobiscarboxylic acid (0.21 mmol) in anhydrous DMF (5 mL) at 0 °C were added dopamine hydrochloride **5** (80.0 mg, 0.42 mmol), PyBOP (219.6 mg, 0.42 mmol) and anhydrous Et_3_N (86 μL, 0.62 mmol). The mixture was stirred in the dark, at rt and under Ar overnight. Then, the crude reaction was concentrated to dryness and the corresponding residue was purified by column chromatography using the eluant indicated in each case.

##### 2,2′-Dithiobis{N-[2′’-(3′’’,4′’’-dihydroxyphenyl)ethyl]acetamide} (**8**)

Dithiodiacetic acid **6** (38.3 mg. 0.21 mmol) was used. Column chromatography (30:1→ 25:1 EtOAc‒MeOH) afforded **8** as an oil. Yield: 89.5 mg, 94%; *R_F_* 0.58 (6:1 EtOAc‒MeOH); ^1^H-NMR (300 MHz, CD_3_OD) δ 6.68 (d, 1H, *J*_5′’’,6′’’_ = 7.9 Hz, H-5′’’), 6.67 (d, 1H, *J*_2′’’,6′’’_ = 1.6 Hz, H-2′’’), 6.55 (dd, 1H, H-6′’’), 3.39 (t, 2H, *J*_H,H_ = 7.1 Hz, CH_2_N), 3.37 (s, 2H, CH_2_S), 2.67 (t, 2H, CH_2_Ar) ppm; ^13^C-NMR (75.5 MHz, CD_3_OD) δ 171.1 (CO), 146.1, 144.6 (C-3′’’, C-4′’’), 131.9 (C-1′’’), 121.1 (C-6′’’), 116.9, 116.4 (C-2′’’, C-5′’’), 42.9, 42.7 (CH_2_N, CH_2_‒Ar), 35.7 (CH_2_S) ppm; HRESI‒MS calcd for C_20_H_24_N_2_NaO_6_S_2_ ([M+Na]^+^): 475.0968, found: 475.0964.

##### 3,3′-Dithiobis{*N*-[2′’-(3′’’,4′’’-dihydroxyphenyl)ethyl]propanamide} (**9**)

3,3′-Dithiobispropionic acid **7** (44.2 mg, 0.21 mmol) was used. Column chromatography (50:1→25:1 EtOAc‒MeOH) afforded **9** as an oil. Yield: 80.2 mg, 79%; *R_F_* 0.69 (6:1 EtOAc‒MeOH); ^1^H-NMR (500 MHz, CD_3_OD) δ 6.68 (d, 1H, *J*_5′’’,6′’’_ = 8.0 Hz, H-5′’’), 6.64 (d, 1H, *J*_2′’’,6′’’_ = 2.0 Hz, H-2′’’), 6.52 (dd, 1H, H-6′’’), 3.34 (t, 2H, *J*_H,H_ = 7.1 Hz, CH_2_N), 2.90 (t, 2H, *J*_H,H_ = 7.2 Hz, CH_2_S), 2.63 (t, 2H, CH_2_Ar), 2.55 (t, 2H, C*H*_2_CH_2_S) ppm; ^13^C-NMR (125.7 MHz, CD_3_OD) δ 173.6 (CS), 146.2, 144.7 (C-3′’’, C-4′’’), 132.0 (C-1′’’), 121.1 (C-6′’’), 116.9, 116.3 (C-2′’’, C-5′’’), 42.3 (CH_2_N), 36.5 (CH_2_Ar), 35.9 (*C*H_2_CH_2_S), 35.2 (CH_2_S) ppm; HRESI‒MS calcd for C_22_H_28_N_2_NaO_6_S_2_ ([M+Na]^+^): 503.1281, found: 503.1276.

##### 4,4′-Dithiobis{*N*-[2′’-(3′’’,4′’’-dihydroxyphenyl)ethyl)]butanamide} (**10**)

4,4′-Dithiobisbuthyric acid **2** (50.3 mg, 0.21 mmol) was used. Column chromatography (20:1→1:1 CH_2_Cl_2_‒MeOH and EtOAc) afforded **10**. Yield: 85.3 mg, 80%; *R_F_* 0.61 (5:1 CH_2_Cl_2_‒MeOH). ^1^H-NMR (300 MHz, CD_3_OD) δ 6.68 (d, 1H, *J*_5′’’,6′’’_ = 8.0 Hz, H-5′’’), 6.64 (d, 1H, *J*_2′’’,6′’’_ = 2.0 Hz, H-2′’’), 6.52 (dd, 1H, H-6′’’), 3.34 (t, 2H, *J*_H,H_ = 7.0 Hz, CH_2_N), 2.63 (t, 4H, *J*_H,H_ = 7.0 Hz, CH_2_Ar, CH_2_S), 2.26 (t, 2H, *J*_H,H_ = 7.2 Hz, CH_2_CO), 1.94 (quint, 2H, C*H*_2_CH_2_S) ppm; ^13^C-NMR (125.7 MHz, CD_3_OD) δ 175.2 (CO), 146.2, 144.7 (C-3′’’, C-4′’’), 132.0 (C-1′’’), 121.1 (C-6′’’), 116.8, 116.4 (C-2′’’, C-5′’’), 42.1 (CH_2_N), 38.7, 35.9, 35.5 (CH_2_-Ar, CH_2_CO, CH_2_S), 26.4 (*C*H_2_CH_2_S) ppm; HRESI‒MS calcd for C_24_H_32_N_2_NaO_6_S_2_ ([M+Na]^+^): 531.1594, found: 531.1586. 

##### 3,3′-Diselenobis{*N*-[2′’-(3′’’,4′’’-dihydroxyphenyl)ethyl]propanamide} (**15**)

3,3′-Diselenobispropanoic acid **13** (63.8 mg, 0.21 mmol) was used Column chromatography (30:1→20:1 EtOAc‒MeOH) afforded **15**. Yield: 74.8 mg, 62%; *R_F_* 0.71 (5:1 EtOAc‒MeOH); ^1^H-NMR (300 MHz, CD_3_OD) δ 6.68 (d, 1H, *J*_5′’’,6′’’_ = 8.0 Hz, H-5′’’), 6.65 (d, 1H, *J*_2′’’,6′’’_ = 2.0 Hz, H-2′’’), 6.53 (dd, 1H, H-6′’’), 3.35 (brt, 2H, CH_2_N), 3.08 (t, 2H, *J*_H,H,_ = 7.3 Hz, CH_2_Se), 2.64, 2.63 (2t, 2H each, C*H*_2_CH_2_Se, CH_2_Ar) ppm; ^13^C-NMR (75.5 MHz, CD_3_OD) δ 174.0 (CO), 146.0, 144.5 (C-3′’’, C-4′’’), 132.0 (C-1′’’), 121.1 (C-6′’’), 116.8, 116.3 (C-2′’’, C-5′’’), 42.2 (CH_2_N), 38.1 (CH_2_Ar), 35.8, 25.4 (C*H*_2_CH_2_S, CH_2_C*H*_2_S) ppm; HRESI‒MS calcd for C_22_H_28_N_2_NaO_6_^80^Se_2_ ([M+Na]^+^): 599.0170, found: 599.0167. 

##### 4,4′-Diselenobis{*N*-[2′’-(3′’’,4′’’-dihydroxyphenyl)ethyl]butanamide} (**16**)

4,4′-Diselenobisbutyric acid 14 (70.6 mg, 0.21 mmol) was used. Column chromatography (50:1→30:1 EtOAc‒MeOH) afforded 16. Yield: 97.8 mg, 77%; ^1^H-NMR (300 MHz, CD_3_OD) δ 6.68 (d, 1H, J_5′’’,6′’’_ = 8.0 Hz, H-5′’’), 6.64 (d, 1H, J_2′’’,6′’’_ = 2.0 Hz, H-2′’’), 6.52 (dd, 1H, H-6′’’), 3.35 (brt, 2H, CH_2_N), 2.83 (t, 2H, J_H,H,_ = 7.4 Hz, CH_2_Se), 2.63 (t, 2H, J_H,H,_ = 7.4 Hz, CH_2_), 2.25 (t, 2H, J_H,H,_ = 7.1 Hz, CH_2_), 1.99 (quint, 2H, CH_2_-CH_2_-CH_2_) ppm; ^13^C-NMR (125.7 MHz, CD_3_OD) δ 175.1 (CO), 146.1, 144.6 (C-3′’’, C-4′’’), 132.0 (C-1′’’), 121.1 (C-6′’’), 116.8, 116.3 (C-2′’’, C-5′’’), 42.1 (CH_2_N), 36.5, 35.8 (CH_2_Ar, CH_2_CO), 29.5, 28.2 (CH_2_Se, CH_2_‒CH_2_Se) ppm; HRESI‒MS calcd for C_24_H_32_N_2_NaO_6_^80^Se_2_ ([M+Na]^+^): 627.0483, found: 627.0474.

#### 4.2.3. *N*-[2′-(3′’,4′’-Dihydroxyphenyl)ethyl]-3-phenylselenopropanamide (**18**)

To a solution of 3-phenylselenopropanoic acid 17 (96.4 mg, 0.42 mmol) in anhydrous DMF (6 mL) were added dopamine hydrochloride 5 (80.0 mg, 0.42 mmol), PyBOP (219.6 mg, 0.42 mmol) and anhydrous Et_3_N (86 μL, 0.62 mmol). The corresponding mixture was stirred in the dark, at rt and under Ar overnight. Then, the crude reaction was concentrated to dryness, and the residue was purified by column chromatography (cyclohexane→1:8 cyclohexane‒EtOAc). Yield: 123.8 mg, 81%; R_F_ 0.71 (EtOAc); ^1^H-NMR (300 MHz, CD_3_OD) δ 7.52‒7.48 (m, 2H, Ar‒H), 7.30‒7.23 (m, 3H, Ar‒H), 6.68 (d, 1H, J_5′’,6′’_ = 8.0 Hz, H-5′’), 6.64 (d, 1H, J_2′’,6′’_ = 2.0 Hz, H-2′’), 6.52 (dd, 1H, H-6′’), 3.31 (t, 2H, J_H,H_ = 7.5 Hz, CH_2_N), 3.09 (t, 2H, CH_2-_Ar), 2.62 (t, 2H, J_H,H_ = 7.2 Hz, CH_2_), 2.54 (t, 2H, CH_2_) ppm; ^13^C-NMR (75.5 MHz, CD_3_OD) δ 174.1 (CO), 146.1, 144.7 (C-3′’, C-4′’), 133.7 (x2) (Ar‒C_o_), 131.9 (C-1′’), 130.9 (Ar‒C_ipso_), 130.1 (x2) (Ar‒C_m_), 128.0 (Ar‒C_p_), 121.1 (C-6′’), 116.8, 116.3 (C-2′’, C-5′’), 42.2 (CH_2_N), 37.7, 35.8, 23.3 (CH_2_Ar, CH_2_CO, CH_2_Se) ppm; ^77^Se-NMR (95 MHz, CDCl_3_) δ 306.16 ppm; HRESI‒MS calcd for C_17_H_19_NNaO_3_^80^Se ([M+Na]^+^): 388.0422, found: 388.0421. 

#### 4.2.4. 3-Selenocyanatopropanoic acid (**19**)

To a solution of 3-bromopropanoic acid (100.0 mg, 0.65 mmol) in anhydrous DMF (6 mL) was added potassium selenocyanate (376.7 mg, 2.61 mmol, 4.0 equiv.). The corresponding mixture was stirred overnight at 60 °C, under inert atmosphere (Ar) and in the dark. Then, it was concentrated to dryness, and the residue was partitioned between water (20 mL) and CH_2_Cl_2_ (3 × 15 mL). The combined organic fractions were dried over Na_2_SO_4_, filtrated and the filtrate was concentrated to dryness to give 19 as an oil, which was directly used for the next step without any further purification. Yield: 82.4 mg, 71%; ^1^H-NMR (300 MHz, CDCl_3_) δ 7.33 (brs, 1H, COOH), 3.24 (t, 2H, J_H,H_ = 6.6 Hz, CH_2_‒COOH), 3.06 (t, 2H, CH_2_SeCN); ^13^C-NMR (75.5 MHz, CDCl_3_) δ 176.8 (COOH), 101.9 (NCSe), 35.0 (CH_2_COOH), 22.9 (CH_2_SeCN) ppm; HRESI‒MS calcd for C_4_H_5_NNaO_2_^80^Se ([M+Na]^+^): 201.9378, found: 201.9380.

#### 4.2.5. *N*-[2′-(3′’,4′’-Dihydroxyphenyl)ethyl]-3-selenocyanatopropanamide (**20**)

To a solution of 3-selenocyanatopropanoic acid **19** (27.0 mg, 0.15 mmol) in anhydrous DMF (2 mL) were added dopamine hydrochloride 5 (28.6 mg, 0.15 mmol), PyBOP (78.4 mg, 0.15 mmol) and anhydrous Et_3_N (31 μL, 0.22 mmol, 1.5 equiv.). The corresponding mixture was stirred in the dark, at rt and under Ar overnight. Then, the crude reaction was concentrated to dryness, and the residue was purified by column chromatography (20:1→3:1 CH_2_Cl_2_‒MeOH and cyclohexane→1:4 cyclohexane‒EtOAc). Yield: 15.9 mg, 34%; R_F_ 0.70 (EtOAc); ^1^H-NMR (500 MHz, CD_3_OD) δ 6.68 (d, 1H, J_5′’,6′’_ = 8.0 Hz, H-5′’), 6.64 (d, 1H, J_2′’,6′’_ = 2.1 Hz, H-2′’), 6.52 (dd, 1H, H-6′’), 3.34 (t, 2H, J_H,H_ = 7.2 Hz, CH_2_N), 3.25 (t, 2H, J_H,H_ = 6.7 Hz, CH_2_CO), 2.80 (t, 2H, CH_2_Se), 2.64 (t, 2H, CH_2_Ar) ppm; ^13^C-NMR (125.7 MHz, CD_3_OD) δ 173.1 (CO), 146.3, 144.8 (C-3′’, C-4′’), 131.9 (C-1′’), 121.0 (C-6′’), 116.8, 116.4 (C-2′’, C-5′’), 105.4 (SeCN), 42.4 (CH_2_N), 36.7, 35.8 (CH_2_Ar, CH_2_CO), 25.6 (CH_2_Se) ppm; HRESI‒MS calcd for C_12_H_14_N_2_NaO_3_^80^Se ([M+Na]^+^): 337.0062, found: 337.0058. 

#### 4.2.6. *N*-[2′-(3′’,4′’-Dihydroxyphenyl)ethyl]-1,2-benzisoselenazole-3(2*H*)-one (**26**)

A solution of 2,2′-diselenobisbenzoic acid **24** (128.0 mg, 0.32 mmol) and anhydrous DMF (63 µL, 0.81 mmol) in SOCl_2_ (2 mL, 27.6 mmol) was refluxed under inert atmosphere and in the dark for 2 h. Then, the crude reaction was concentrated to dryness to give 2-(chloroseleno)benzoyl chloride 25 as a brown oil, which was directly used for the next step without any further purification. To a solution of crude 25 in anhydrous DMF (1 mL), a solution of dopamine hydrochloride (60.9 mg, 0.32 mmol) and Et_3_N (0.25 mL, 1.79 mmol) in anhydrous DMF (3 mL) was added. The corresponding mixture was stirred at rt, under Ar and in the dark overnight. Then, it was concentrated to dryness and the residue was purified by column chromatography (40:1 EtOAc‒MeOH) to give 26 as a yellow oil. Yield: 32 mg, 30%; R_F_ 0.68 (6:1 EtOAc‒MeOH); ^1^H-NMR (300 MHz, CD_3_OD) δ 7.91 (ddd, 1H, J_4,5_ = 7.9 Hz, J_4,6_ = 1.3 Hz, J_4,7_ = 0.6 Hz, H-4), 7.85 (dt, 1H, J_6,7_ = 8.0 Hz, J_5,7_ = 0.8 Hz, H-7), 7.57 (ddd, 1H, J_5,6_ = 7.2 Hz, H-5), 7.41 (ddd, 1H, H-6), 6.68 (d, 1H, J_5′’,6′’_ = 7.9 Hz, H-5′’), 6.67 (d, 1H, J_2′’,6′’_ = 2.3 Hz, H-2′’), 6.56 (dd, 1H, H-6′’), 3.99 (t, 2H, J_H,H_ = 7.0 Hz, CH_2_N), 2.85 (t, 2H, CH_2_Ar) ppm; ^13^C-NMR (125.7 MHz, CD_3_OD) δ 169.1 (CO), 146.3, 145.1 (C-3′’, C-4′’), 141.3 (C-7a), 132.9 (Ar‒C), 131.0 (C-3a), 128.8, 128.7, 126.9, 126.1 (Ar‒C), 121.3 (C-6′’), 117.1, 116.5 (C-2′’, C-5′’), 47.4 (CH_2_N), 36.6 (CH_2_Ar) ppm; HRESI‒MS calcd for C_15_H_13_NNaO_3_^80^Se ([M+Na]^+^): 357.9953, found 357.9949. 

#### 4.2.7. General Procedure for the Synthesis of Selenocyanates **35**‒**37**

To a solution of the corresponding ω-(3′,4′-methylenedioxyphenyl)alkyl halide **32**‒**34** (0.83 mmol) in DMF (5 mL) was added potassium selenocyanate (3.0 equiv.). The corresponding mixture was stirred under Ar and in the dark, at rt, 13h for **35** and at 75 °C, 5 h for **36**, **37**. After that, the crude was concentrated to dryness and the residue was partitioned between CH_2_Cl_2_ (20 mL) and H_2_O (20 mL); the aqueous layer was further extracted with CH_2_Cl_2_ (3 × 10 mL). The combined organic fractions were dried over Na_2_SO_4_, filtered, and the filtrate was concentrated to dryness; the residue was purified by column chromatography using the eluant indicated in each case.

##### (3′,4′-Methylenedioxyphenyl)methyl selenocyanate (**35**)

(3′,4′-methylenedioxyphenyl)methyl chloride **32** (141.6 mg, 0.83 mmol) was used. Column chromatography (10:1 cyclohexane‒EtOAc) gave **35** as a white solid. Yield: 172.4 mg (87%); *R_F_* 0.29 (9:1 cyclohexane‒EtOAc); ^1^H-NMR (300 MHz, CDCl_3_) δ 6.85‒6.81 (m, 2H, H-2, H-6), 6.77 (d, 1H, *J*_5,6_ = 8.4 Hz, H-5), 5.98 (s, 2H, OCH_2_O), 4.26 (s, 2H, CH_2_Se) ppm; ^13^C-NMR (125.7 MHz, CDCl_3_) δ 148.3, 148.2 (C-3, C-4), 129.0 (C-1), 123.0 (C-6), 109.3 (C-2), 108.8 (C-5), 102.0 (SeCN), 101.6 (OCH_2_O), 33.5 (CH_2_Se) ppm; HRESI‒MS calcd for C_9_H_7_NNaO_2_^80^Se ([M+Na]^+^): 263.9534, found: 263.9527.

##### 2-(3′,4′-Methylendioxyphenyl)ethyl selenocyanate (**36**)

2-(3′,4′-Methylenedioxyphenyl)ethyl bromide **33** (190 mg, 0.83 mmol) was used. Column chromatography (5:1 cyclohexane‒EtOAc) afforded **36**. Yield: 192 mg, 91%; *R_F_* 0.66 (6:1 cyclohexane‒EtOAc); ^1^H-NMR (300 MHz, CDCl_3_) δ 6.76 (d, 1H, *J*_5′,6′_ = 7.7 Hz, H-5′), 6.68‒6.65 (m 2H, H-2′, H-6′), 5.94 (s, 2H, OCH_2_O), 3.23, 3.10 (2brt, 2H each, *J*_H,H_ = 7.0 Hz, CH_2_-CH_2_) ppm; ^13^C-NMR (75.5 MHz, CDCl_3_) δ (ppm): 148.0, 146.8 (C-3′, C-4′), 132.3 (C-1′), 121.7 (C-6′), 108.9, 108.6 (C-2′, C-5′), 101.5 (CN), 101.1 (OCH_2_O), 36.6 (CH_2_Ar), 30.8 (CH_2_Se); HRESI‒MS calcd for C_10_H_9_NNaO_2_^80^Se ([M+Na]^+^): 277.9691, found 277.9687. 

##### 3-(3’,4’-Methylenedioxyphenyl)propyl selenocyanate (**37**)

3-(3’,4’-Methylenedioxyphenyl)propyl bromide **34** (201.5 mg 0.83 mmol) was used. Column chromatography (6:1 cyclohexane‒EtOAc) gave **37**. Yield: 160.1 mg, 72%; *R*_F_ 0.34 (6:1 cyclohexane‒EtOAc). ^1^H-NMR (300 MHz, CDCl_3_) δ 6.74 (d, 1H, *J*_5′,6′_ = 7.9 Hz, H-5′), 6.67 (brd, 1H, *J*_2′,6′_ = 1.7 Hz, H-2′), 6.62 (brdd, 1H, H-6′), 5.93 (s, 2H, OCH_2_O), 3.01 (t, 2H, *J*_H,H_ = 7.2 Hz, CH_2_Se ), 2.70 (t, 2H, *J*_H,H_ = 7.1 Hz, CH_2_Ar), 2.19 (quint, 2H, C*H*_2_-CH_2_Se) ppm; ^13^C-NMR (125.7 MHz, CDCl_3_) δ 148.0, 146.3 (C-3′, C-4′), 133.7 (C-1′), 121.5 (C-6′), 109.0, 108.5 (C-2′, C-5′), 101.4 (SeCN), 101.1 (OCH_2_O), 34.7 (CH_2_Ar), 32.4 (*C*H_2_-CH_2_Se), 28.7 (CH_2_Se) ppm; ^77^Se-NMR (95 MHz, CDCl_3_) δ 207.57 ppm; HRESI‒MS calcd for C_11_H_11_NNaO_2_^80^Se ([M+Na]^+^): 291.9847, found 291.9839. 

#### 4.2.8. 1-Benzylseleno-2-(3’, 4’-methylenedioxyphenyl)ethane (**38**)

To a solution of 2-(3′,4′-methylenedioxyphenyl)ethyl selenocyanate **36** (129.1 mg, 0.51 mmol) and benzyl bromide (0.24 mL, 2.02 mmol, 4.0 equiv.) in MeOH (5 mL) at 0 °C, was slowly added NaBH_4_ (21.1 mg, 0.56 mmol, 1.1 equiv.). The resulting mixture was stirred at 0 °C, under inert atmosphere (N_2_) and in the dark for 2 h. Then, the crude reaction was concentrated to dryness and the residue was partitioned between EtOAc (8 mL) and sat. aq. NH_4_Cl (8 mL). The water layer was further washed with EtOAc (3x5 mL); the combined organic fractions were dried over MgSO_4_, filtered and concentrated to dryness. The residue was purified by column chromatography (cyclohexane) to give **38**. Yield: 72.2 mg, 44%; *R_F_* 0.76 (6:1 ciclohexane‒EtOAc); ^1^H-NMR (300 MHz, (CD_3_)_2_CO) δ 7.36‒7.20 (m, 5H, Ar-H), 6.74 (d, 1H, *J*_5′,6′_ = 7.9 Hz, H-5′), 6.71 (d, 1H, *J*_2′,6′_ = 1.5 Hz, H-2′) 6.65 (d, 1H, H-6′), 5.93 (s, 2H, OCH_2_O), 3.82 (s, 2H, C*H*_2_Ph), 2.86‒2.65 (m, 4H, CH_2_-CH_2_) ppm; ^13^C-NMR (75.5 MHz, (CD_3_)_2_CO) δ 148.6, 146.9 (C-3′, C-4′), 140.8 (Ar-C*_ipso_*), 136.3 (C-1′), 129.8, 129.2 (Ar-C*_o_*, Ar-C*_m_*) 127.3 (Ar-C*p*), 122.1 (C-6′), 109.6, 108.8 (C-2′, C-5′), 101.7 (OCH_2_O), 37.4 (CH_2_Ar), 27.1 (CH_2_Se), 25.7 (CH_2_Ph) ppm; ^77^Se-NMR (95 MHz, CDCl_3_) δ 262.17 ppm; HRESI‒MS calcd for C_16_H_16_NaO_2_^80^Se ([M+Na]^+^): 343.0208, found 343.0200. 

#### 4.2.9. General Procedure for the Preparation of Diselenides **39**‒**41**

To a solution of ω-(3’, 4’-methylenedioxyphenyl)alkyl selenocyanates **35**‒**37** (0.69 mmol) in absolute EtOH (7 mL) at 0 °C was slowly added NaBH_4_ (13.5 mg, 0.36 mmol). The corresponding mixture was stirred for 15 h at rt. The crude reaction was concentrated to dryness and the residue was purified by column chromatography using the eluant indicated in each case. 

##### Bis[(3’, 4’-methylenedioxyphenyl)methyl]diselenide (**39**)

(3′,4′-Methylenedioxyphenyl)methyl selenocyanate 35 (167 mg, 0.69 mmol) was used. Column chromatography (cyclohexane→4:1 cyclohexane‒EtOAc) gave 39. Yield: 87 mg, 59%; R_F_ 0.64 (4:1 cyclohexane‒EtOAc); ^1^H-NMR (300 MHz, CDCl_3_) δ 6.74 (d, 1H, J_5′,6′_ = 7.7 Hz, H-5′), 6.74 (brd, 1H, J_2′,6′_ = 1.6 Hz, H-2′), 6.69 (brdd, 1H, H-6′), 5.94 (s, 2H, OCH_2_O), 3.84 (s, 2H, CH_2_Se) ppm; ^13^C-NMR (125.7 MHz, CDCl_3_) δ 147.8, 146.9 (C-3′, C-4′), 132.9 (C-1′), 122.4 (C-6′), 109.5, 108.3 (C-2′, C-5′), 101.2 (OCH_2_O), 33.0 (CH_2_Se) ppm; HRESI‒MS calcd for C_16_H_14_NaO_4_^80^Se_2_ ([M+Na]^+^): 452.9115, found: 452.9109. 

##### Bis[2-(3′,4′-methylenedioxyphenyl)ethyl]diselenide (**40**)

2-(3′,4′-Methylenedioxyphenyl)ethyl selenocyanate 36 (177.7 mg, 0.69 mmol) was used. Column chromatography (40:1→30:1 cyclohexane‒EtOAc) gave 40 as a yellow oil. Yield: 61 mg, 39%; R_F_ 0.41 (6:1 cyclohexane‒EtOAc); ^1^H-NMR (300 MHz, CDCl_3_) δ 6.74 (d, 1H, J_5′,6′_ = 7.9 Hz, H-5′), 6.69 (brd, 1H, J_2′,6′_ = 1.4 Hz, H-2′), 6.65 (brdd, 1H, H-6′), 5.93 (s, 2H, OCH_2_O), 3.10 (brt, 2H, J_H,H_ = 7.5 Hz, CH_2_), 2.95 (brt, 1H, CH_2_) ppm; ^13^C-NMR (125.7 MHz, CDCl_3_) δ 147.8, 146.2 (C-3′, C-4′), 134.7 (C-1′), 121.5 (C-6′), 109.0, 108.4 (C-2′, C-5′), 101.0 (OCH_2_O), 37.4 (CH_2_Ar), 31.2 (CH_2_Se) ppm; HREI‒MS calcd for C_18_H_18_O_4_^80^Se_2_ ([M]^+^): 457.9530, found: 457.9527. 

##### Bis[3-(3’, 4’-methylenedioxyphenyl)propyl]diselenide (**41**)

3-(3’, 4’-Methylenedioxyphenyl)propyl selenocyanate **37** (184 mg, 0.69 mmol) was used. Column chromatography (cyclohexane→4:1 cyclohexane‒EtOAc) gave **41**. Yield: 121.3 mg, 73%; *R*_F_ 0.71 (4:1 cyclohexane‒EtOAc); ^1^H-NMR (300 MHz, CDCl_3_) δ 6.72 (d, 1H, *J*_5′,6′_ = 7.9 Hz, H-5′), 6.67 (brd, 1H, *J*_2′,6′_ = 1.4 Hz, H-2′), 6.62 (brdd, 1H, H-6′), 5.92 (s, 2H, OCH_2_O), 2.88 (t, 2H, *J*_H,H_ = 7.2 Hz, CH_2_Se), 2.63 (t, 2H, *J*_H,H_ = 7.2 Hz, CH_2_Ar), 2.01 (quint, 2H, C*H*_2_-CH_2_Se) ppm; ^13^C-NMR (125.7 MHz, CDCl_3_) δ 147.8, 145.9 (C-3′, C-4′), 135.2 (C-1′), 121.4 (C-6′), 109.0, 108.3 (C-2′, C-5′), 100.9 (OCH_2_O), 35.2 (CH_2_Ar), 32.7 (*C*H_2_-CH_2_Se), 29.1 (CH_2_Se) ppm; ^77^Se-NMR (95 MHz, CDCl_3_) δ 303.09 ppm; HRESI‒MS calcd for C_20_H_22_O_4_^80^Se_2_ ([M]^+^): 485.9849, found: 485.9837. 

#### 4.2.10. 1-Azido-2-(3′,4′-methylenedioxyphenyl)ethane (**42**)

A solution of 2-(3′,4′-methylenedioxyphenyl)ethyl bromide **33** (1.52 g, 6.64 mmol) and NaN_3_ (1.49 g, 22.9 mmol, 3.4 equiv.) in DMF (25 mL) was heated at 70 °C overnight. After that, the crude was partitioned between H_2_O (15 mL) and CH_2_Cl_2_ (3 × 20 mL); combined organic fractions were dried over MgSO_4_, filtered, and the filtrate was concentrated to dryness. The residue was purified by column chromatography (cyclohexane→1:4 cyclohexane‒Et_2_O) to give 42 as a colourless oil. Yield: 1.02 g, 80%. R_F_ 0.76 (1:4 cyclohexane‒Et_2_O); ^1^H-NMR (300 MHz, CDCl_3_) δ 6.66 (d, 1H, J_5′,6′_ = 8.0 Hz, H-5′), 6.61 (m, 1H, H-2′), 6.57 (m, 1H, H-6′), 5.84 (s, 2H, OCH_2_O), 3.36 (t, 2H, J_H,H_ = 7.2 Hz, CH_2_), 2.71 (t, 2H, CH_2_) ppm; ^13^C-NMR (75.5 MHz, CDCl_3_) δ 147.9, 146.5 (C-3′, C-4′), 131.9 (C-1′), 121.9 (C-6′), 109.2, 108.5 (C-2′, C-5′), 101.1 (OCH_2_O), 52.8 (CH_2_N_3_), 35.2 (CH_2_Ar) ppm; HRESI‒MS calcd for C_9_H_9_N_3_O_2_ [M]^+^: 191.0695, found: 191.0683. 

#### 4.2.11. *N*-[2′-(3′,4′-Methylenedioxyphenyl)ethyl]-4-selenocyanatobutanamide (**45**)

To a solution of 2-(3,4-methylendioxyphenyl)ethylamine **43** (194 mg, 1.17 mmol) in anhydrous DMF (3 mL), were added PyBOP (630 mg, 1.21 mmol), 4-selenocyanatobutanoic acid 44 (231 mg, 1.20 mmol) and anhydrous Et_3_N (0.25 mL, 1.80 mmol, 1.5 equiv.). The corresponding mixture was stirred overnight at rt, under Ar and in the dark. Then, the crude reaction was concentrated to dryness, and the residue was purified by column chromatography (CH_2_Cl_2_→80:1 CH_2_Cl_2_‒MeOH) to give 45 as an orange oil. Yield: 279 mg, 70%; R_F_ 0.60 (EtOAc); ^1^H-NMR (300 MHz, CDCl_3_) δ 6.74 (d, 1H, J_5′,6′_ = 7.8 Hz, H-5′), 6.66 (brd, 1H, J_2′,6′_ = 1.6 Hz, H-2′), 6.61 (dd, 1H, H-6′), 5.92 (s, 2H, OCH_2_O), 5.62 (brs, 1H, NH), 3.46 (q, 2H, J_H,H_ = 6.9 Hz, CH_2_N), 3.09 (t, 2H, J_H,H_ = 6.8 Hz, CH_2_Ar), 2.72 (t, 2H, J_H,H_ = 6.9 Hz, CH_2_CO), 2.31 (brt, 2H, J = 6.7 Hz, CH_2_), 2.20 (quint, 2H, CH_2_CH_2_CH_2_) ppm; ^13^C-NMR (75.5 MHz, CDCl_3_) δ 171.2 (CO), 148.0, 146.4 (C-3′, C-4′), 132.5 (C-1′), 121.7 (C-6′), 109.1, 108.5 (C-5′, C-2′), 101.9 (SeCN), 101.1 (OCH_2_O), 40.8 (CH_2_N), 35.4 (ArCH_2_), 34.8 (CH_2_CO), 29.1 (CH_2_Se), 26.5 (CH_2_CH_2_CH_2_) ppm; HRESI‒MS m/z calcd for C_14_H_16_N_2_NaO_3_Se [M+Na]^+^: 363.0218, found: 363.0218. 

#### 4.2.12. 4,4’-Diselenobis{*N*-[2′’-(3′’’,4′’’-Methylenedioxyphenyl)ethyl]butanamide} (**46**)

To a solution of selenocyanate **45** (128.8 mg, 0.38 mmol) in EtOH (2 mL) was added NaBH_4_ (14.4 mg, 0.38 mmol). The corresponding mixture was stirred at rt overnight; then it was concentrated to dryness and the residue was partitioned between EtOAc (25 mL) and sat. aq. NH_4_Cl (20 mL). The aqueous layer was further washed with EtOAc (3x20 mL); the combined organic fractions were dried over MgSO_4_, filtrated and the filtrate was concentrated to dryness. The residue was purified by column chromatography (1:1→1:3 cyclohexane‒EtOAc) to give 46 as a white solid. Yield: 75.4 mg, 63%; R_F_ 0.38 (EtOAc); ^1^H-NMR (300 MHz, CDCl_3_) δ 6.73 (d, 1H, J_5′’’,6′’’_ = 7.9 Hz, H-5′’’), 6.66 (brd, 1H, J_2′’’,6′’’_ = 1.5 Hz, H-2′’’), 6.61 (dd, 1H, H-6′’’), 5.92 (s, 2H, OCH_2_O), 5.86 (brs, 1H, NH), 3.44 (q, 2H, J_H,H_ = 6.9 Hz, CH_2_N), 2.87 (t, 2H, J_H,H_ = 7.1 Hz, ArCH_2_), 2.72 (t, 2H, J_H,H_ = 6.9 Hz, CH_2_CO), 2.24 (t, 2H, J_H,H_ = 6.9 Hz, CH_2_Se), 2.03 (quint, 2H, CH_2_CH_2_CH_2_) ppm; ^13^C-NMR (75.5 MHz, CDCl_3_) δ 172.2 (CO), 147.9, 146.3 (C-3′’’, C-4′’’), 132.7 (C-1′’’), 121.6 (C-6′’’), 109.1, 108.5 (C-2′’’, C-5′’’), 101.0 (OCH_2_O), 40.9 (CH_2_N), 35.9 (ArCH_2_), 35.5 (CH_2_CO), 29.0 (CH_2_CH_2_CH_2_), 26.7 (CH_2_Se) ppm; HRESI‒MS m/z calcd for C_26_H_33_N_2_O_6_Se_2_ [M+H]^+^: 629.0664, found: 629.0662. 

## 5. Conclusions

We have successfully accomplished the conjugation of phenolic residues with organochalcogen motifs in the search for new potent and selective antiproliferative agents.

A large variety of compounds have been accessed, modifying the nature of the phenolic substituents (either free hydroxyl groups, or the methylenedioxyphenyl moiety), the kind of chalcogen atom (sulfur, selenium), the nature and length of the linker, and also the nature of the chalcogen-containing functionality (disulfide, diselenide, selenide, selenocyanato, benzoselenazolone). Such diverse structural modifications have allowed us to carry out structure-activity relationships searching for the best profile.

Unprotected derivatives, including those with moderate antiproliferative activity, might exert valuable antioxidant activities and act as chemopreventive agents.

Regarding antiproliferative properties, diselenide **40**, bearing a methylenedioxyphenyl residue, and an ethylene tether between both pharmacophoric units, was found to be the lead compound in terms of potency (GI_50_ = 0.88‒2.0 µM) and selectivity (selectivity index: 14‒32). Diselenide **46**, incorporating an amide-type linker was found to be the most potent derivative within the series (GI_50_ = 0.12‒0.27 µM), but with diminished selectivity (selectivity index: 4‒8).

Remarkably, selenoderivatives tested herein were not found to be substrates for the P-gp efflux pump, so presumably such compounds will not undergo chemoresistance via this transport pump for excreting xenobiotics.

## Figures and Tables

**Table 1:** Antiradical activity for organochalcogen derivatives.

Compound	EC_50_ (µM)
**4**	>175
**8**	12.3 ± 0.5
**9**	4.2 ± 0.1
**10**	15.3 ± 0.1
**15**	7.8 ± 0.2
**16**	9.3 ± 1.3
**18**	23.2 ± 0.6
**20**	10.8 ± 0.4
**26**	7.8 ± 0.2
**39**	>250
**46**	>250
**HT**	13.4 ± 0.6
**Dopamine**	11.4 ± 1.2

**Table 2:** H_2_O_2_ scavenging properties ^1^.

Compound	% Scavenging
**4**	--- ^2^
**8**	26.5 ± 2.1
**9**	79.8 ± 0.8
**10**	11.8 ± 0.5
**15**	40.0 ± 3.1
**16**	52.9 ± 3.4
**18**	19.0 ± 0.8
**20**	25.8 ± 1.0
**26**	53.8 ± 2.3
**39**	---
**46**	---
**Dopamine**	46.1 ± 4.6

^1^ At 50 µM; ^2^ No scavenging properties were observed at the tested concentration.

**Table 3:** Selected GI_50_ values (µM) for the antiproliferative activity of organochalcogen derivatives.

Compound	A549 (Lung)	HBL-100 (Breast)	HeLa (Cervix)	T-47D (Breast)	WiDr (Colon)	BJ-hTert (Fibroblasts)	S.I.
**15**	5.4 ± 1.1	29 ± 6	3.6 ± 1.1	39 ± 5	31 ± 7	---^1^	---
**16**	3.7 ± 0.6	2.4 ± 0.6	2.3 ± 0.4	5.8 ± 0.9	3.9 ± 0.6	3.5 ± 0.6	0.6‒1.5
**18**	33 ± 5	34 ± 1	23 ± 1	71 ± 5	85 ± 21	---	---
**20**	28 ± 8	37 ± 2	19 ± 8	35 ± 1	41 ± 10	---	---
**35**	3.1 ± 0.7	3.5 ± 1.4	3.5 ± 0.8	3.4 ± 0.2	2.0 ± 0.1	8.7 ± 3.2	2.5‒4.4
**36**	1.8 ± 0.7	2.1 ± 0.2	1.2 ± 0.2	1.1 ± 0.3	1.3 ± 0.6	14 ± 2	6.7‒13
**37**	Not soluble						
**38**	19 ± 2	14 ± 3	5.2 ± 1.0	4.7 ± 1.2	14 ± 2	---	---
**39**	1.2 ± 0.3	2.9 ± 0.6	2.4 ± 1.4	2.9 ± 0.4	1.6 ± 0.3	3.9 ± 1.0	1.3‒3.3
**40**	1.6 ± 0.5	2.0 ± 0.9	0.95 ± 0.07	0.88 ± 0.17	1.2 ± 0.1	28 ± 5	14‒32
**41**	32 ± 8	93 ± 10	31 ± 8	29 ± 5	29 ± 8	---	
**45**	0.41 ± 0.03	1.6 ± 0.3	0.36 ± 0.09	0.40 ± 0.04	0.41 ± 0.03	1.8 ± 0.7	1.1‒5.0
**46**	0.27 ± 0.02	0.25 ± 0.02	0.12 ± 0.01	0.19 ± 0.04	0.25 ± 0.06	1.0 ± 0.1	3.7‒8.3
**Ebselen**	25 ± 9	13 ± 3	26 ± 8	90 ± 14	>100	---	---
**CDDP**	4.9 ± 0.2	1.9 ± 0.2	2.0 ± 0.3	15 ± 2	26 ± 6	14 ± 2	0.5‒7.4

^1^ Not tested.

**Table 4:** Antiproliferative activity (GI_50_) of organochalcogen derivatives in SW1573 and SW1573/P-gp cell lines ^1^.

	w/o Verapamil			w Verapamil		
	SW1573	SW1573/PGP	Rf	SW153	SW1573/PGP	Rf
**15**	3.3 ± 0.4	5.0 ± 0.1	1.5	5.0 ± 0.7	5.3 ± 0.3	1.1
**16**	2.7 ± 0.3	5.1 ± 0.6	1.9	2.3 ± 0.3	5.1 ± 0.4	2.2
**35**	2.3 ± 0.5	7.1 ± 0.6	3.1	3.1 ± 1.0	7.0 ± 0.6	2.3
**36**	4.2 ± 0.7	6.8 ± 0.4	1.6	3.1 ± 0.4	5.3 ± 0.6	1.7
**38**	27 ± 2	29 ± 0.2	1.1	15 ± 3	25 ± 2	1.6
**39**	3.0 ± 0.5	18 ± 0.1	6.0	2.5 ± 0.1	5.1 ± 0.4	2.0
**40**	1.9 ± 0.1	3.9 ± 0.2	2.0	1.8 ± 0.2	4.5 ± 1.0	2.5
**45**	1.1 ± 0.2	3.3 ± 0.6	3.1	1.1 ± 0.1	2.5 ± 0.1	2.3
PTX	1.5 ± 0.5	196 ± 53	128	1.6 ± 0.2	4.2 ± 0.9	2.6
VB	0.9 ± 0.3	2051 ± 682	2388	0.8 ± 0.2	1.0 ± 0.5	1.3

^1^ Paclitaxel (PTX) and vinblastine (VB) were used as reference drugs. GI_50_ values are given in μM for organochalcogen derivatives and in nM for PTX and VB. Standard deviation was calculated from at least two independent experiments. Rf represents the ratio between GI_50_ values in SW1573/PGP and SW1573 cells.

## Supplementary Materials

The following are available online at https://www.mdpi.com/1424-8247/13/11/358/s1. Table S1: GI_50_ values (µM) for the antiproliferative activity of organoselenium derivatives. ^1^H- and ^13^C-NMR spectra of compounds **4**, **8**, **9**, **10**, **15**, **16**, **18**‒**20**, **26**, **35**‒**42**, **45**, **46**.
