# Supplementary material for: Masked Phenolic-Selenium Conjugates: Potent and Selective Antiproliferative Agents Overcoming P-gp Resistance
Source: Pharmaceuticals (Basel). 2020 Oct 31;13(11):358. doi: 10.3390/ph13110358 (PMC7692337; doi:10.3390/ph13110358)
Supplement: Supplementary file 1 [file pharmaceuticals-13-00358-s001.pdf]

Article

Masked Phenolic-Selenium Conjugates: Potent and Selective Antiproliferative Agents Overcoming P-gp Resistance

Paloma Begines [1,†], Lucía Sevilla-Horrillo [2,†], Adrián Puerta [3], Rebecca Puckett [2], Samuel Bayort [1], Irene Lagunes [3], Inés Maya [1], José M. Padrón [3,*], Óscar López [1,*] and José G. Fernández-Bolaños [1,*]

1 Departamento de Química Orgánica, Facultad de Química, Universidad de Sevilla, Apartado 1203, E-41071 Seville, Spain; pbegines@us.es (P.B.); samuel_etegarrobo@hotmail.com (S.B.); imaya@us.es (I.M.)
2 Escuela Politécnica Superior, Universidad de Sevilla, Virgen de África 7, E-41011 Seville, Spain; lucia_sh_93@msn.com (L.S.-H.); rebpucper@hotmail.com (R.P.)
3 BioLab, Instituto Universitario de Bio-Orgánica "Antonio González" (IUBO-AG), Universidad de La Laguna, c/ Astrofísico Francisco Sánchez 2, E-38206 La Laguna, Spain; apuertaa@ull.es (A.P.); roslagunes@uv.mx (I.L.)
* Correspondence: jmpadron@ull.es (J.M.P.); osc-lopez@us.es (Ó.L.); bolanos@us.es (J.G.F.-B.); Tel.: +34-922-316-502 (J.M.P.) ext. 6126; +34-954-559-997 (Ó.L.); +34-954-550-996 (J.G.F.-B.)
† These authors contributed equally to this work.

Table S1……………………………………………………………………………..S2

^{1}H-, ^{13}C-NMR and ^{77}Se spectra……………………..……………………………S3-S66

Table S1. GI_{50} values (μM) for the antiproliferative activity of organoselenium derivatives

Compound	A549 (Lung)	HBL-100 (Breast)	HeLa (Cervix)	SW1573 (Lung)	T-47D (Breast)	WiDr (Colon)	BJ-hTert (Human fibroblasts)
**2**	>100	>100	>100	>100	>100	>100	---[1]
**6**	>100	>100	>100	>100	>100	>100	---
**7**	>100	>100	>100	>100	>100	>100	---
**4**	>100	>100	>100	>100	>100	>100	---
**8**	>100	>100	>100	>100	>100	>100	---
**9**	32±1	31±7	28±2	42±6	45±19	>100	---
**10**	>100	>100	>100	>100	>100	>100	---
**13**	**4.1±0.1**	94±8	18±1	>100	20±8	5.6±0.6	---
**14**	46±14	>100	44±13	>100	50±14	54±6	---
**15**	**5.4±1.1**	29±6	**3.6±1.1**	**5.9±1.3**	39±5	31±7	---
**16**	**3.7±0.6**	**2.4±0.6**	**2.3±0.4**	**3.0±0.2**	**5.8±0.9**	**3.9±0.6**	3.5±0.6
**17**	>100	>100	>100	>100	>100	>100	---
**18**	33±5	34±1	23±1	28±4	71±5	85±21	---
**19**	18±6	93±9	32±10	>100	36±1	28±4	---
**20**	28±8	37±2	19±8	24±8	35±1	41±10	---
**26**	21±7	21±4	21±6	21±12	28±6	30±3	---
**35**	**3.1±0.7**	**3.5±1.4**	**3.5±0.8**	**4.4±0.5**	**3.4±0.2**	**2.0±0.1**	8.7±3.2
**36**	**1.8±0.7**	**2.1±0.2**	**1.2±0.2**	**3.8±0.1**	**1.1±0.3**	**1.3±0.6**	14±2
**37**				Not soluble			
**38**	19±2	14±3	**5.2±1.0**	27±5	**4.7±1.2**	14±2	---
**39**	**1.2±0.3**	**2.9±0.6**	**2.4±1.4**	**3.2±0.9**	**2.9±0.4**	**1.6±0.3**	3.9±1.0
**40**	**1.6±0.5**	**2.0±0.9**	**0.95±0.07**	**2.7±0.3**	**0.88±0.17**	**1.2±0.1**	28±5
**41**	32±8	93±10	31±8	37±15	29±5	29±8	---
**45**	**0.41±0.03**	**1.6±0.3**	**0.36±0.09**	**0.81±0.02**	**0.40±0.04**	**0.41±0.03**	1.8±0.7
**46**	**0.27±0.02**	**0.25±0.02**	**0.12±0.01**	**0.30±0.03**	**0.19±0.04**	**0.25±0.06**	1.0±0.1
**Ebselen**	25±9	13±3	26±8	28±4	90±14	>100	---
**CDDP**	4.9±0.2	1.9±0.2	2.0±0.3	3.4±0.7	15±2	26±6	14±2

[1]Not tested

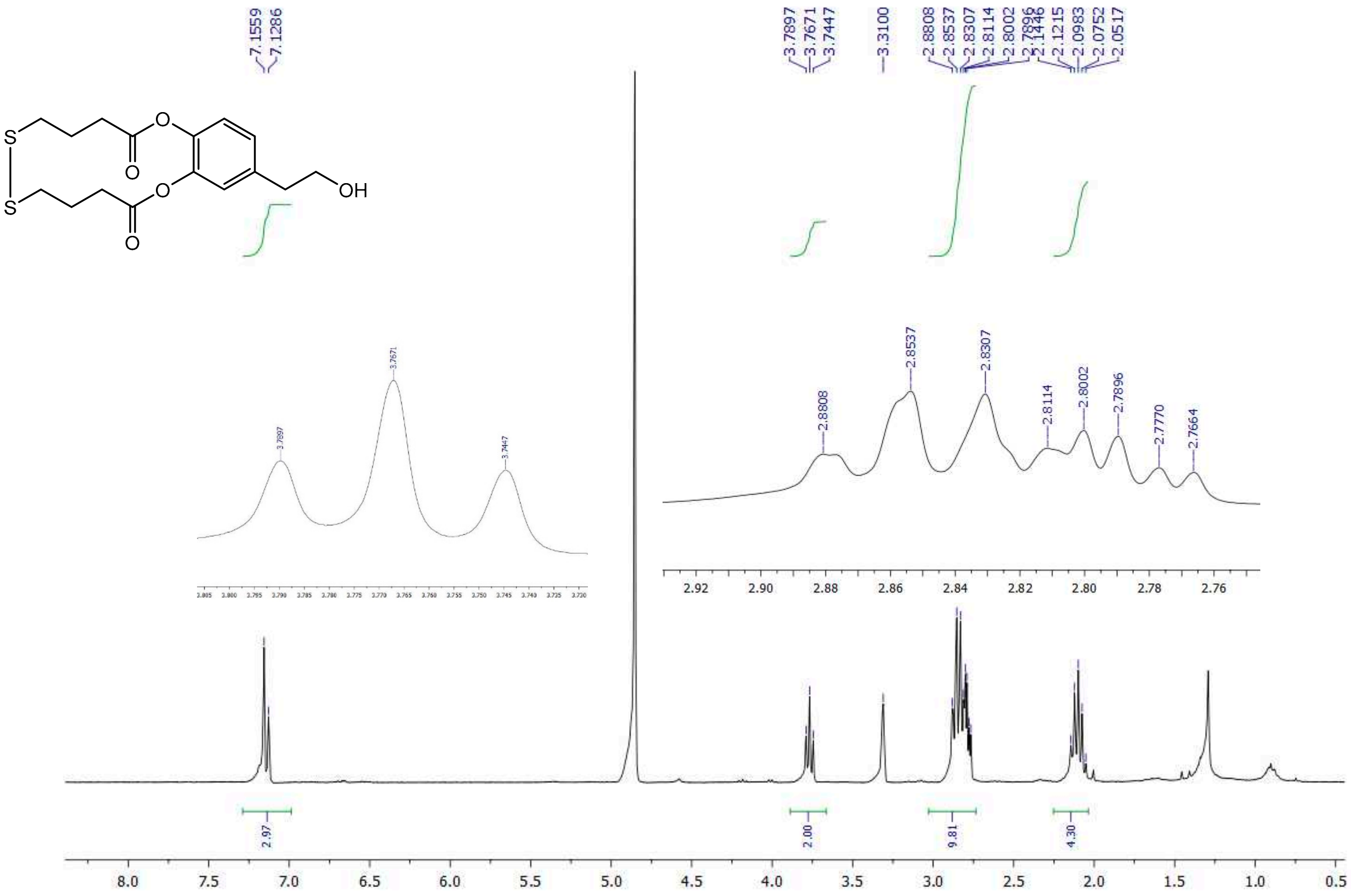

^{1}H-NMR (300 MHz, CD_3OD) of **4**

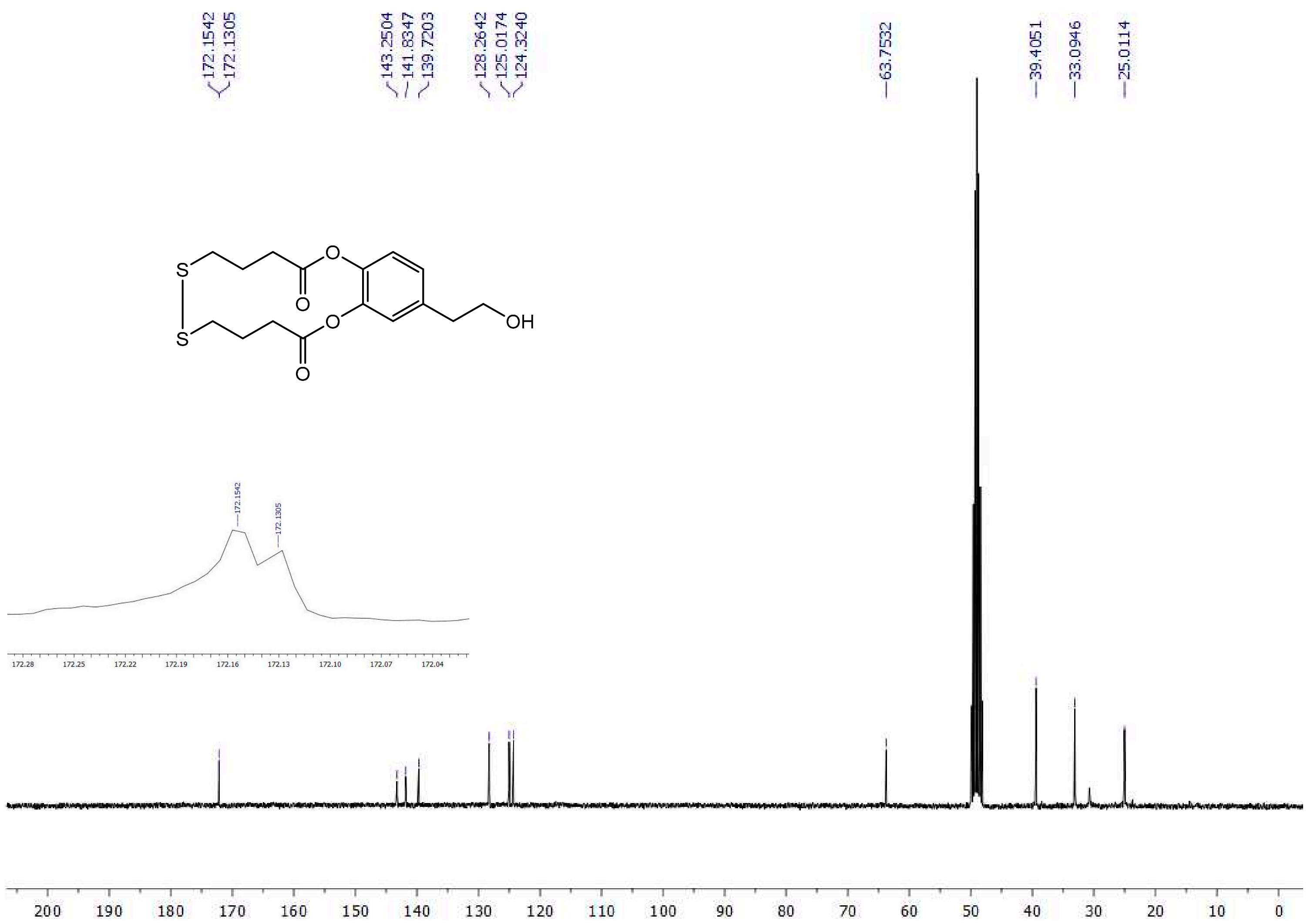

^{13}C- NMR (75.5 MHz, CD_3OD) of **4**

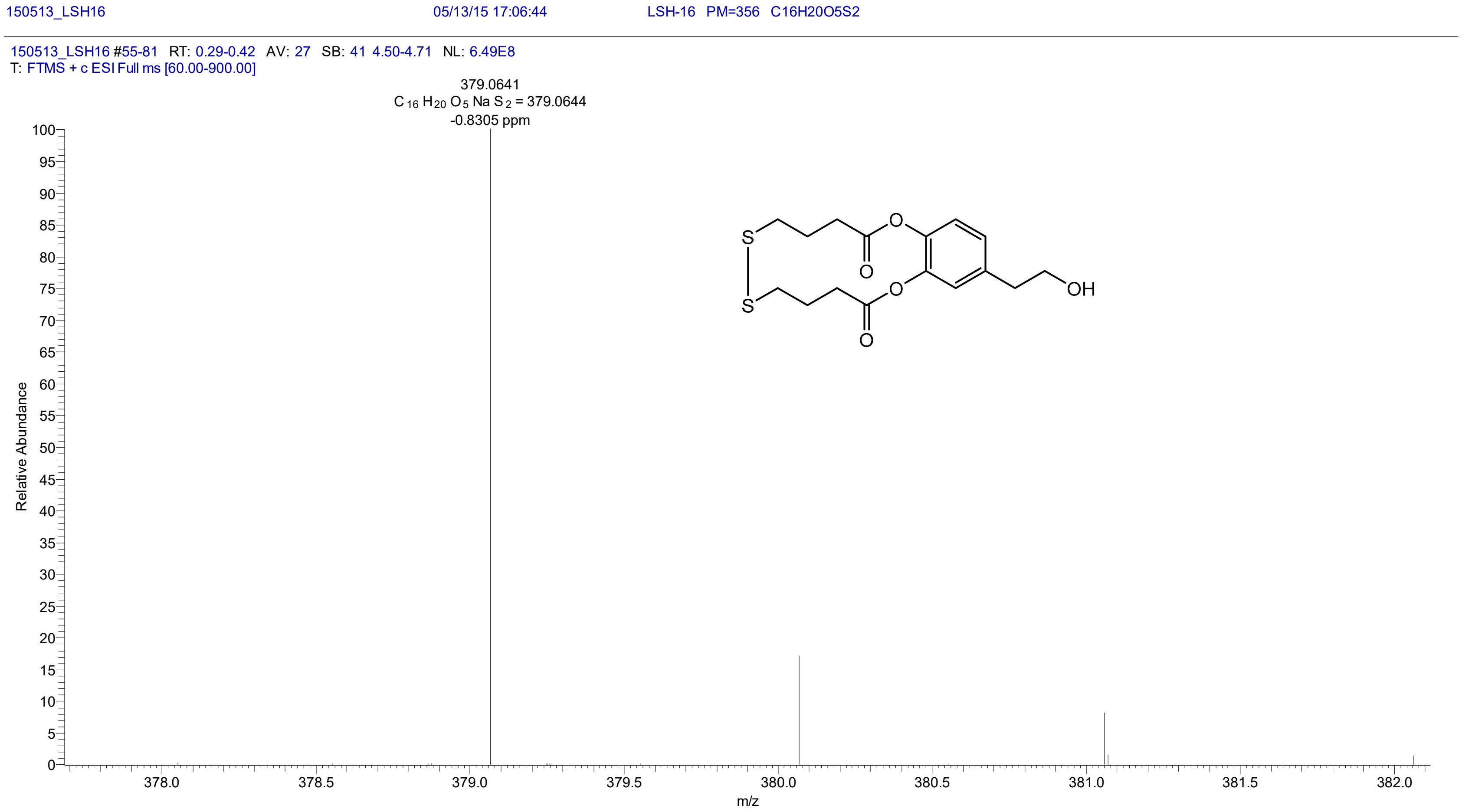

HRESI-MS spectrum of **4**

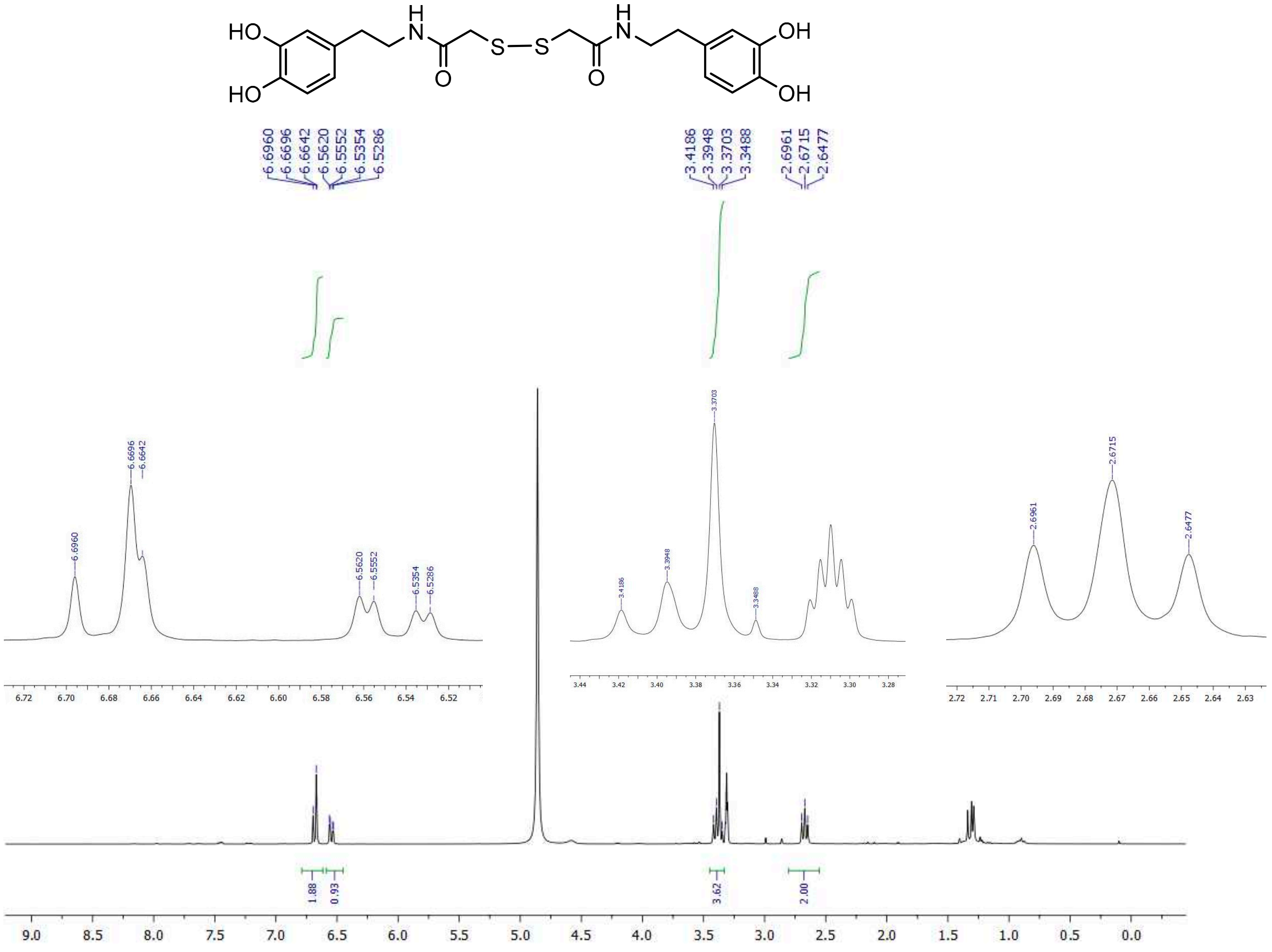

^{1}H- NMR (300 MHz, CD_3OD) of **8**

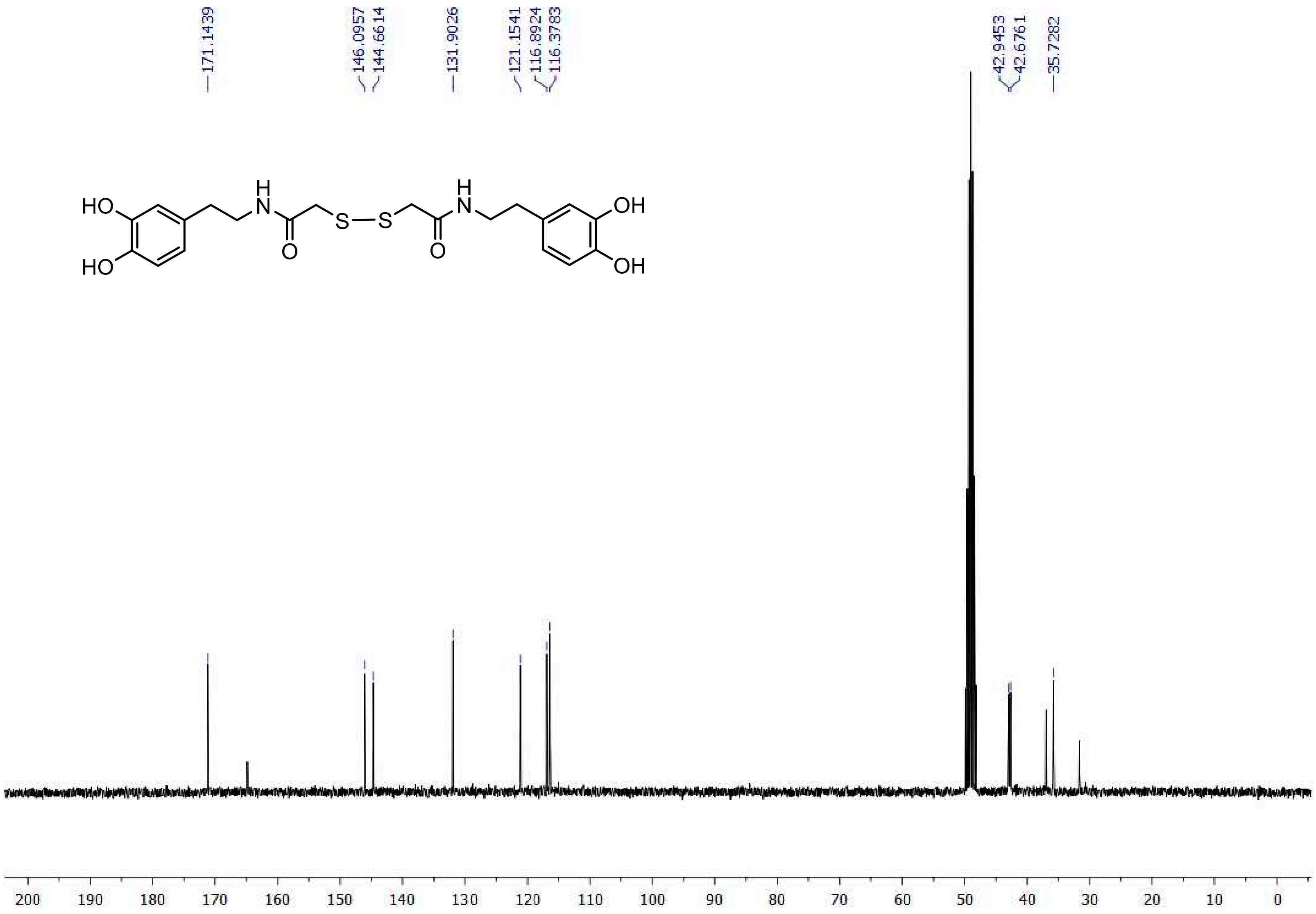

^{13}C- NMR (75.5 MHz, CD_3OD) of **8**

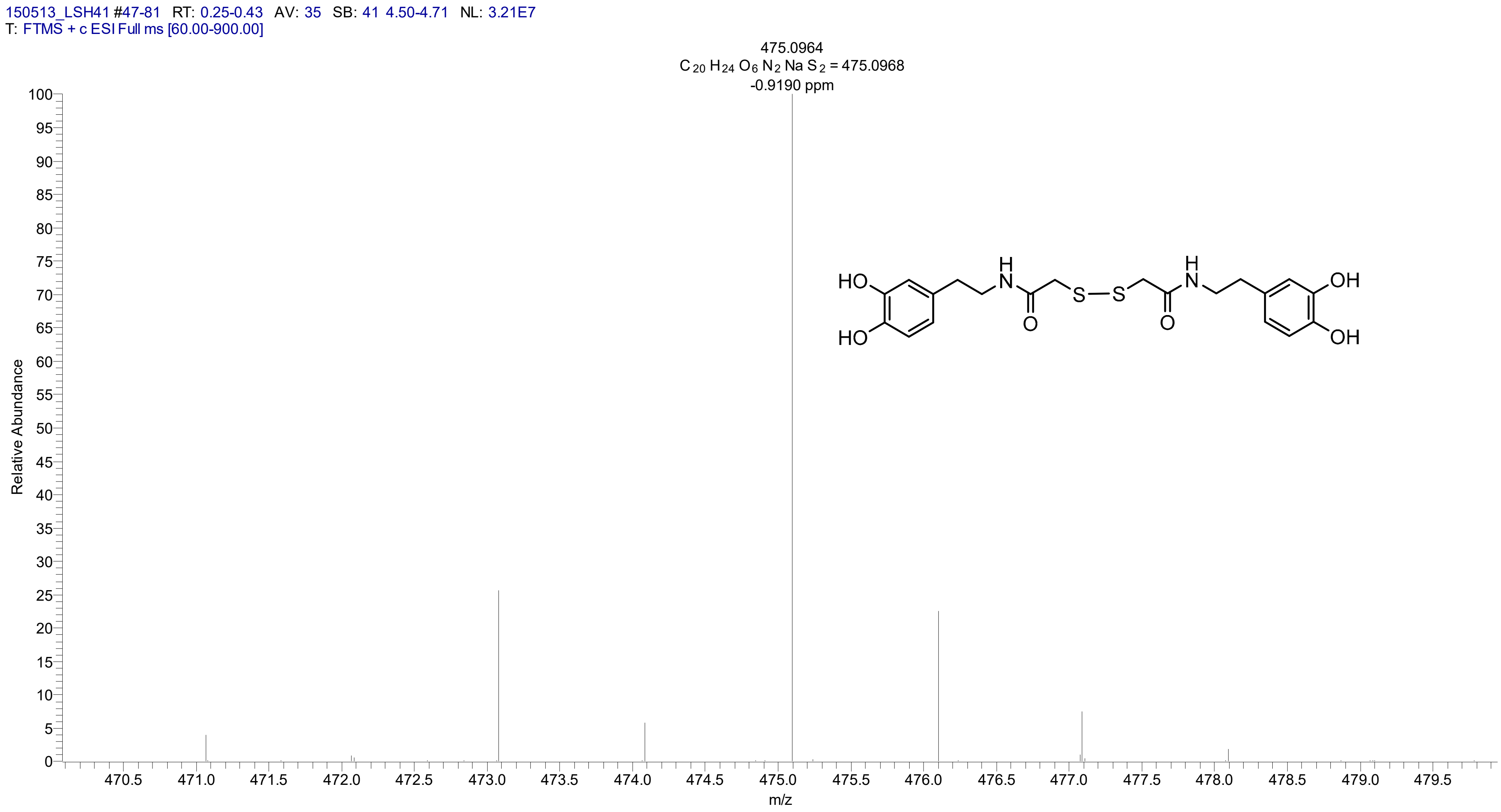

HRESI-MS spectrum of **8**

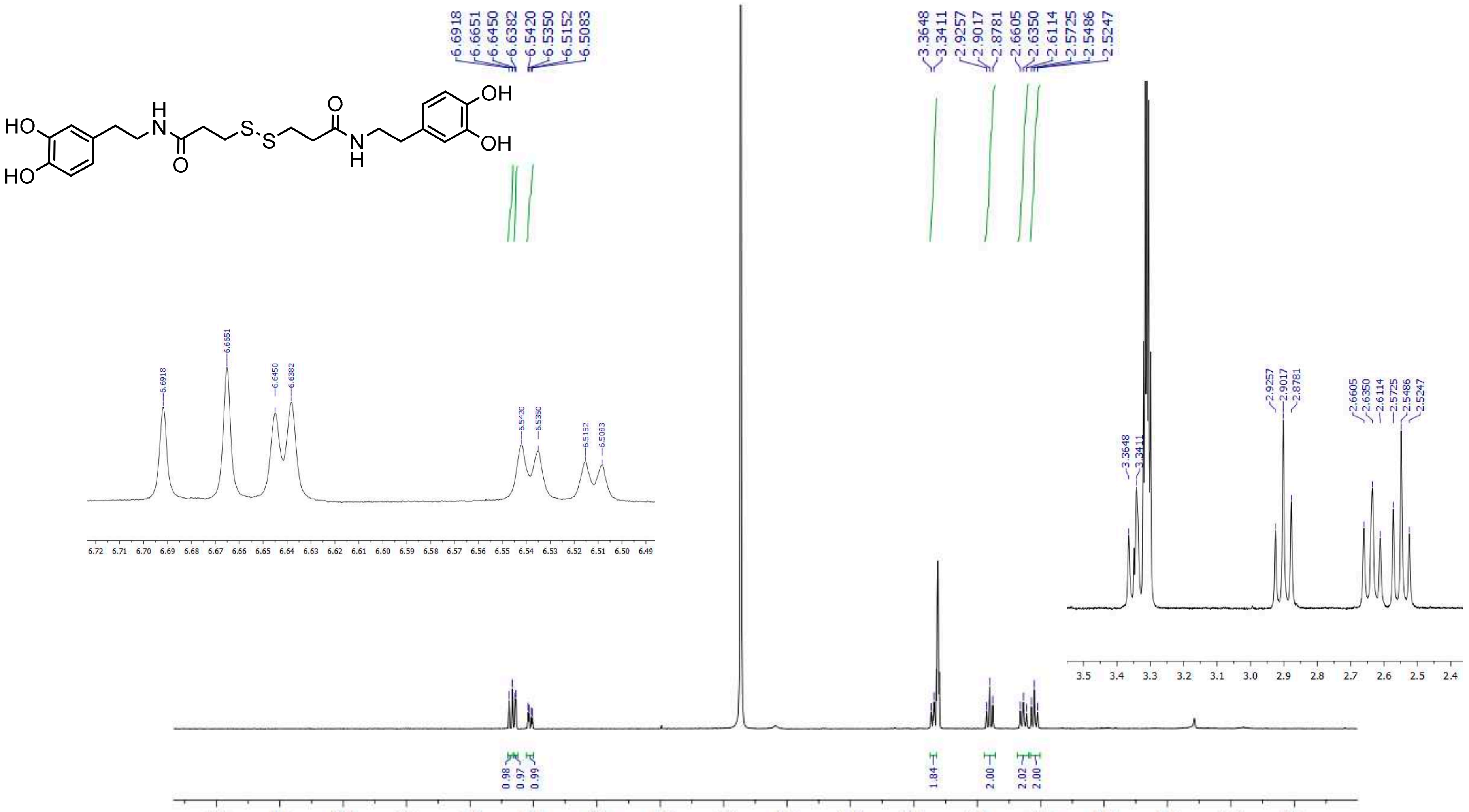

^1H- NMR (500 MHz, CD_3OD) of **9**

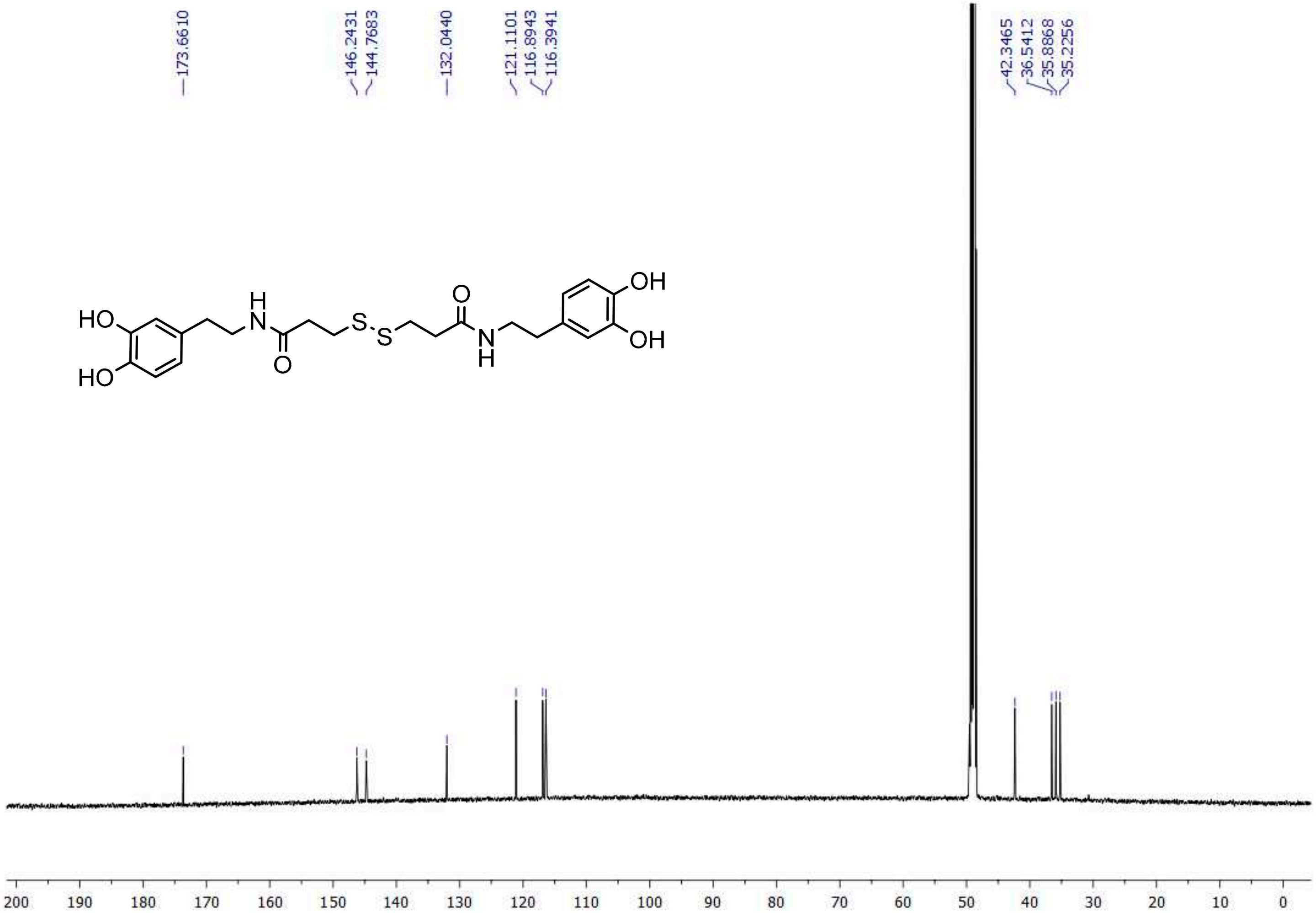

^{13}C- NMR (125.7 MHz, CD_3OD) of **9**

150513_LSH29 05/13/15 16:32:44 LSH-29 PM=480 C22H28N2O6S2

150513_LSH29 #49-74 RT: 0.26-0.39 AV: 26 SB: 40 4.50-4.71 NL: 1.88E7
T: FTMS + c ESI Full ms [60.00-900.00]

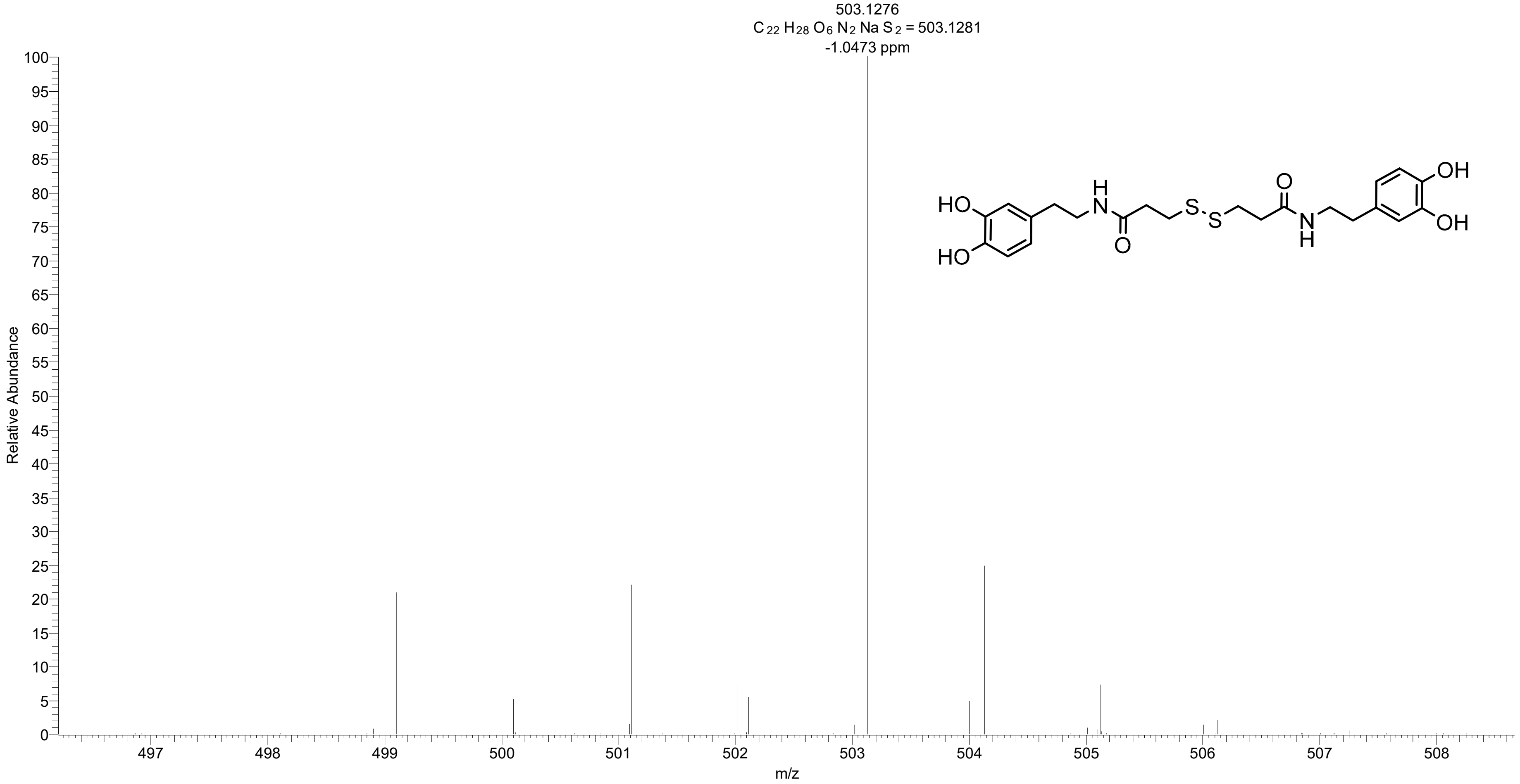

HRESI-MS spectrum of **9**

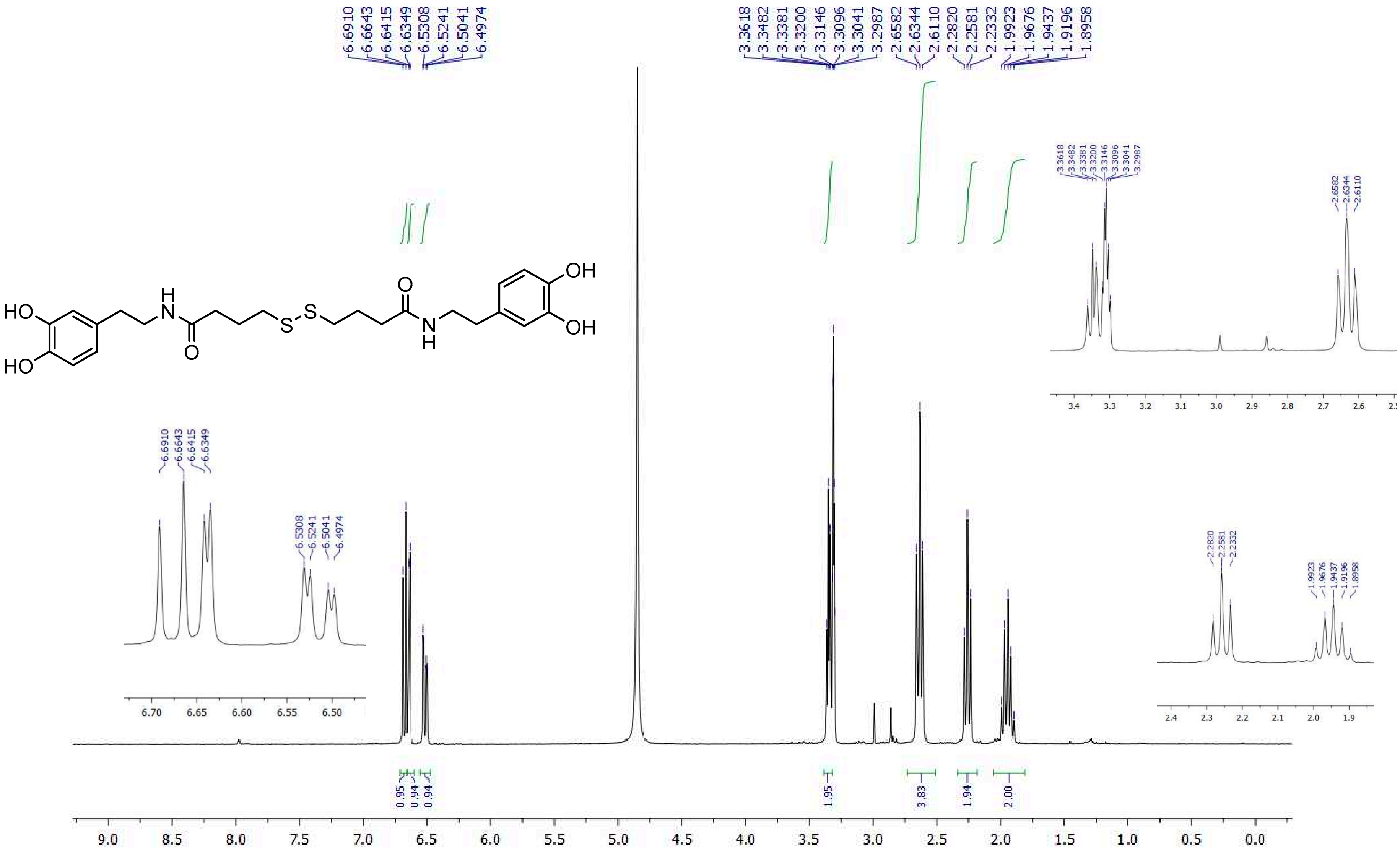

^{1}H- NMR (300 MHz, CD_3OD) of **10**

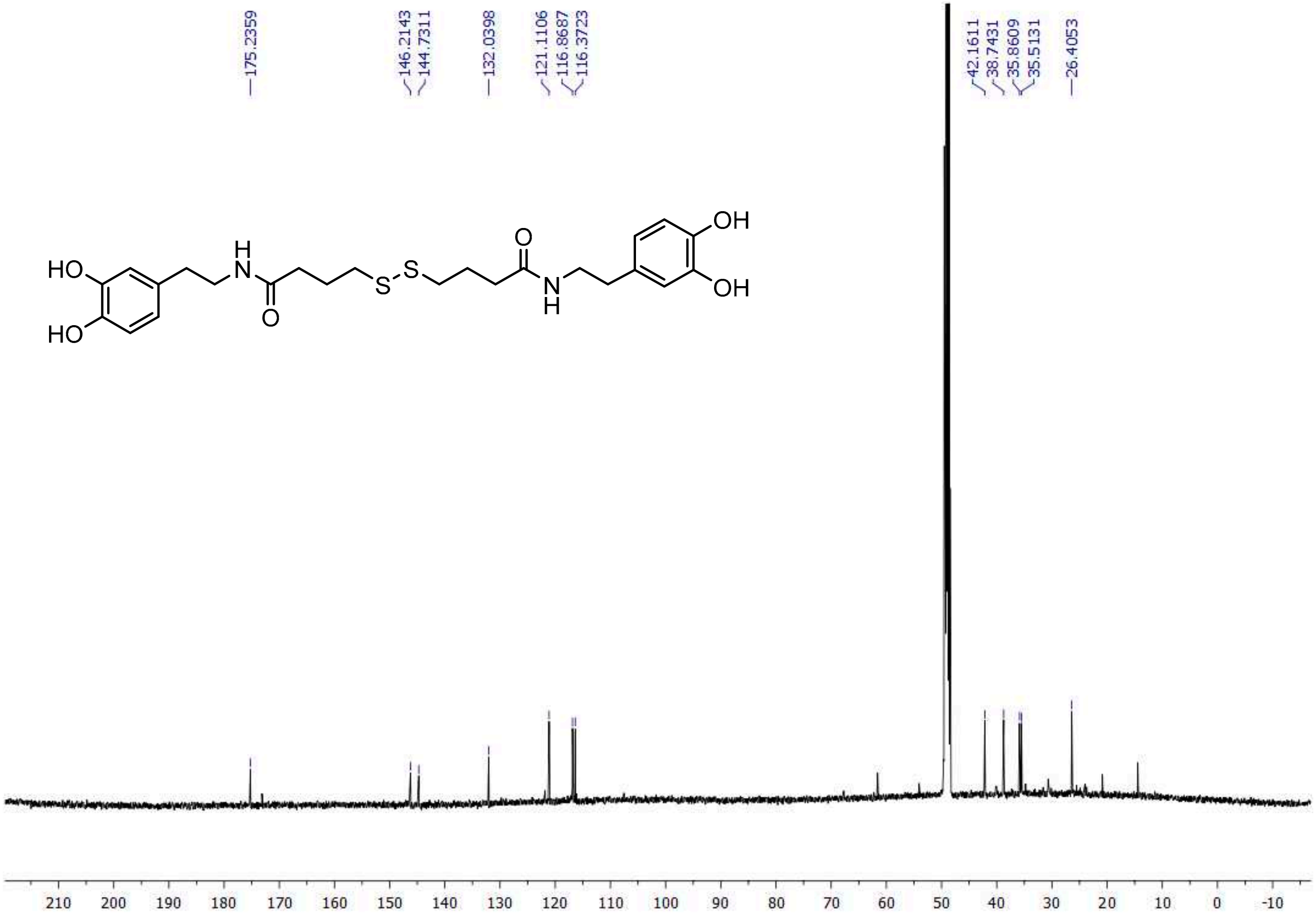

^{13}C- NMR (125.7 MHz, CD_3OD) of **10**

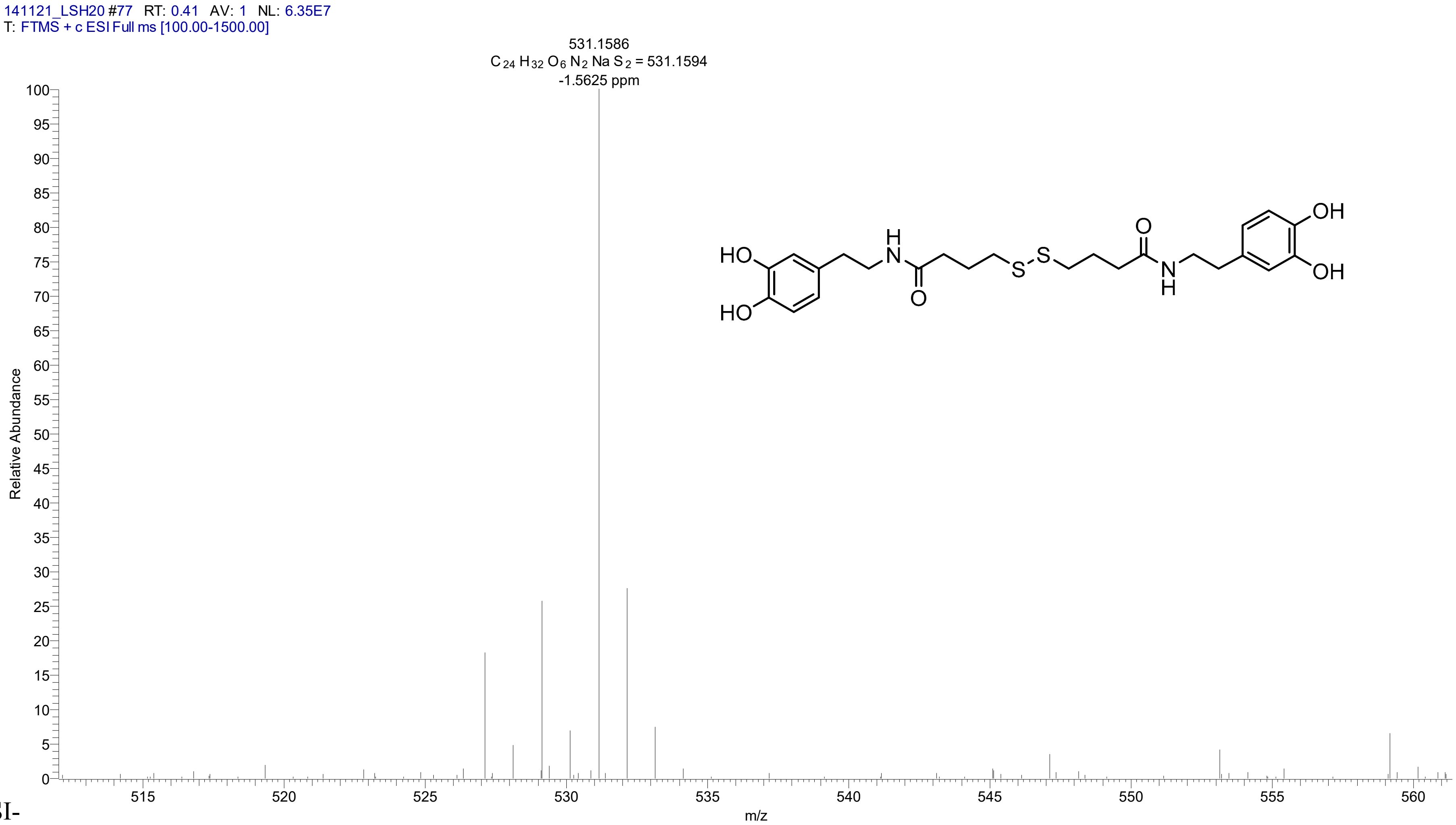

HRESI-MS spectrum of **10**

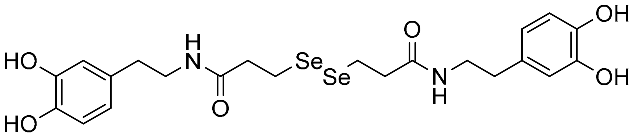

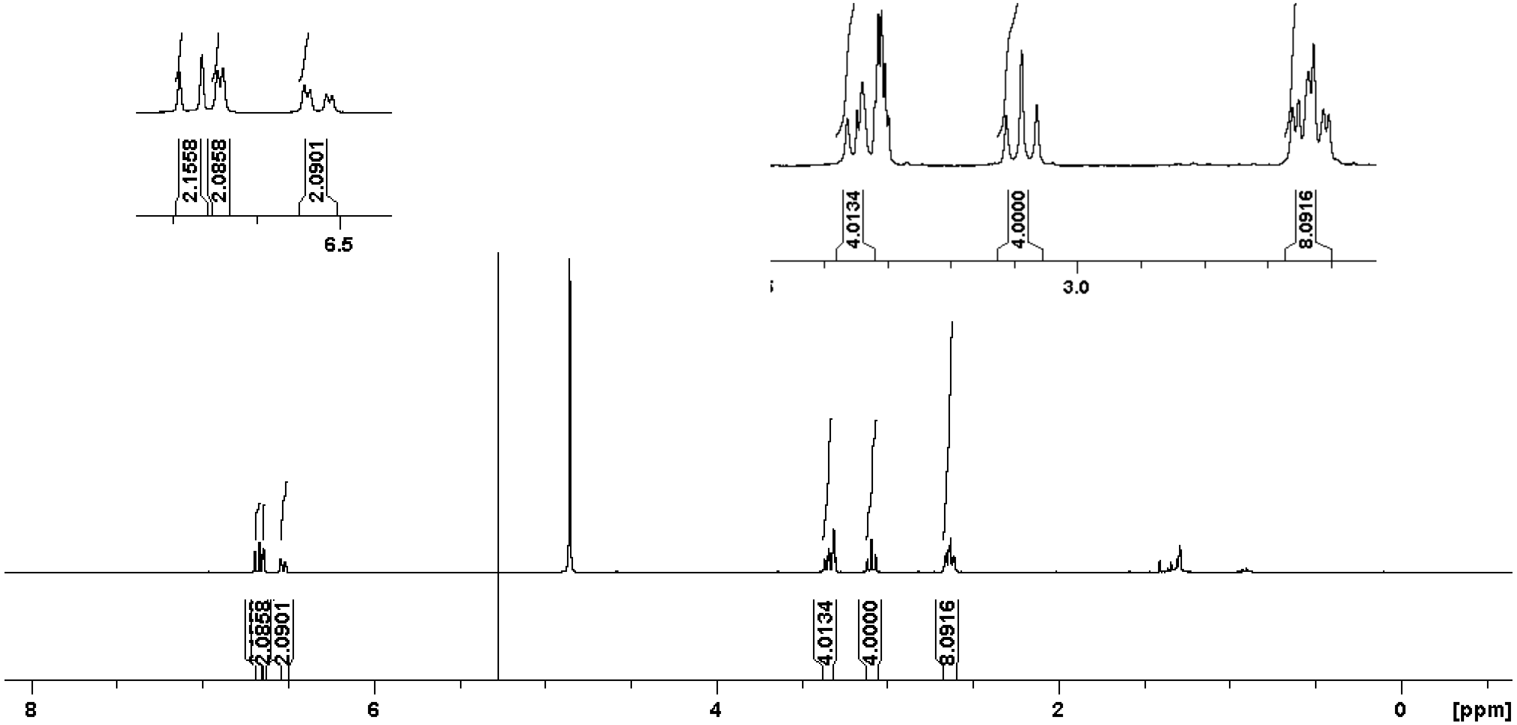

^{1}H- NMR (300 MHz, CD$_3$OD) of **15**

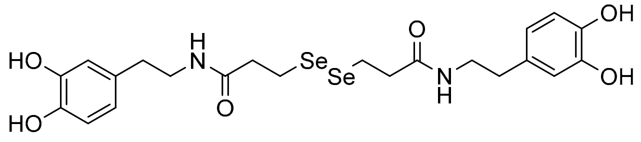

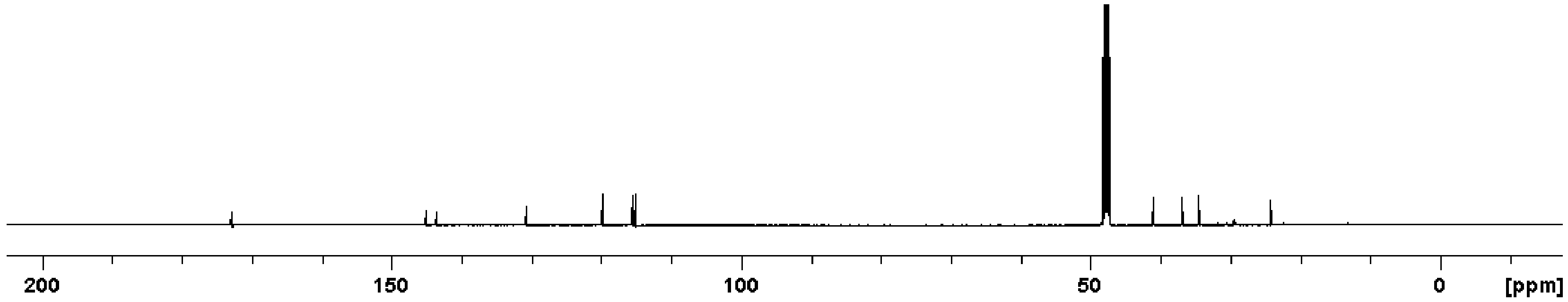

^{13}C- NMR (125.7 MHz, CD_3OD) of **15**

150513_LSH32 05/13/15 16:46:18 LSH-32 PM=576 C22H28N2O6Se2

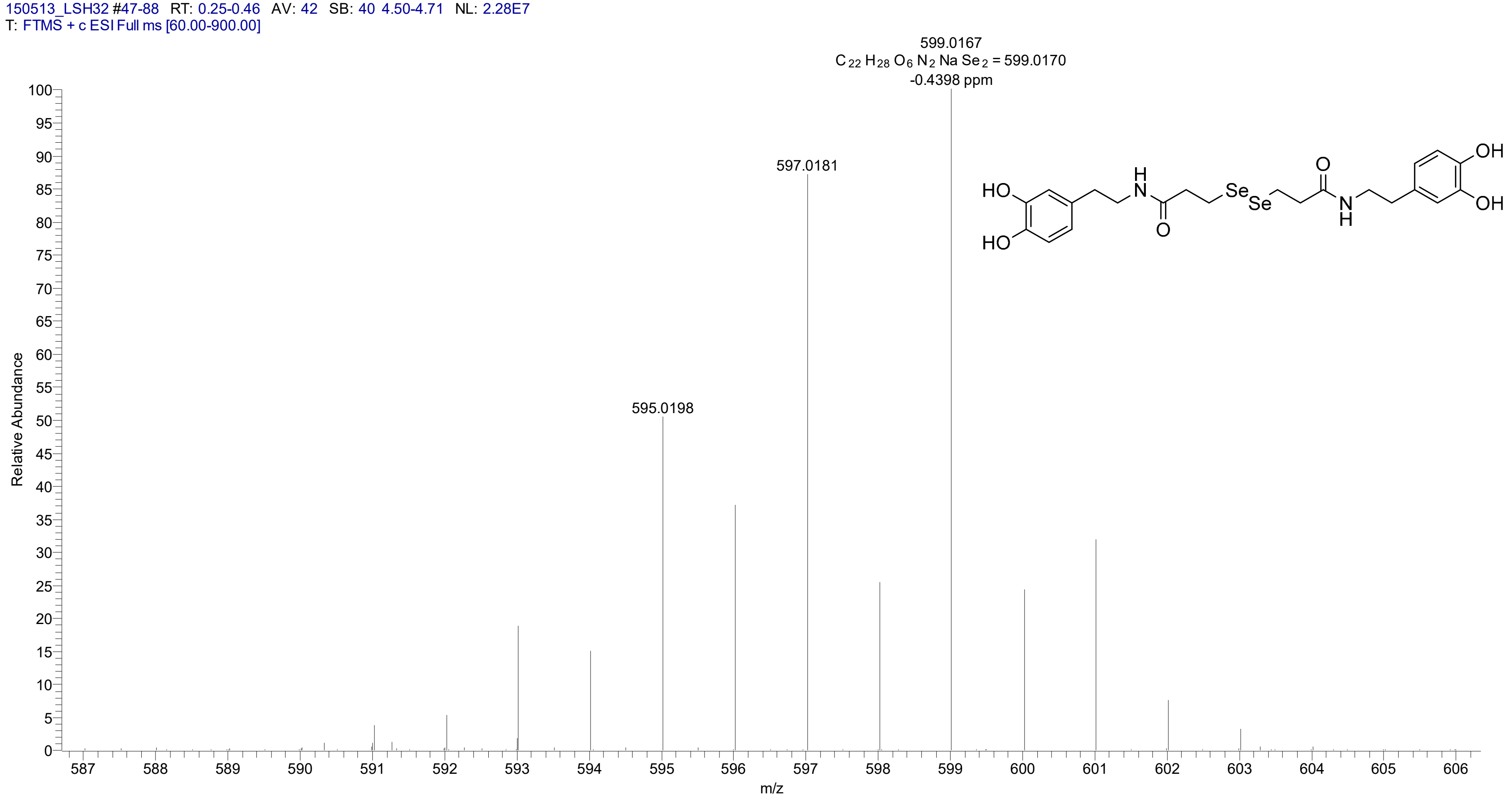

HRESI-MS spectrum of **15**

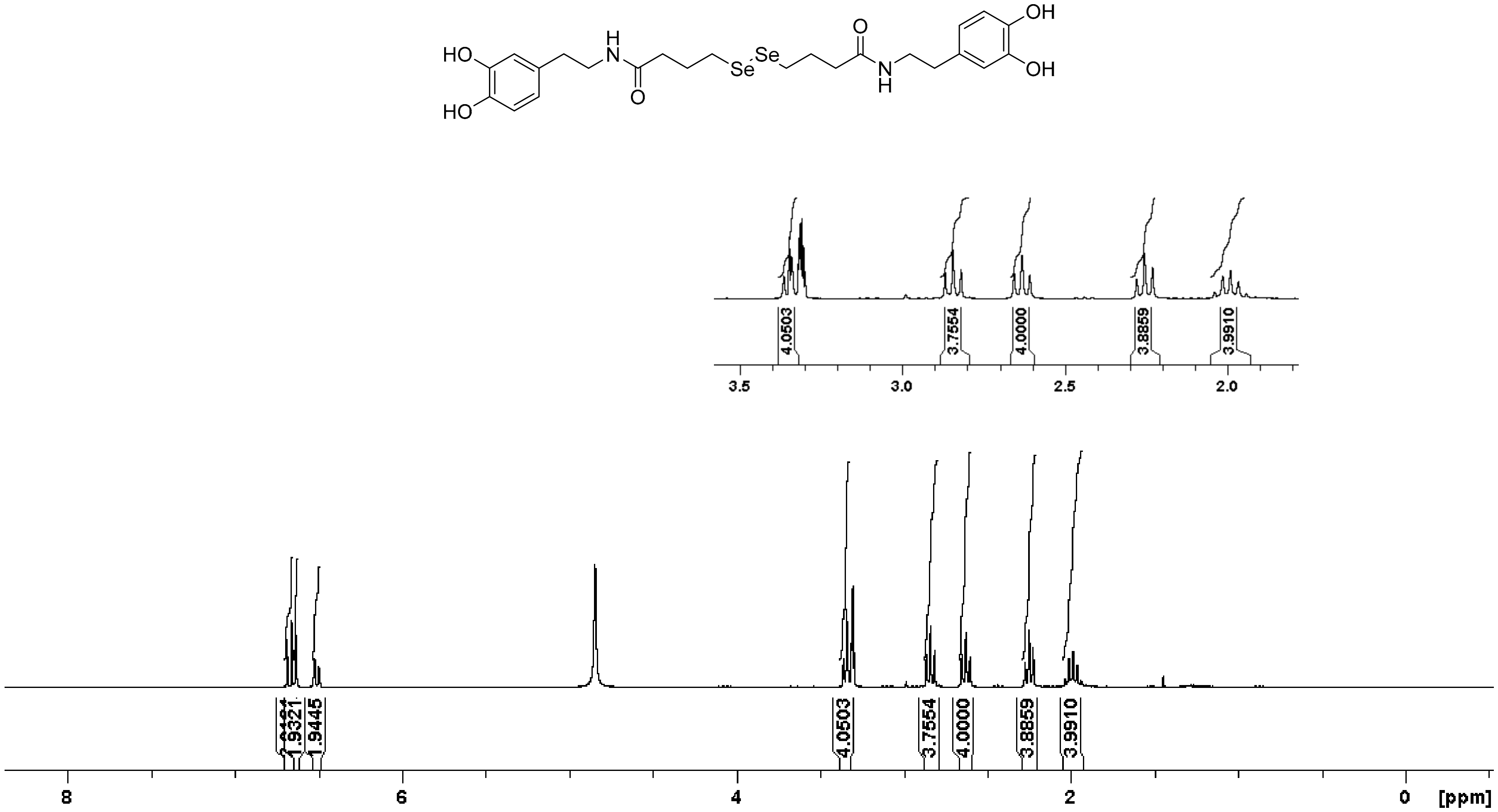

^{1}H- NMR (300 MHz, CD_3OD) of **16**

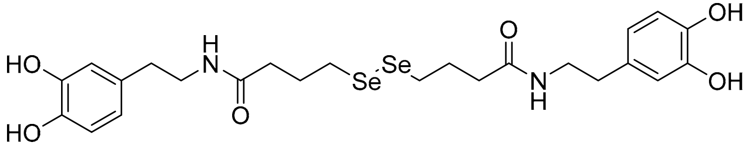

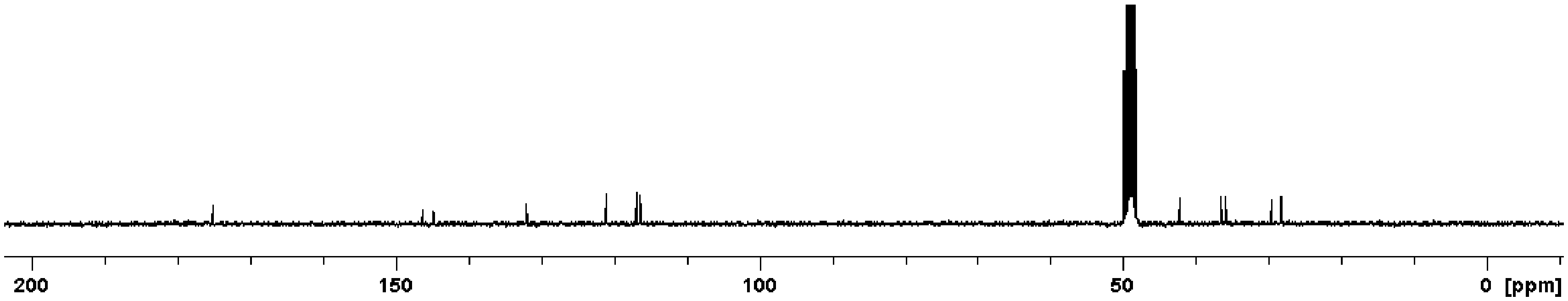

^{13}C- NMR (125.7 MHz, CD_3OD) of **16**

150911_LSH54 09/11/15 13:33:01 LSH-54 PM=604 C24H32N2O6Se2

150911_LSH54 #52-63 RT: 0.27-0.33 AV: 12 NL: 2.68E7
T: FTMS + c ESI Full ms [60.00-900.00]

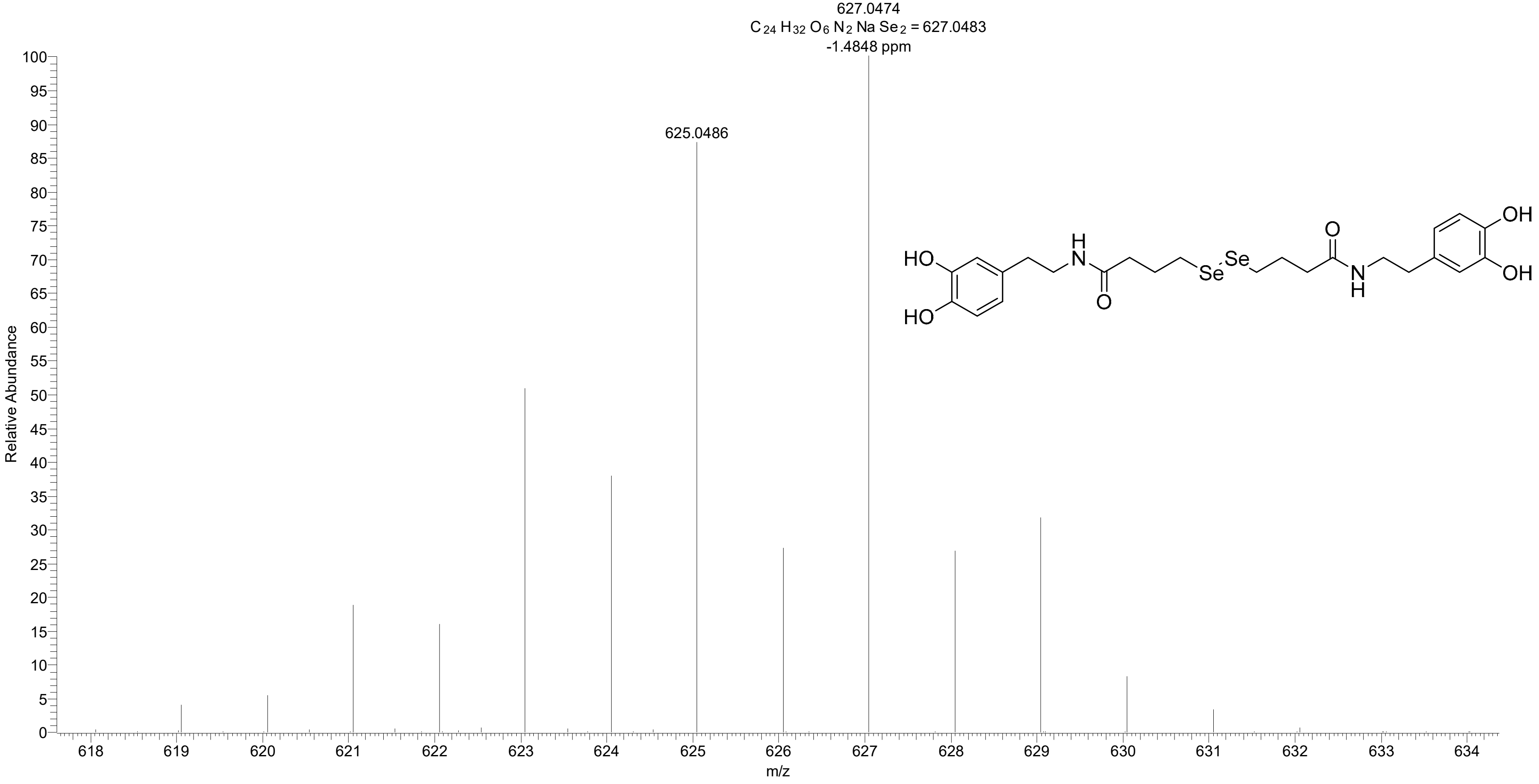

HRESI-MS spectrum of **16**

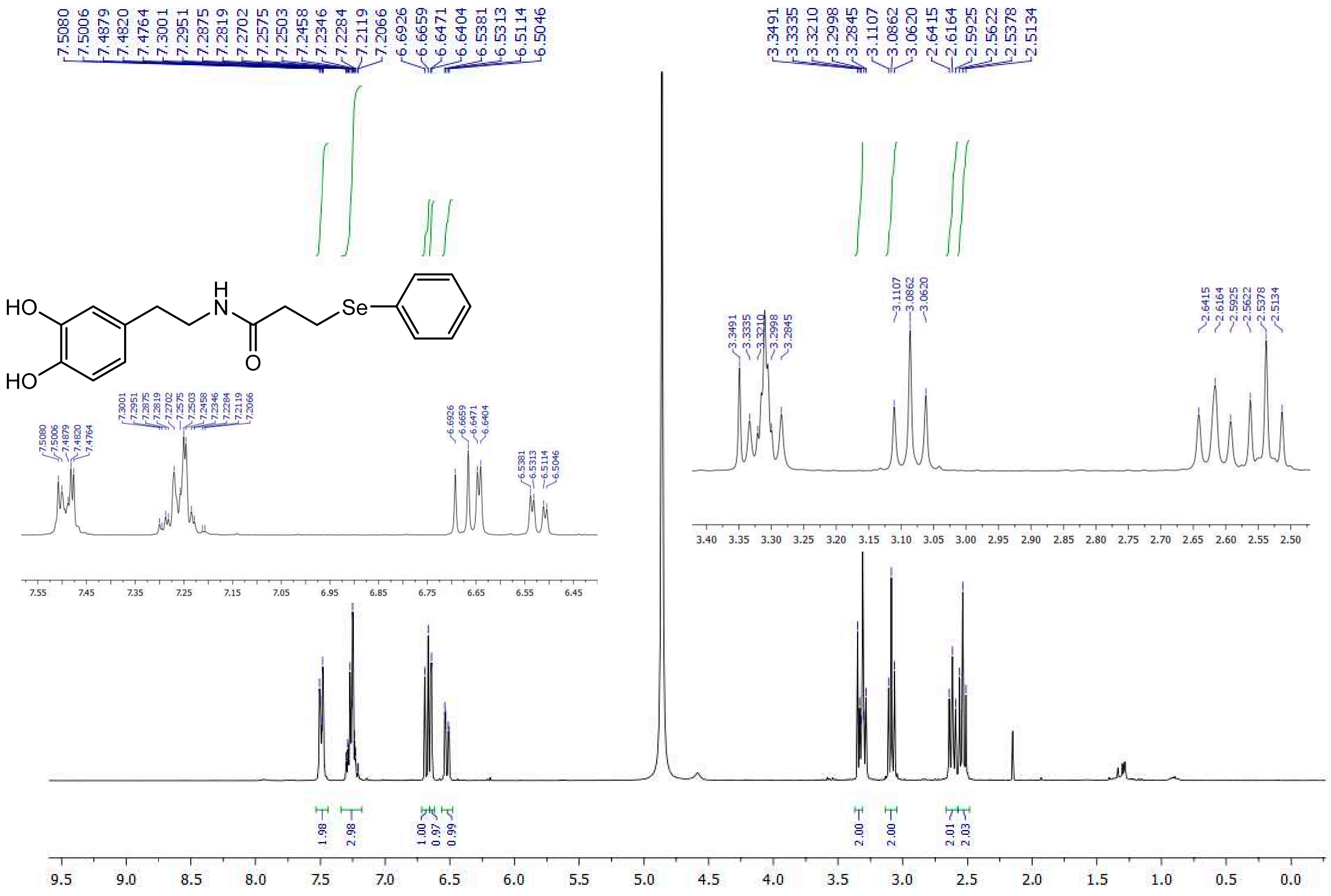

^{1}H- NMR (300 MHz, CD_3OD) of **18**

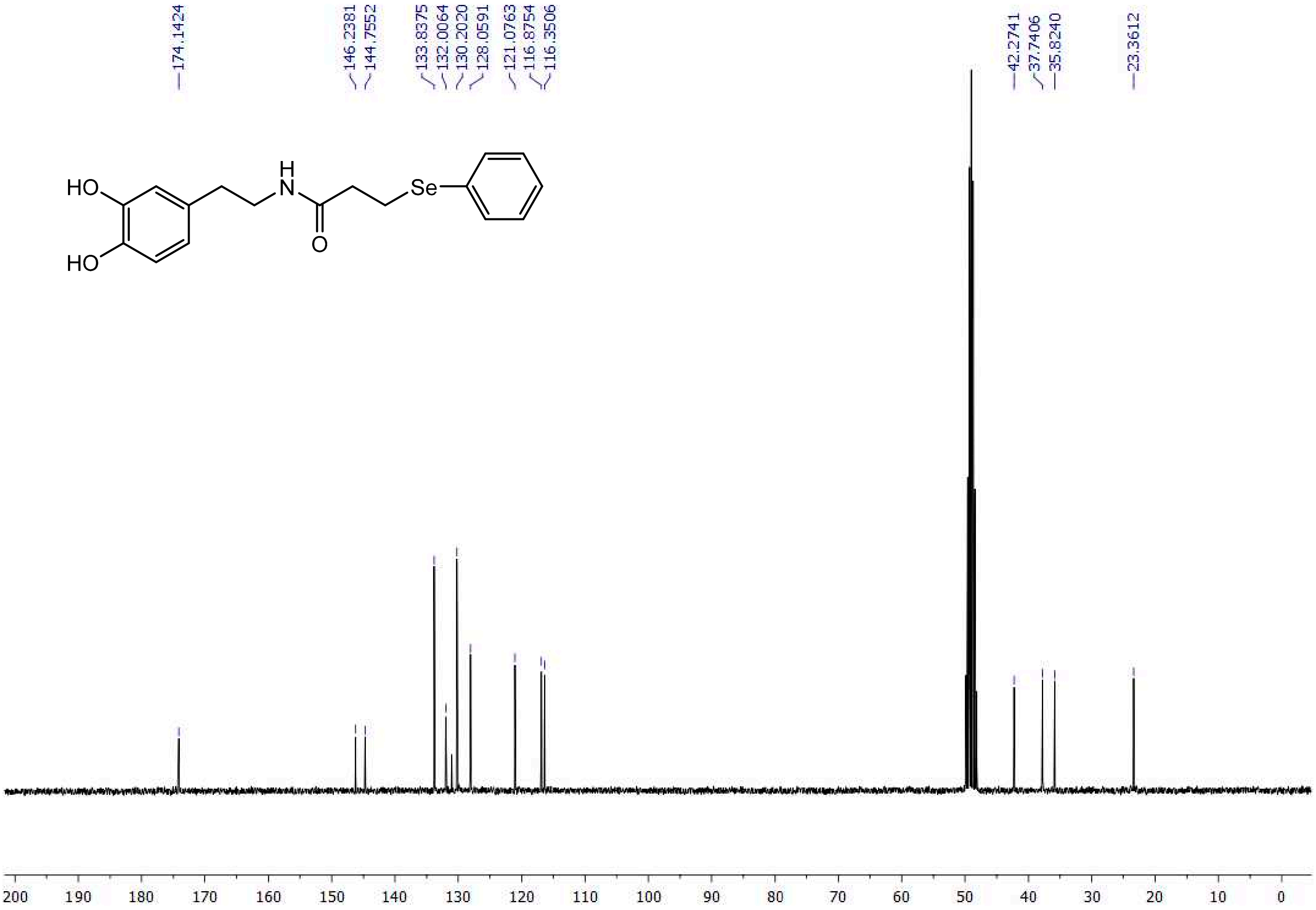

^{13}C- NMR (75.5 MHz, CD_3OD) of **18**

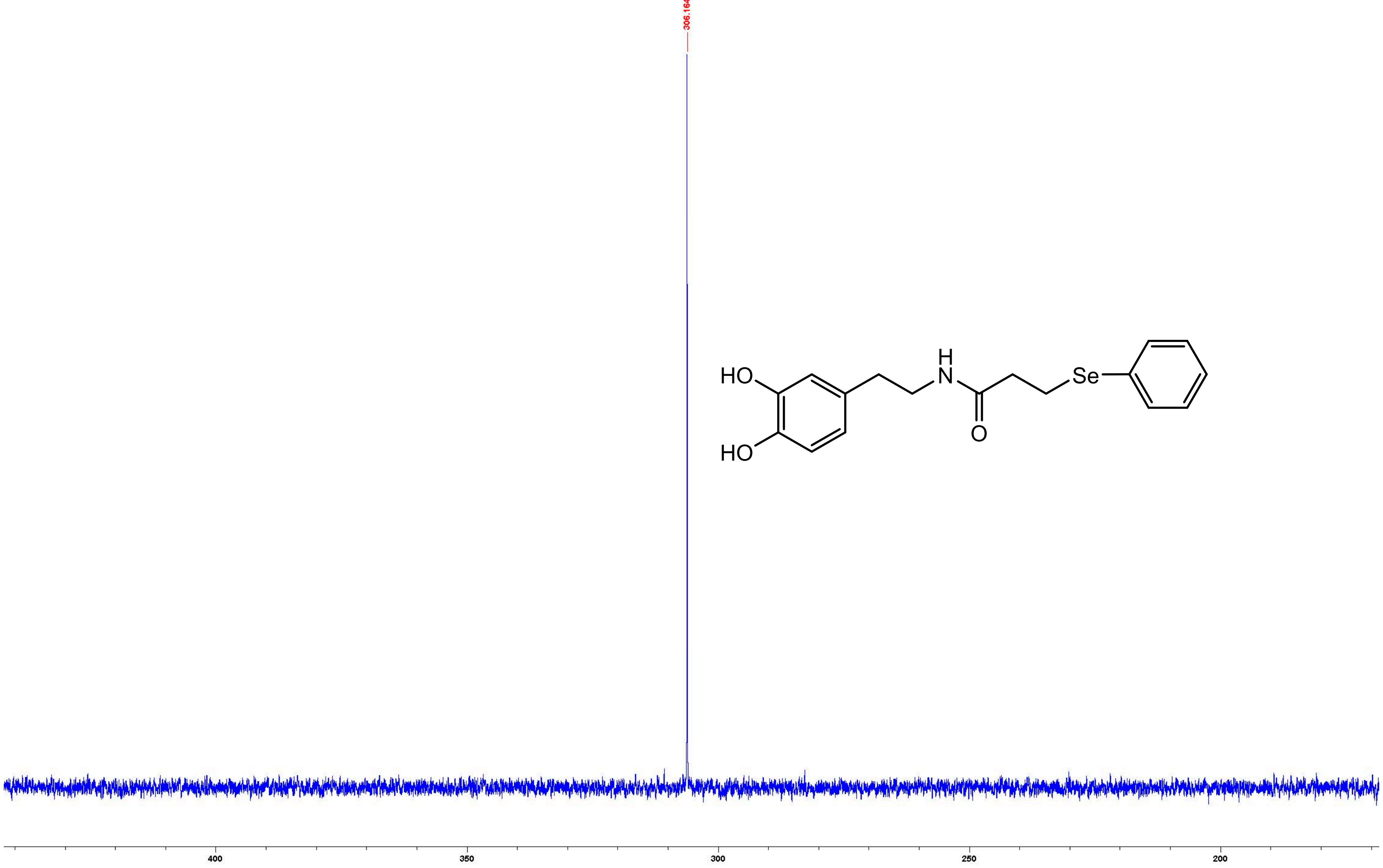

^{77}Se- NMR (95 MHz, CCl_3) of **18**

150513_LSH30 05/13/15 16:39:30 LSH-30 PM365 C17H19NO3Se

150513_LSH30 #42-66 RT: 0.22-0.35 AV: 25 SB: 41 4.50-4.71 NL: 1.56E8
T: FTMS + c ESI Full ms [60.00-900.00]

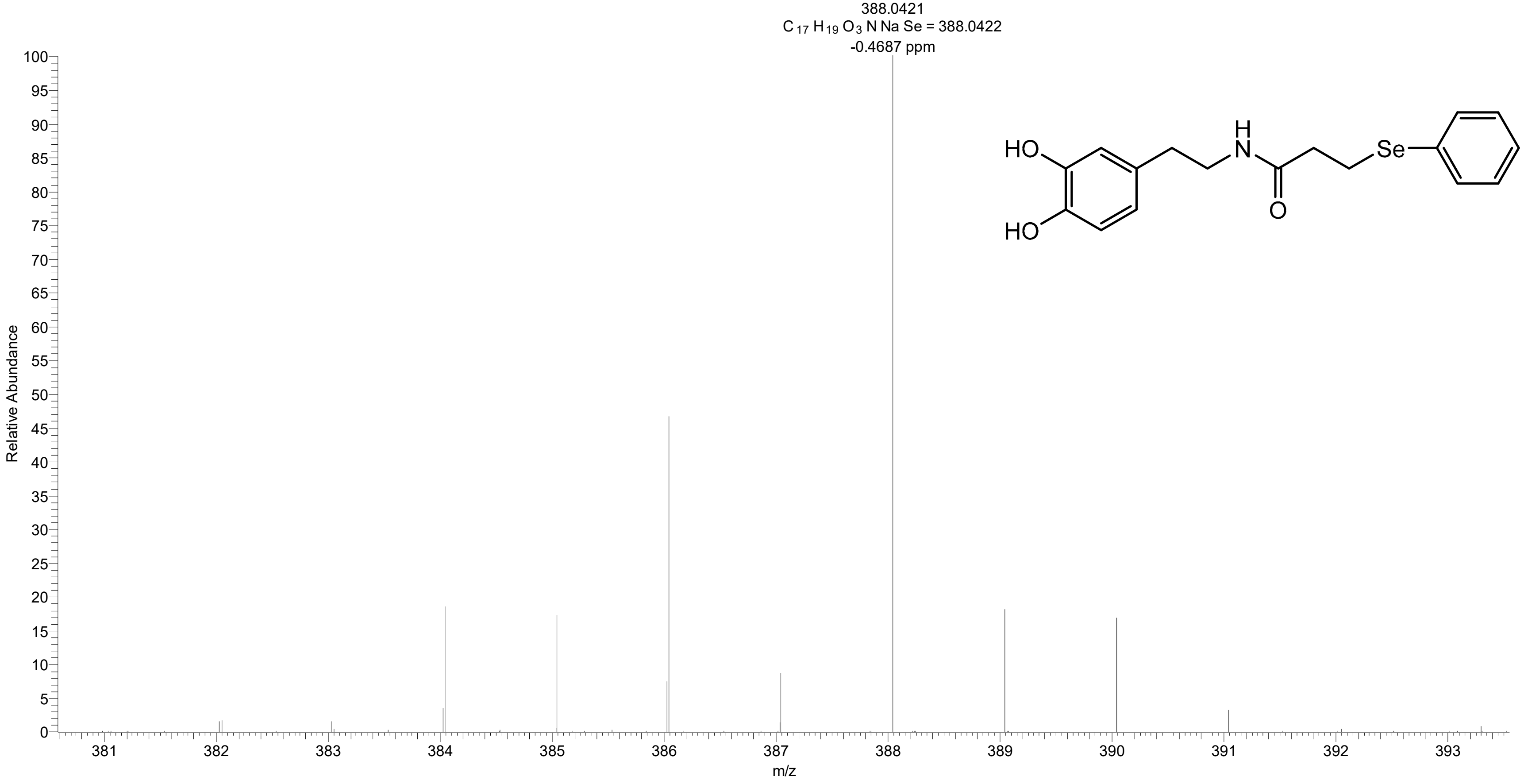

HRESI-MS spectrum of **18**

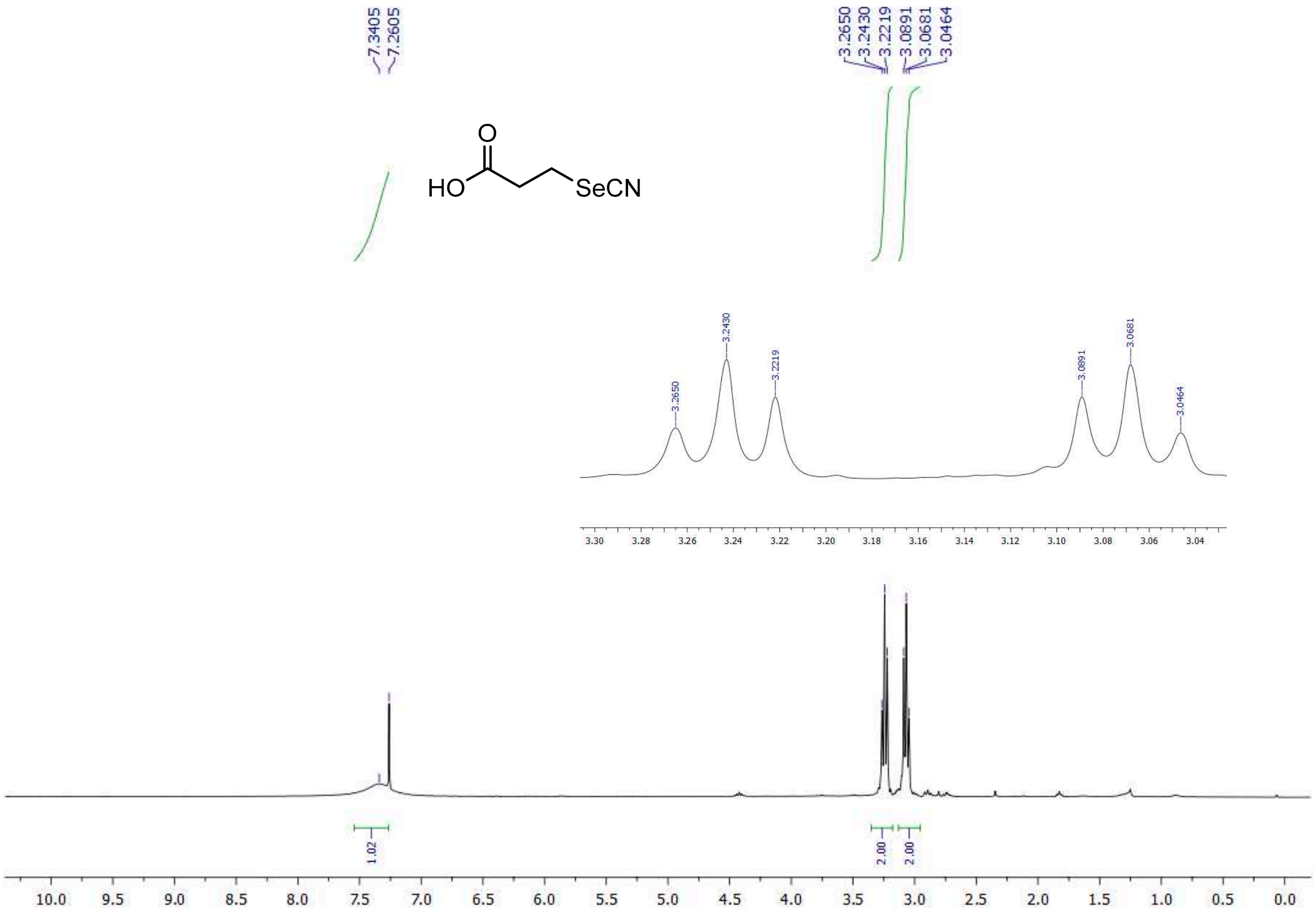

^{1}H- NMR (300 MHz, $CDCl_3$) of **19**

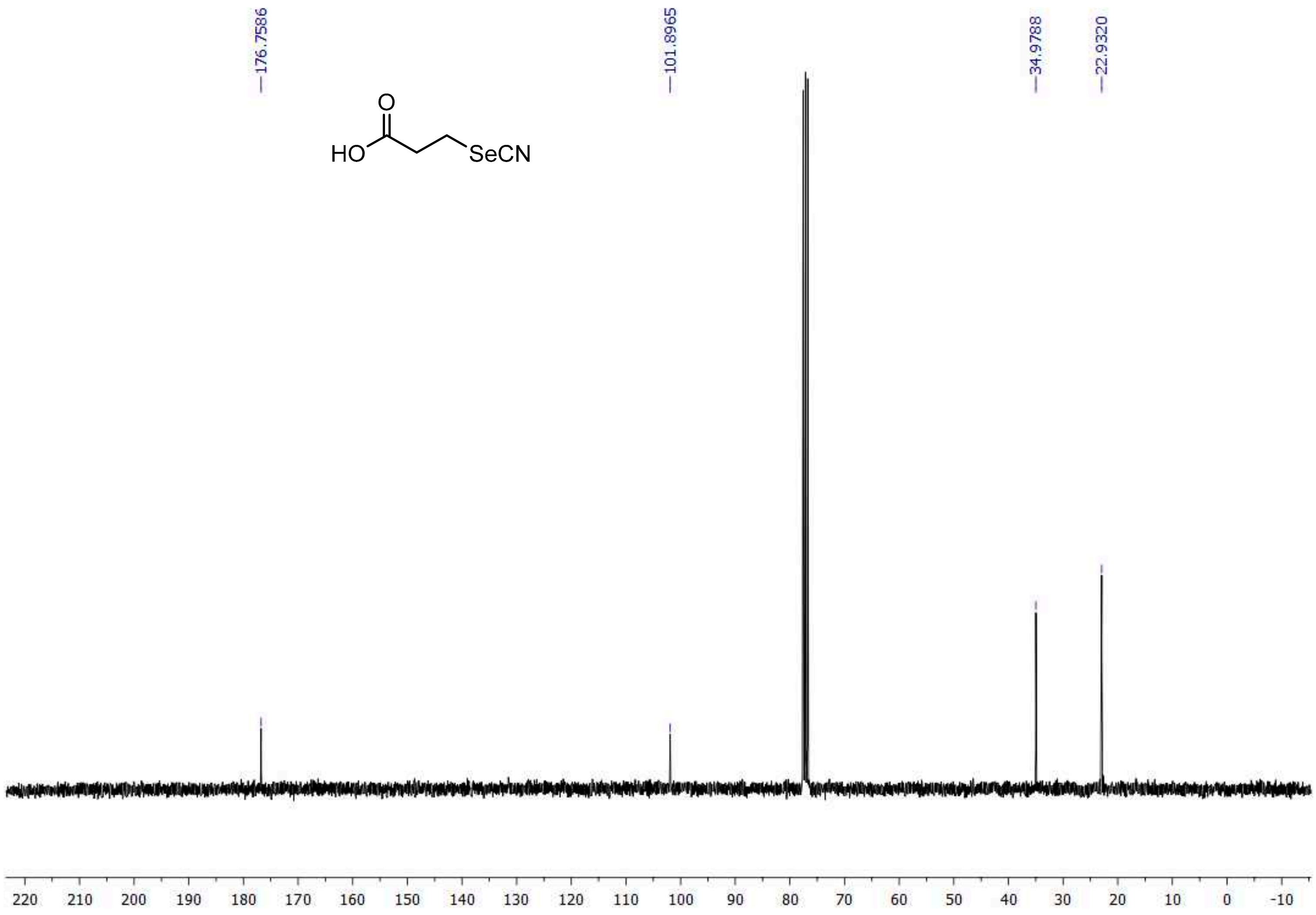

^{13}C- NMR (75.5 MHz, $CDCl_3$) of **19**

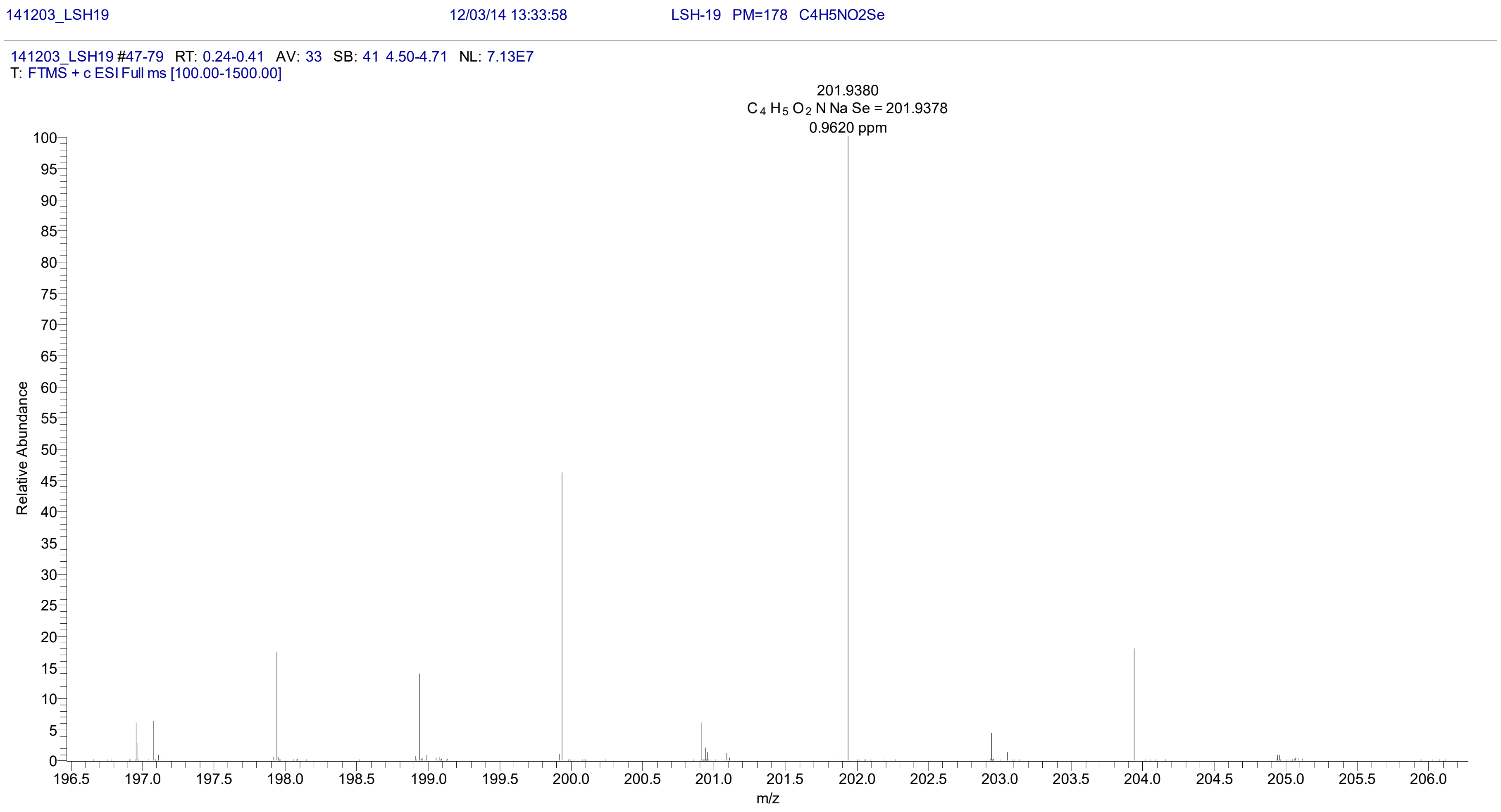

HRESI-MS spectrum of **19**

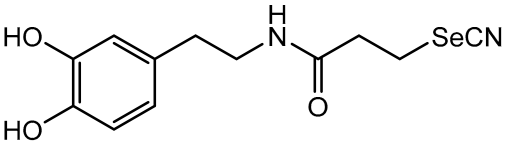

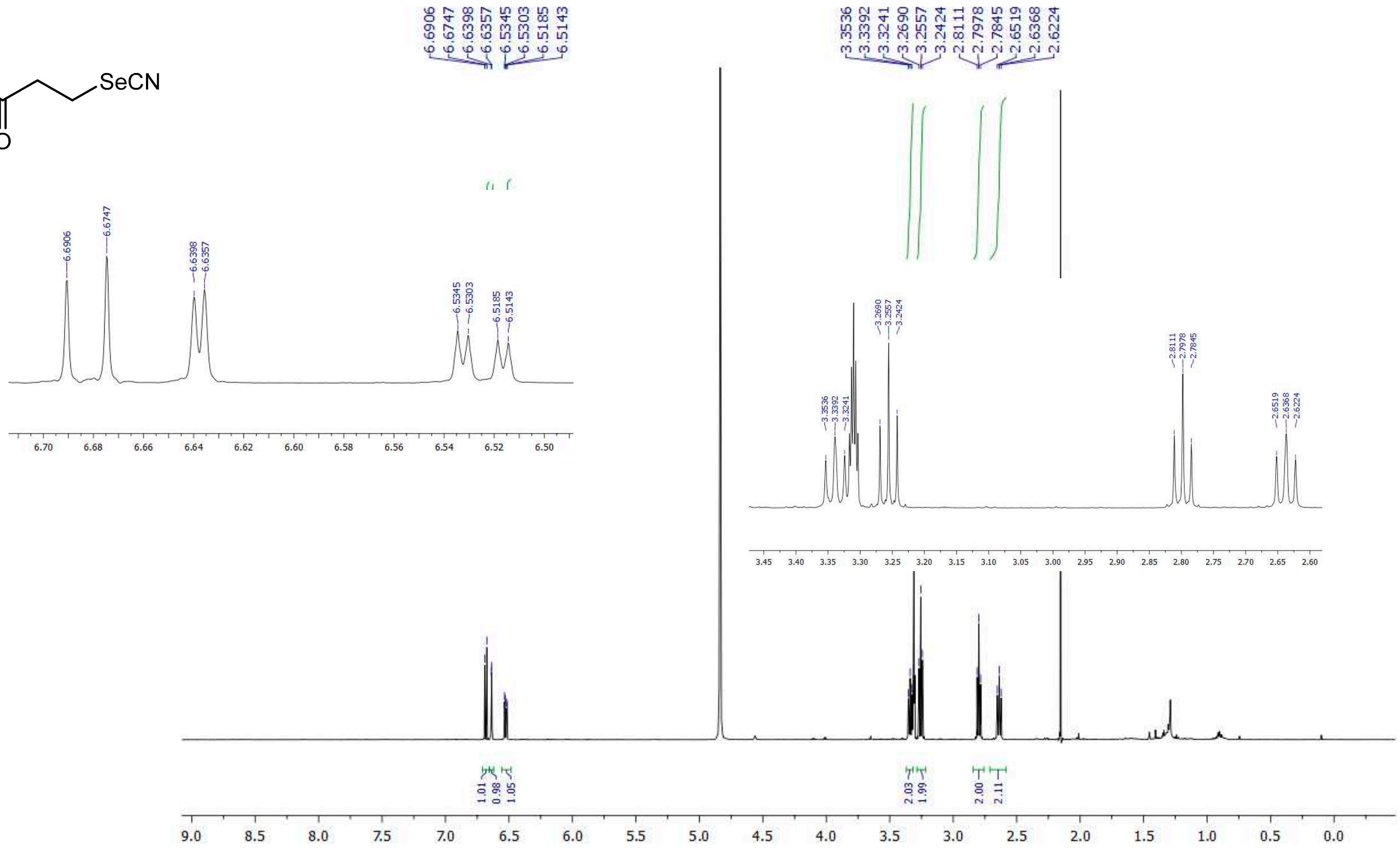

^{1}H- NMR (500 MHz, CD_3OD) of **20**

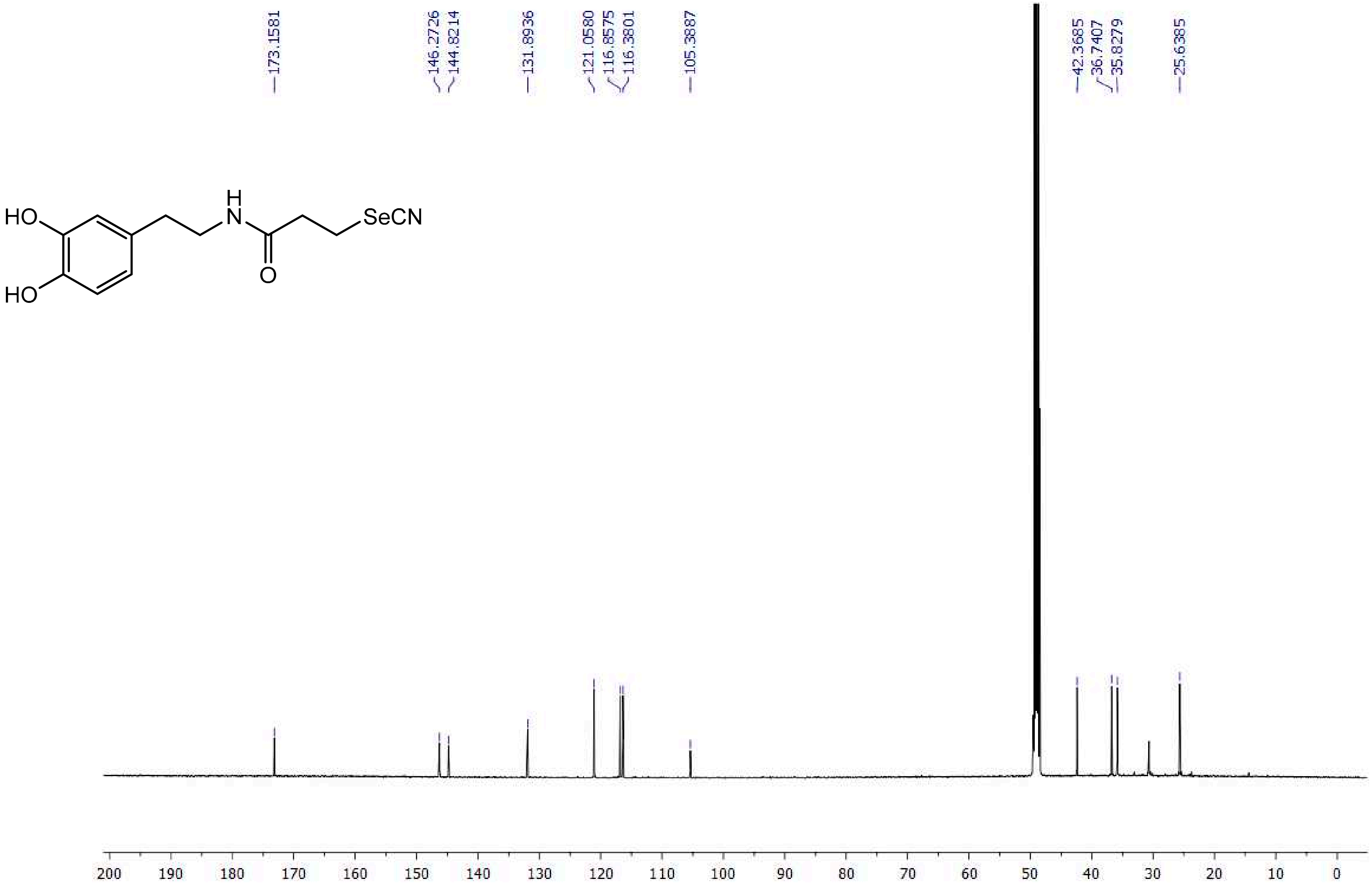

^{13}C- NMR (125.7 MHz, CD_3OD) of **20**

150513_LSH24 05/13/15 16:25:57 LSH-24 PM=314 C12H14N2O3Se

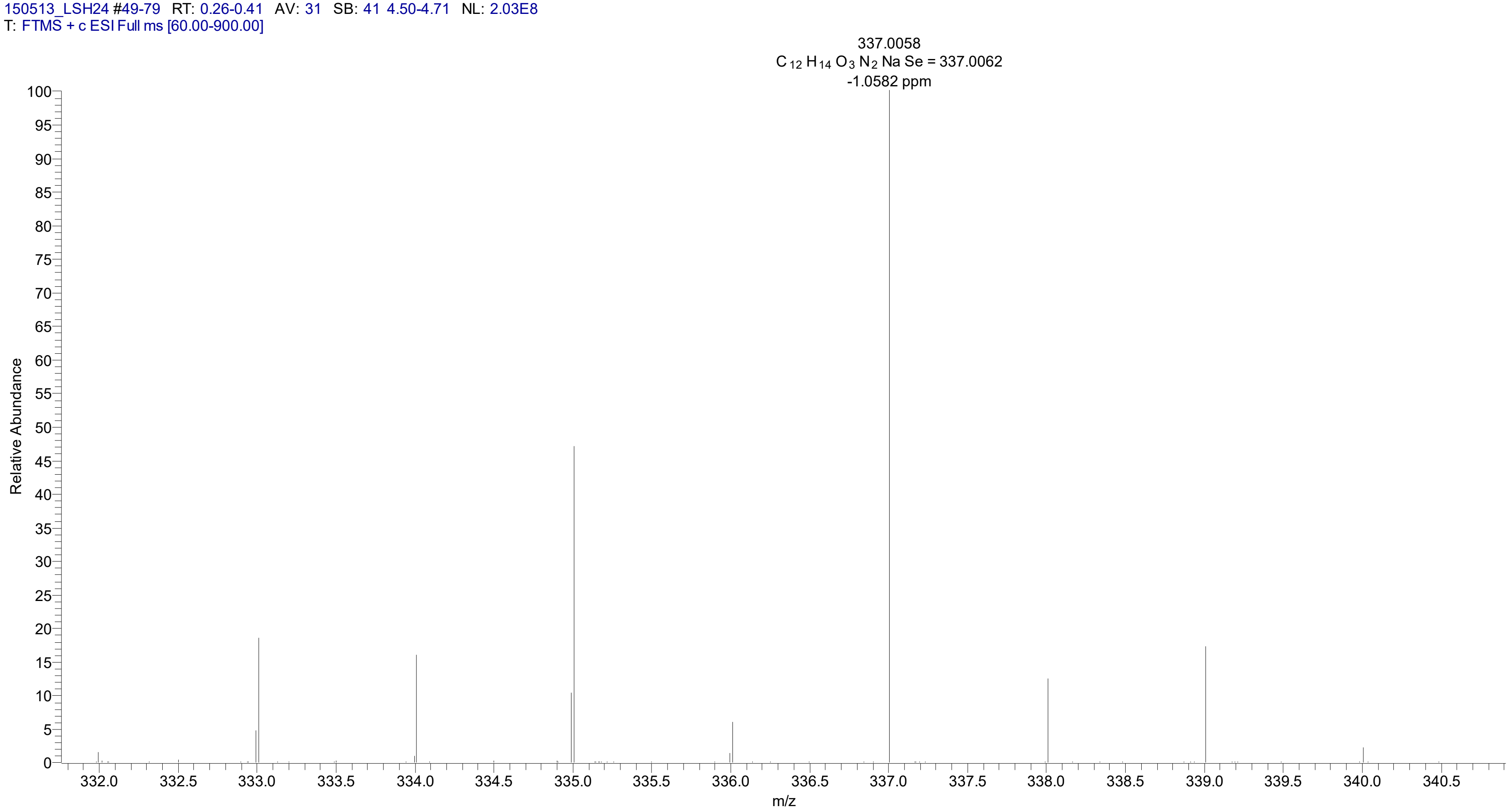

HRESI-MS spectrum of **20**

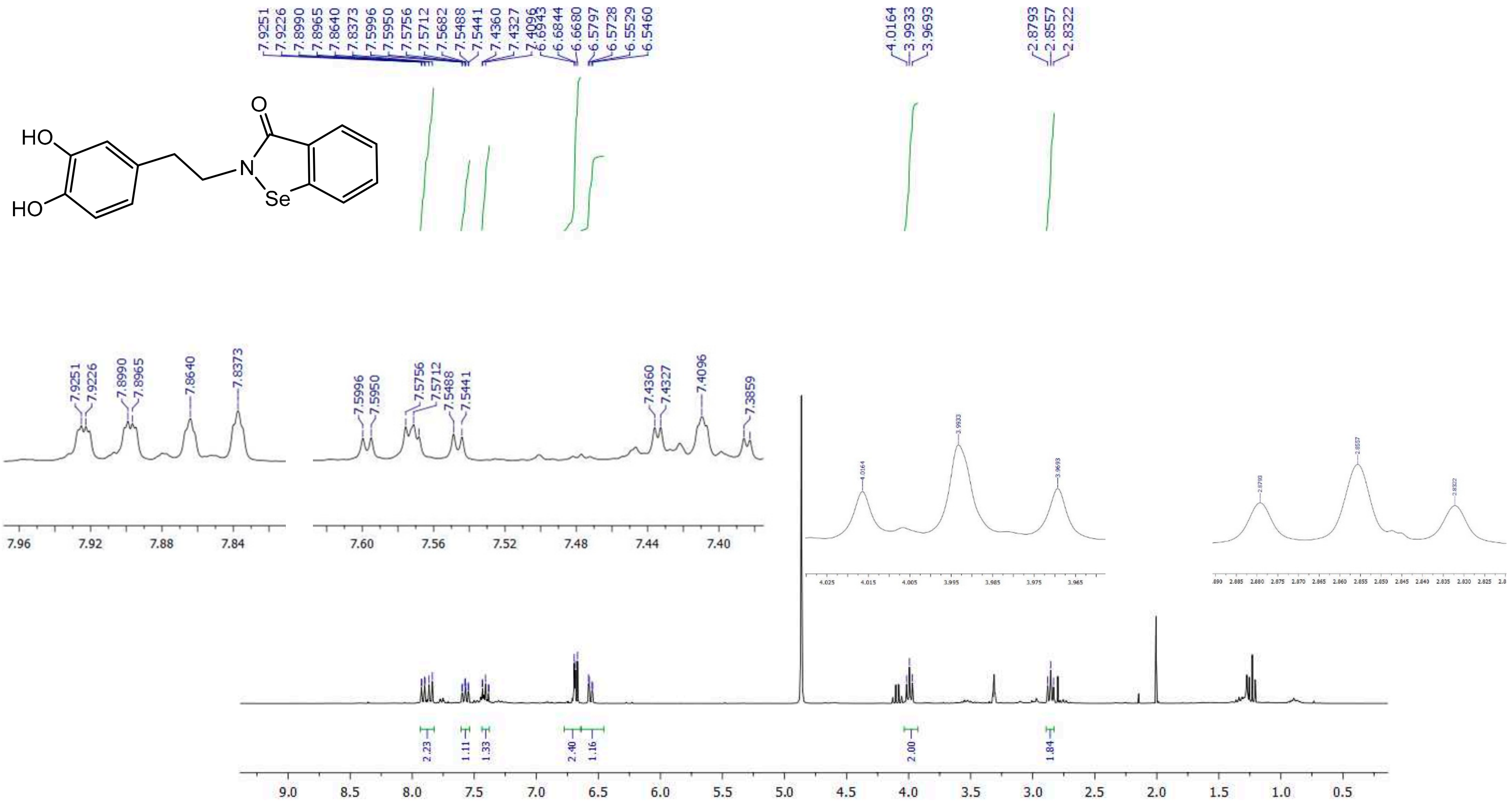

^{1}H- NMR (300 MHz, CD_3OD) of **26**

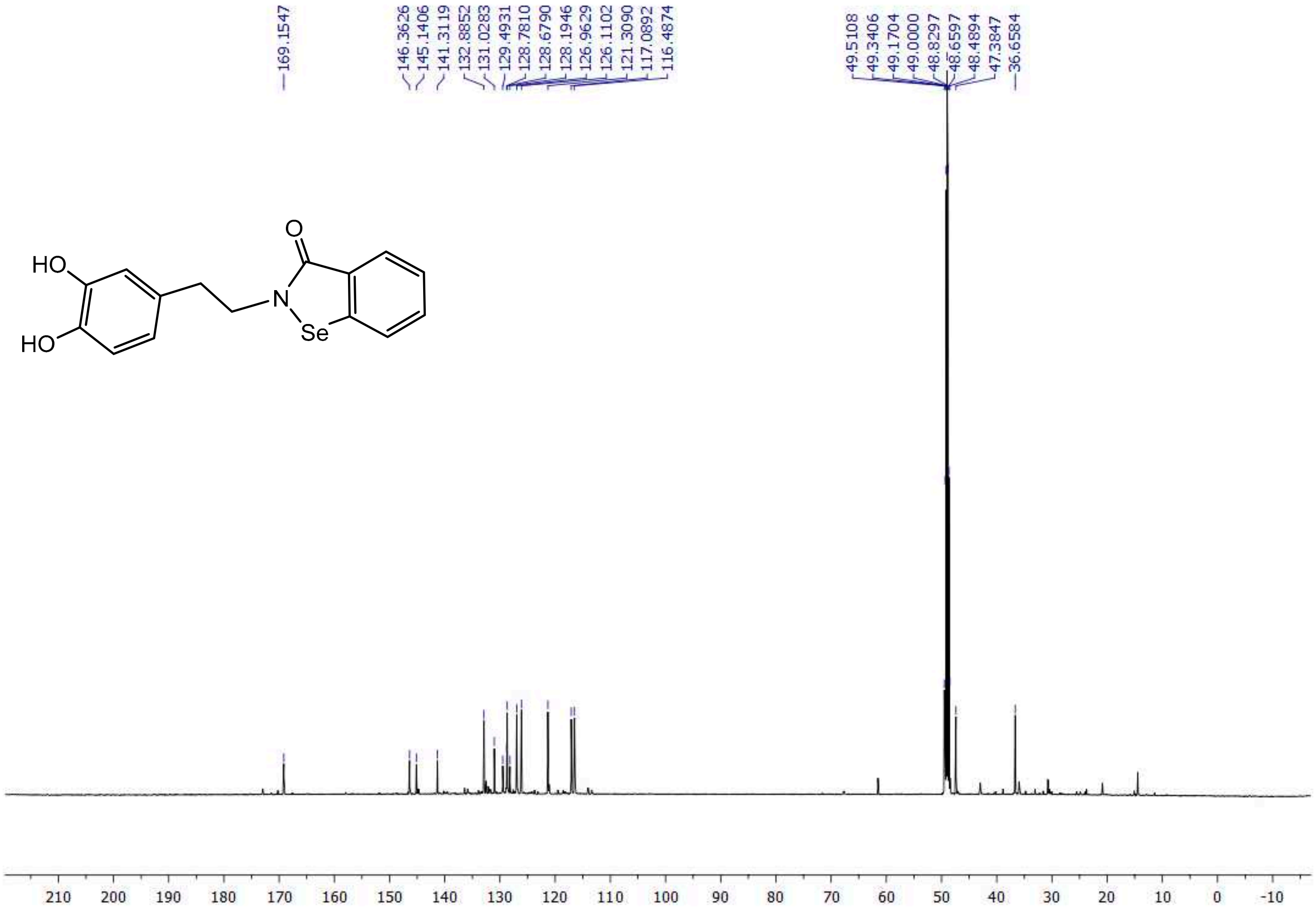

^{13}C- NMR (125.7 MHz, CD_3OD) of **26**

BG_150513_LSH49 05/13/15 17:52:18 LSH-49 PM=335 C15H13NO3Se

BG_150513_LSH49 #38-67 RT: 0.20-0.35 AV: 30 SB: 40 4.50-4.71 NL: 4.74E7
T: FTMS + c ESI Full ms [60.00-900.00]

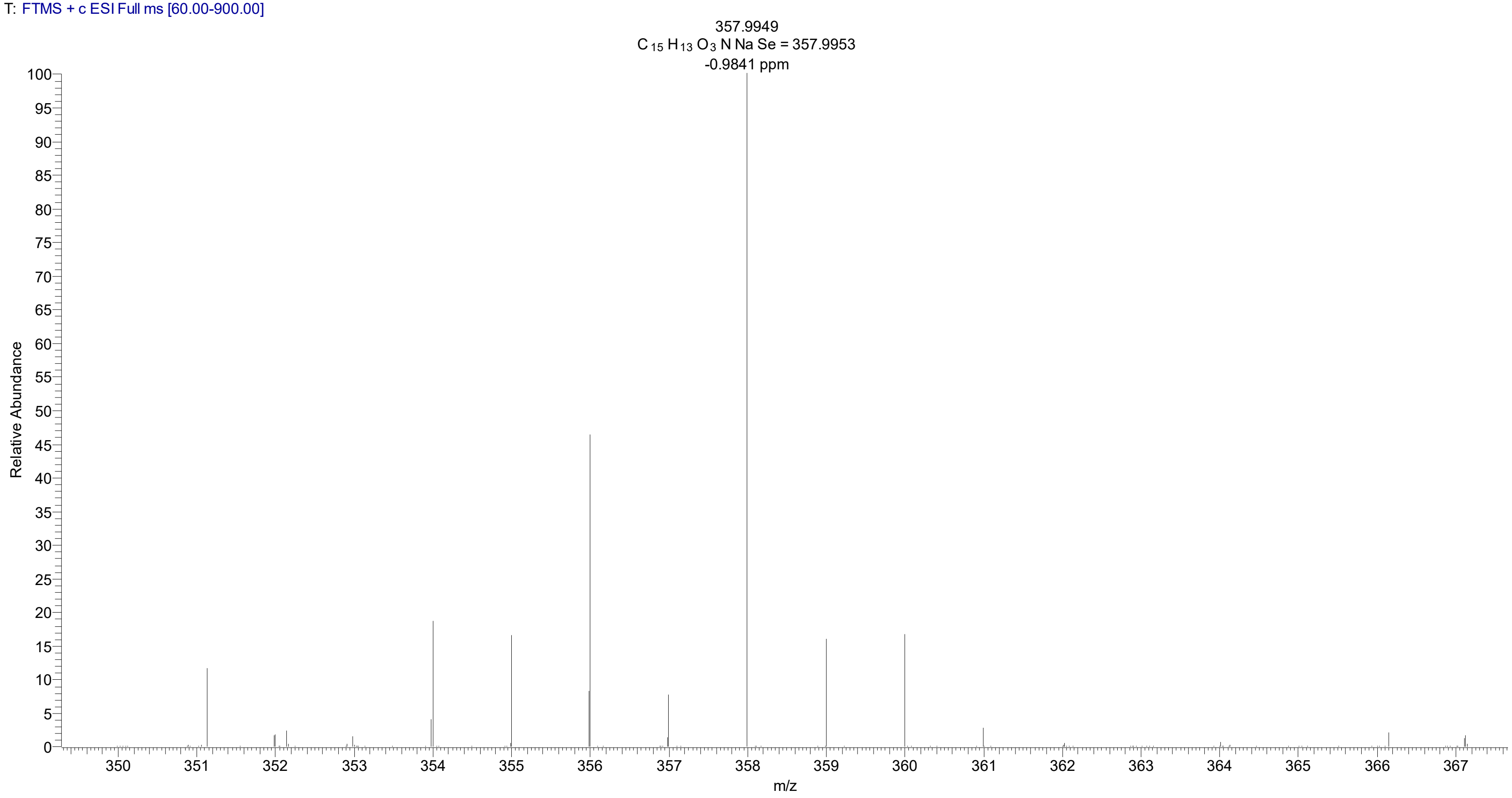

HRESI-MS spectrum of **26**

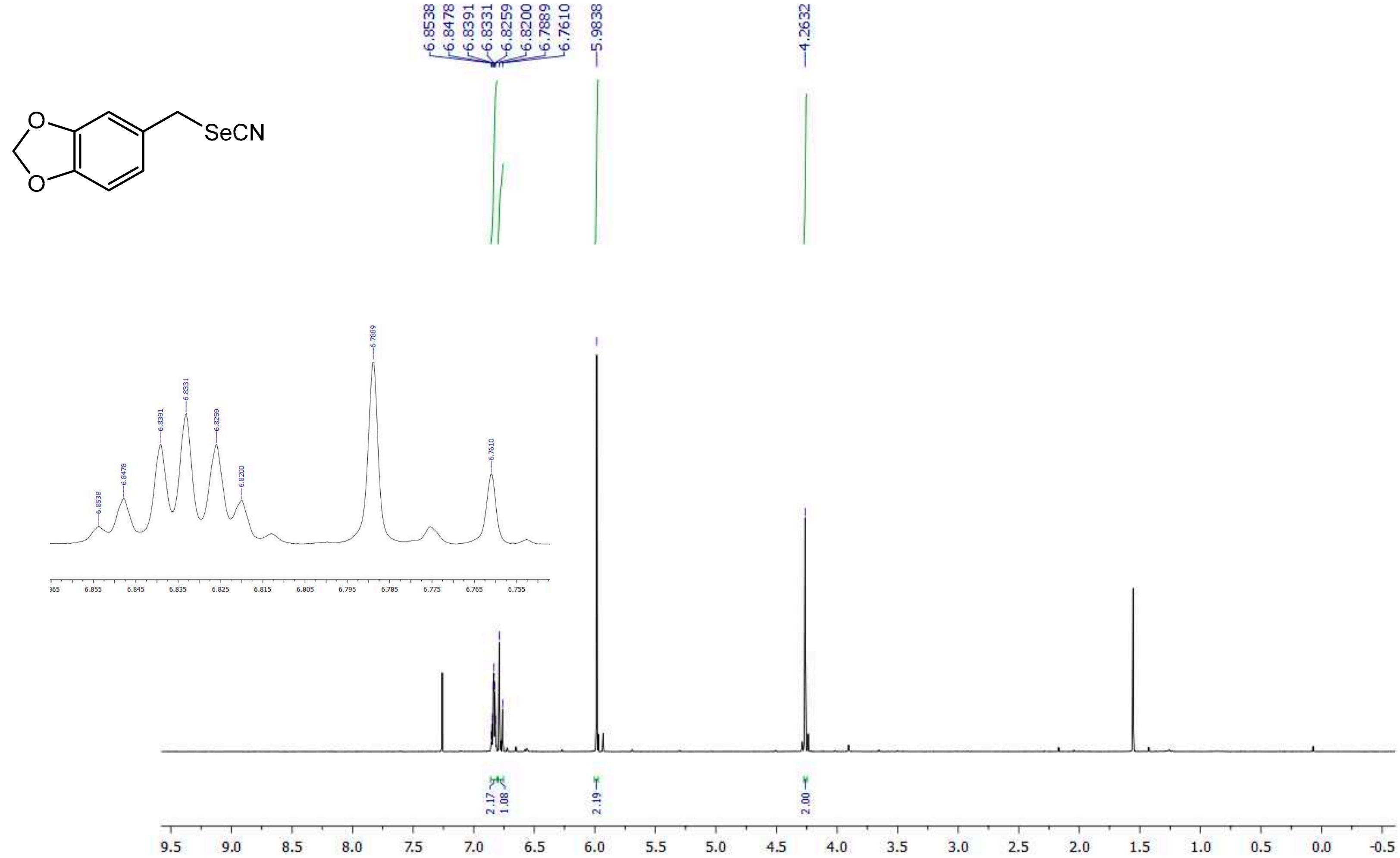

^{1}H- NMR (300 MHz, $CDCl_3$) of **35**

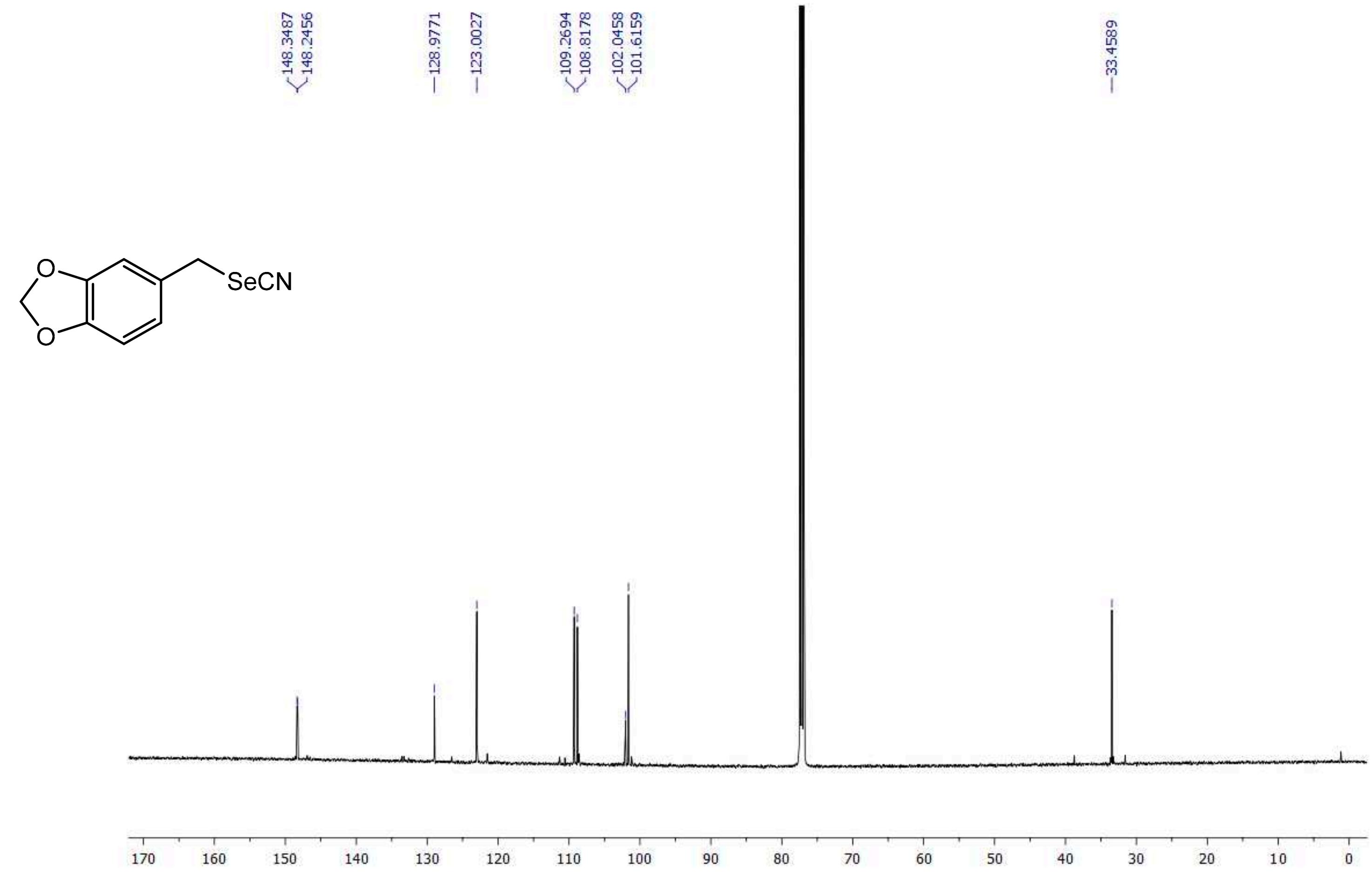

^{13}C- NMR (75.5 MHz, $CDCl_3$) of **35**

160511_RPP11 05/11/16 14:41:07 RPP-11 PM=240 C9H7NO2Se

160511_RPP11 #47-73 RT: 0.25-0.39 AV: 27 NL: 1.51E7
T: FTMS + c ESI Full ms [60.00-900.00]

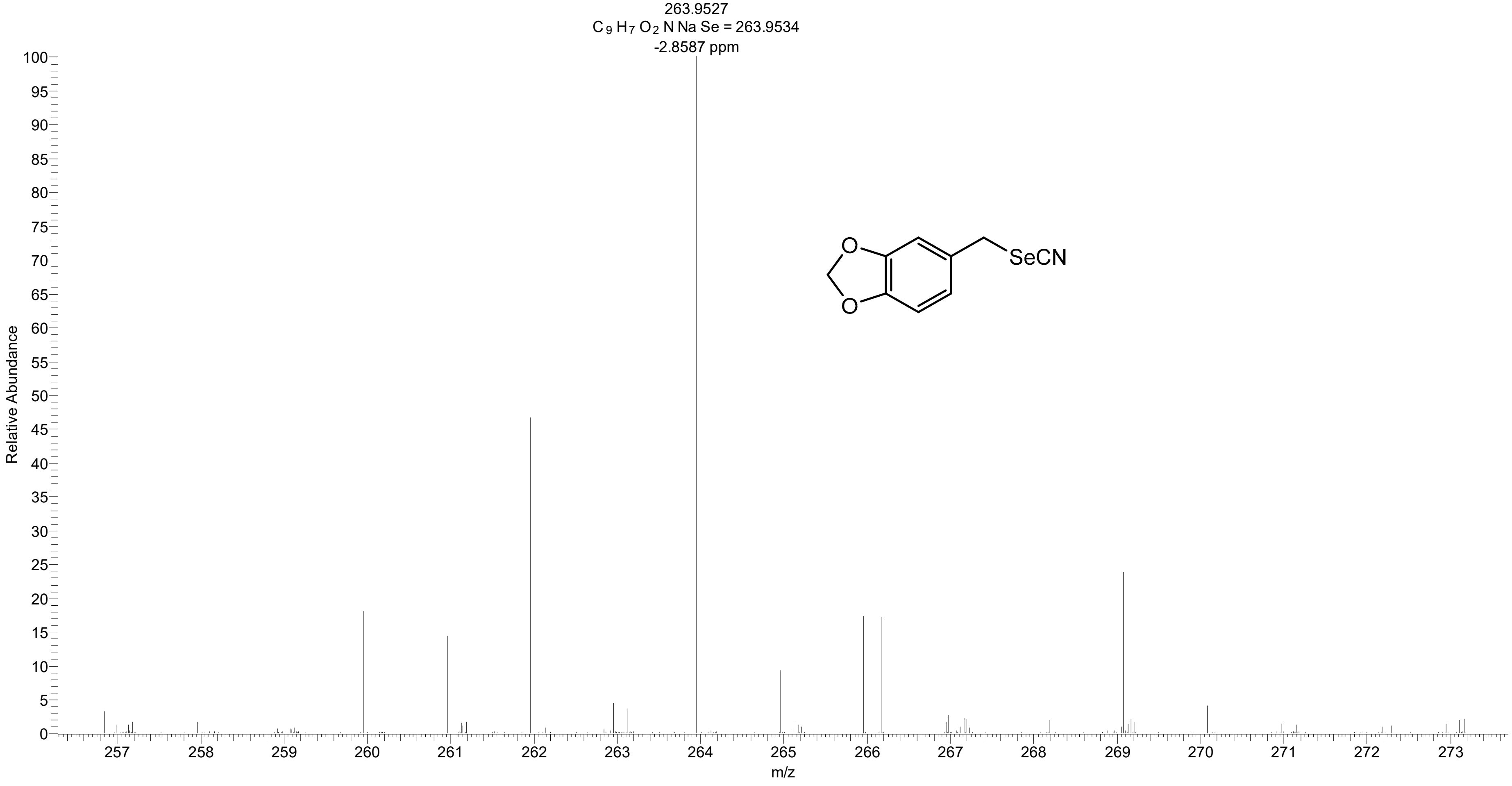

HRESI-MS spectrum of **35**

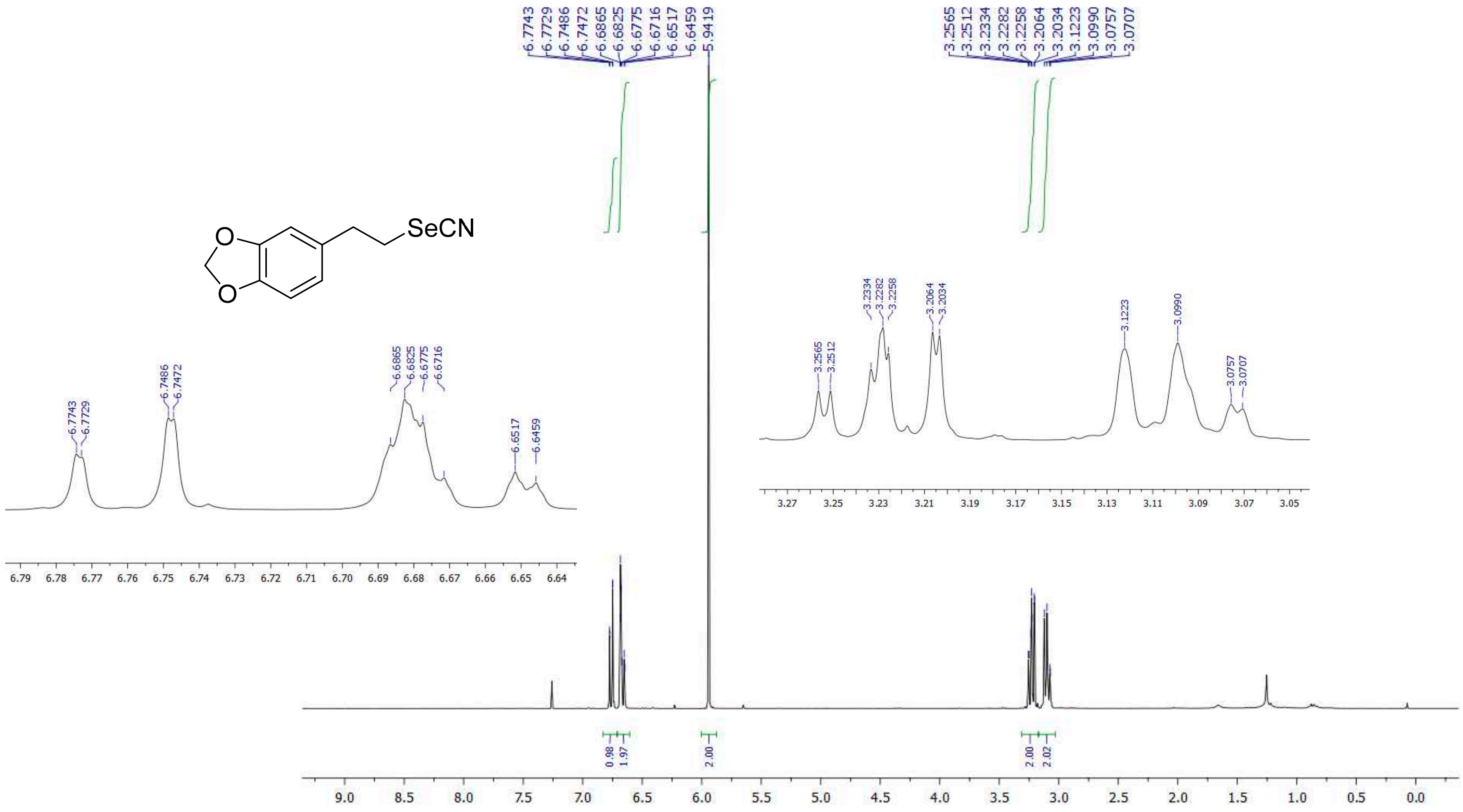

^{1}H- NMR (300 MHz, $CDCl_3$) of **36**

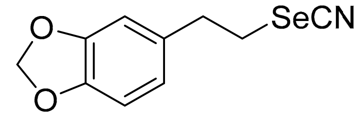

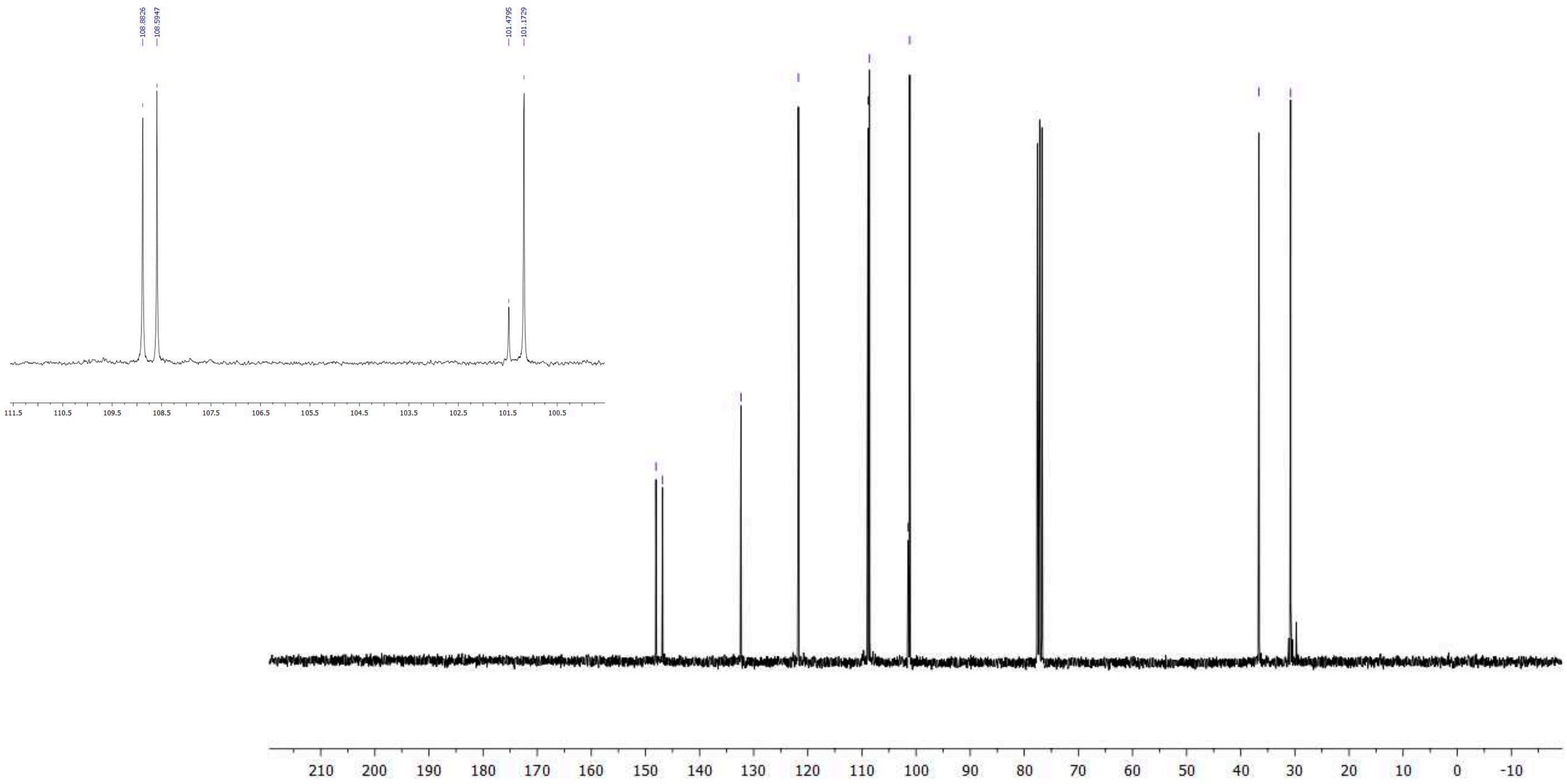

^{13}C- NMR (75.5 MHz, $CDCl_3$) of **36**

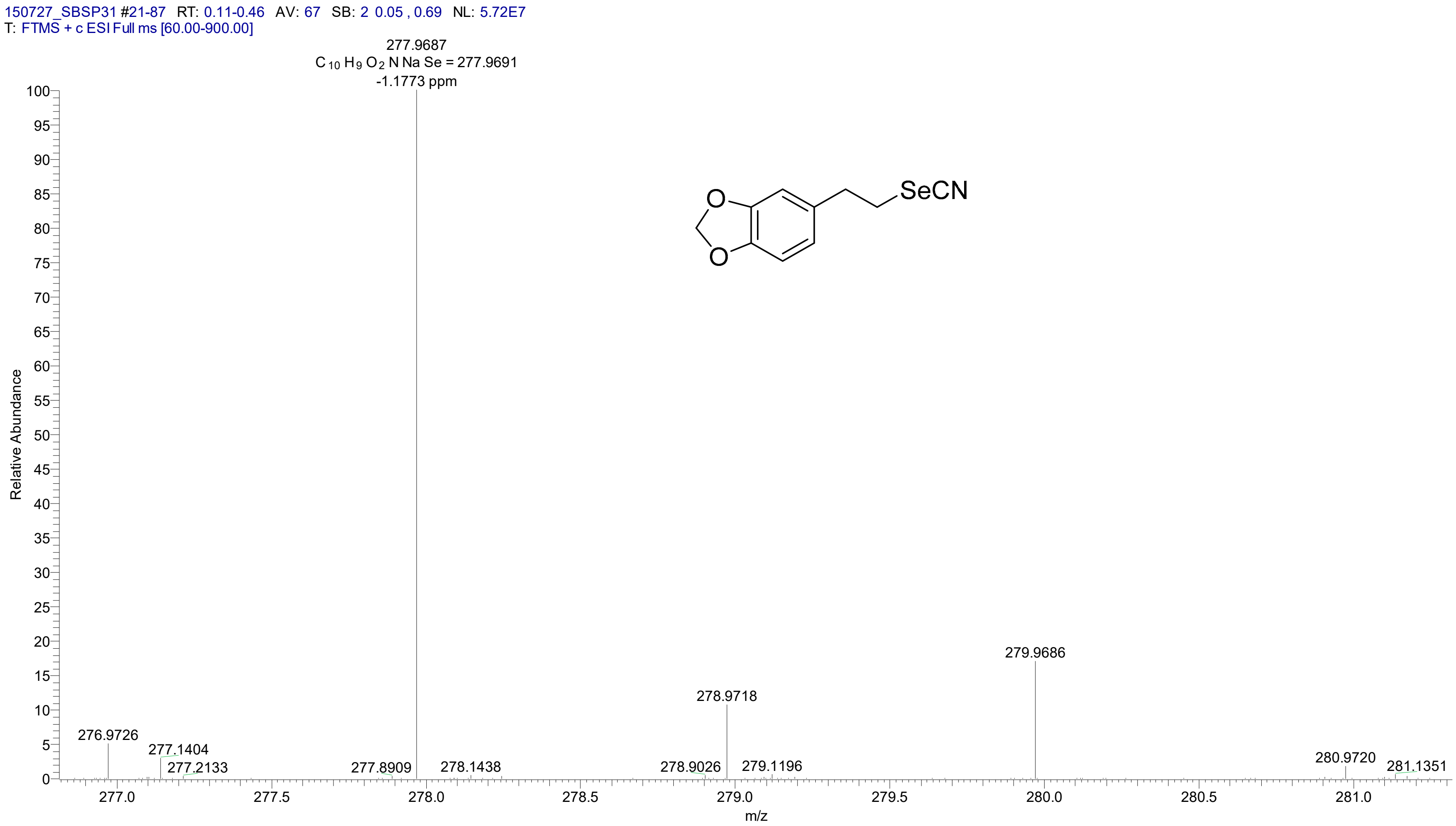

HRESI-MS spectrum of **36**

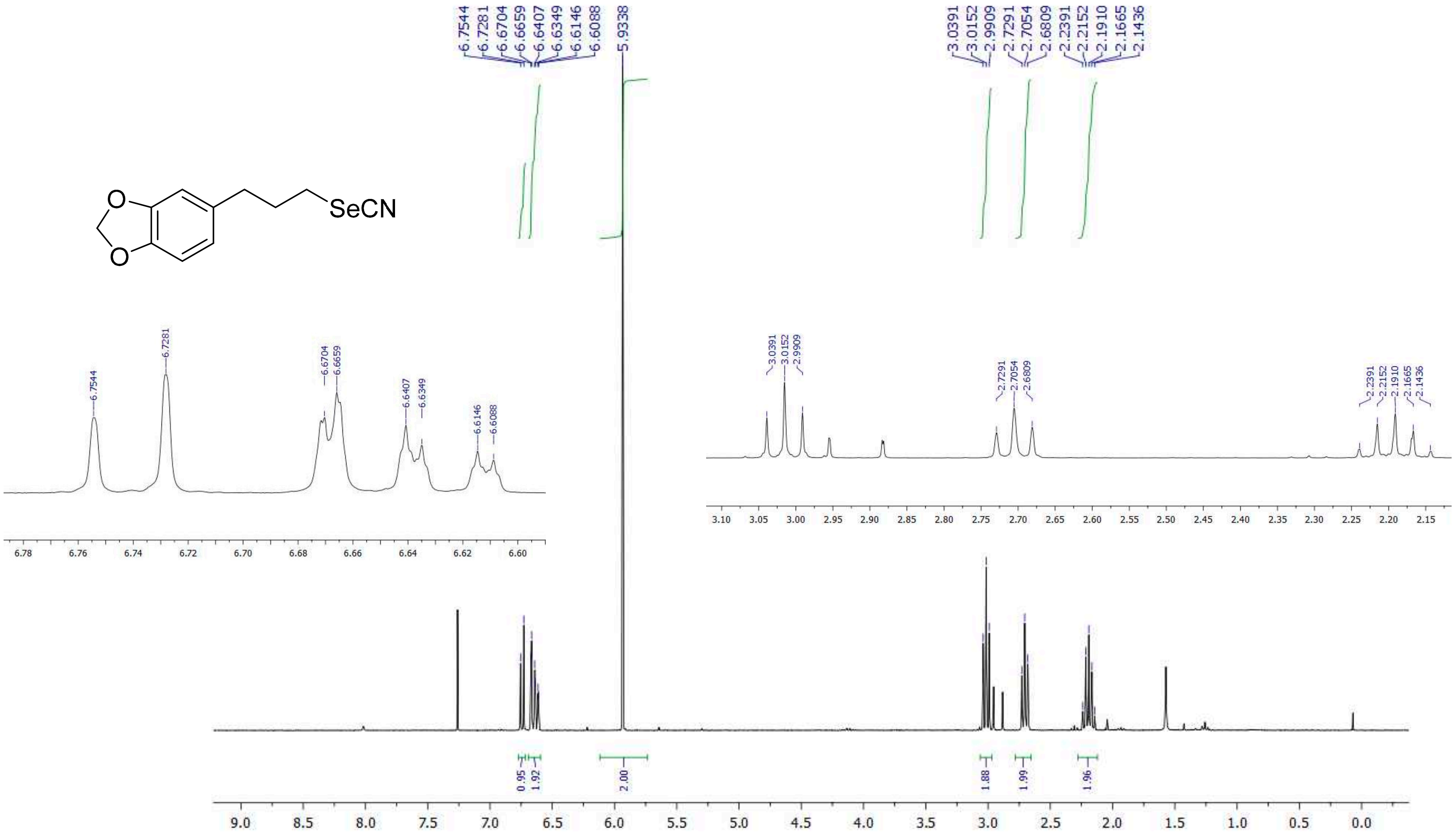

^{1}H- NMR (300 MHz, $CDCl_3$) of **37**

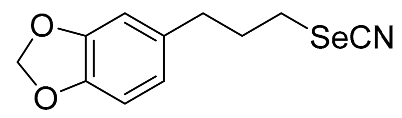

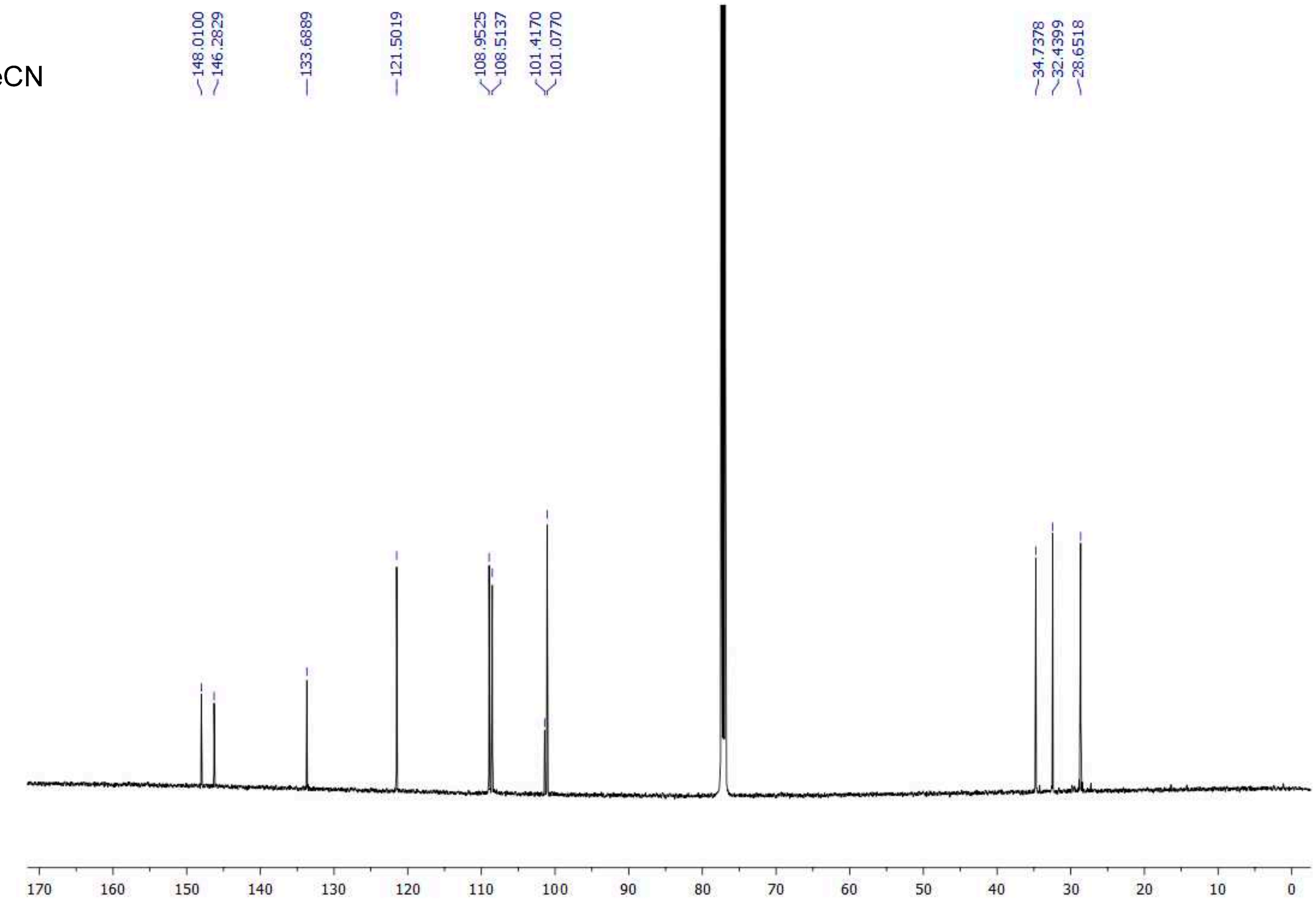

^{13}C- NMR (125.7 MHz, $CDCl_3$) of **37**

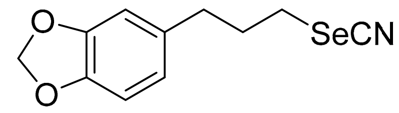

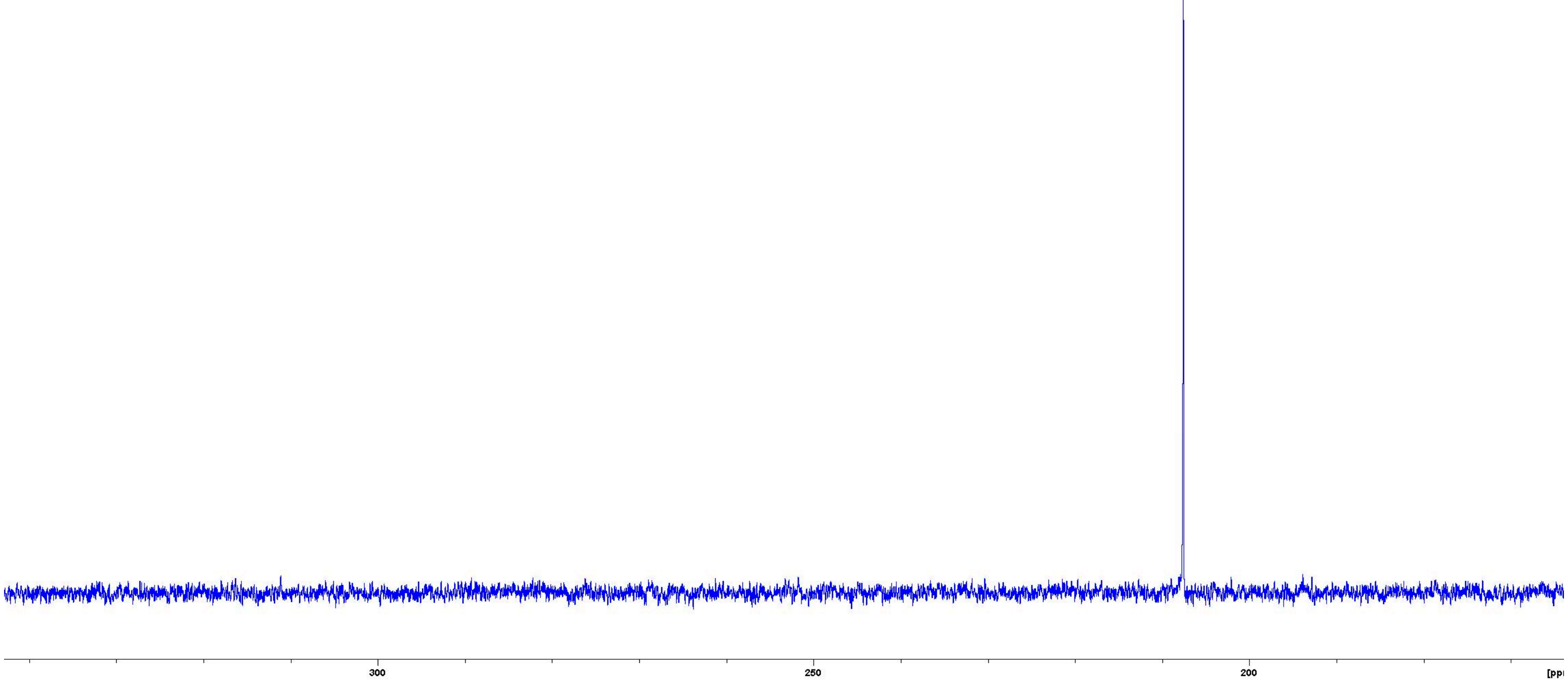

^{77}Se- NMR (95 MHz, $CDCl_3$) of **37**

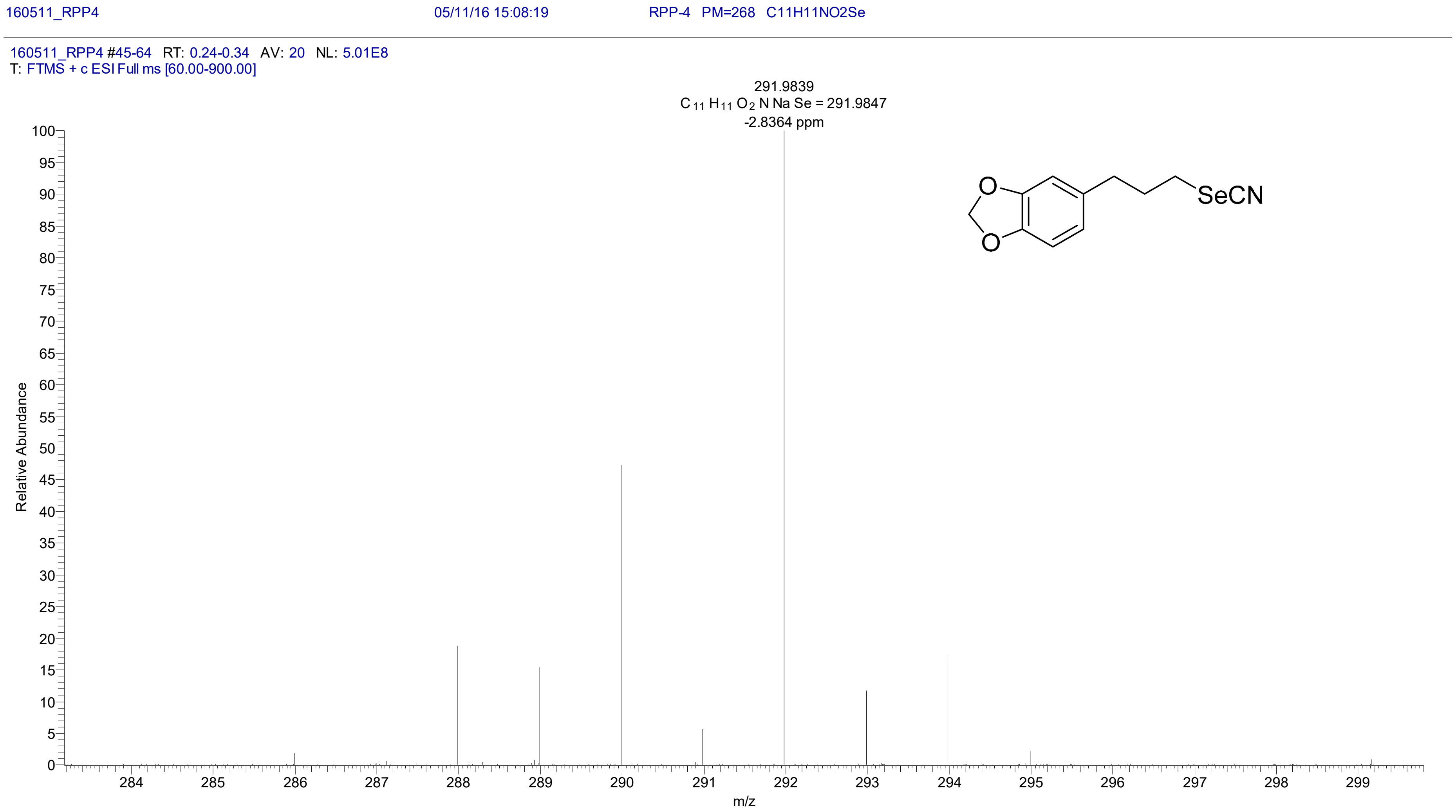

HRESI-MS spectrum of **37**

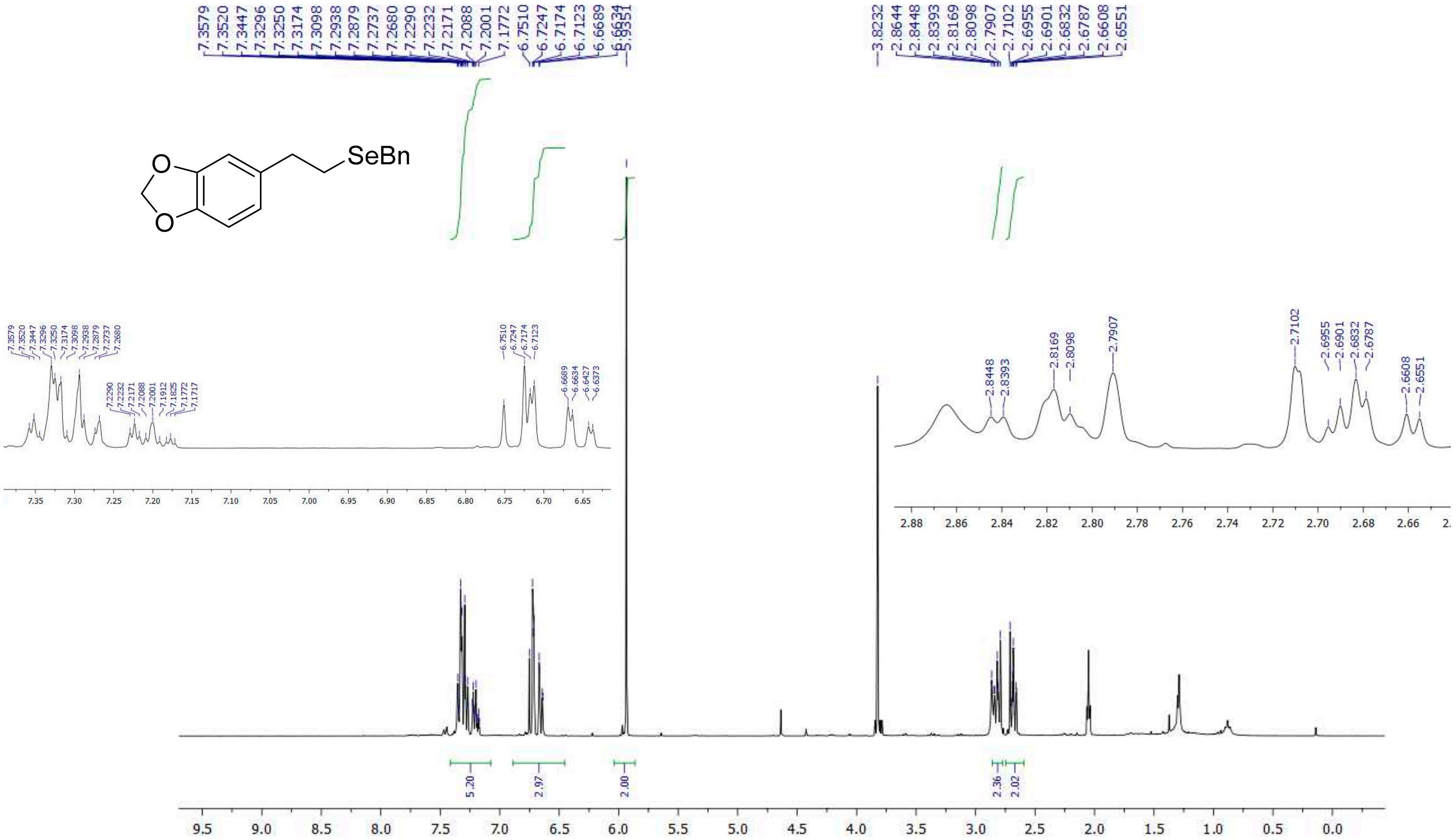

^{1}H- NMR (300 MHz, $(CD_3)_2CO$) of **38**

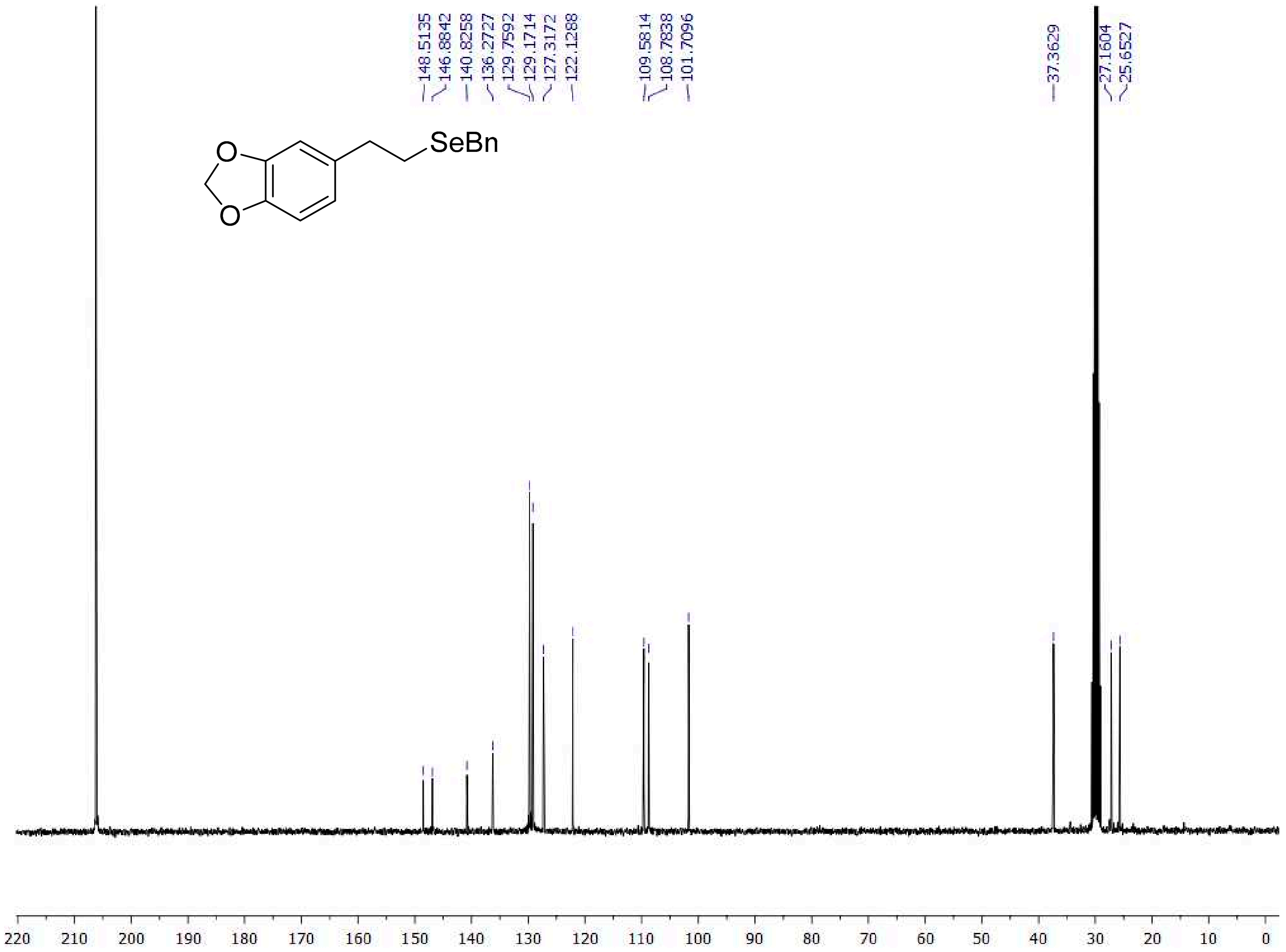

^{13}C- NMR (75.5 MHz, $(CD_3)_2CO$) of **38**

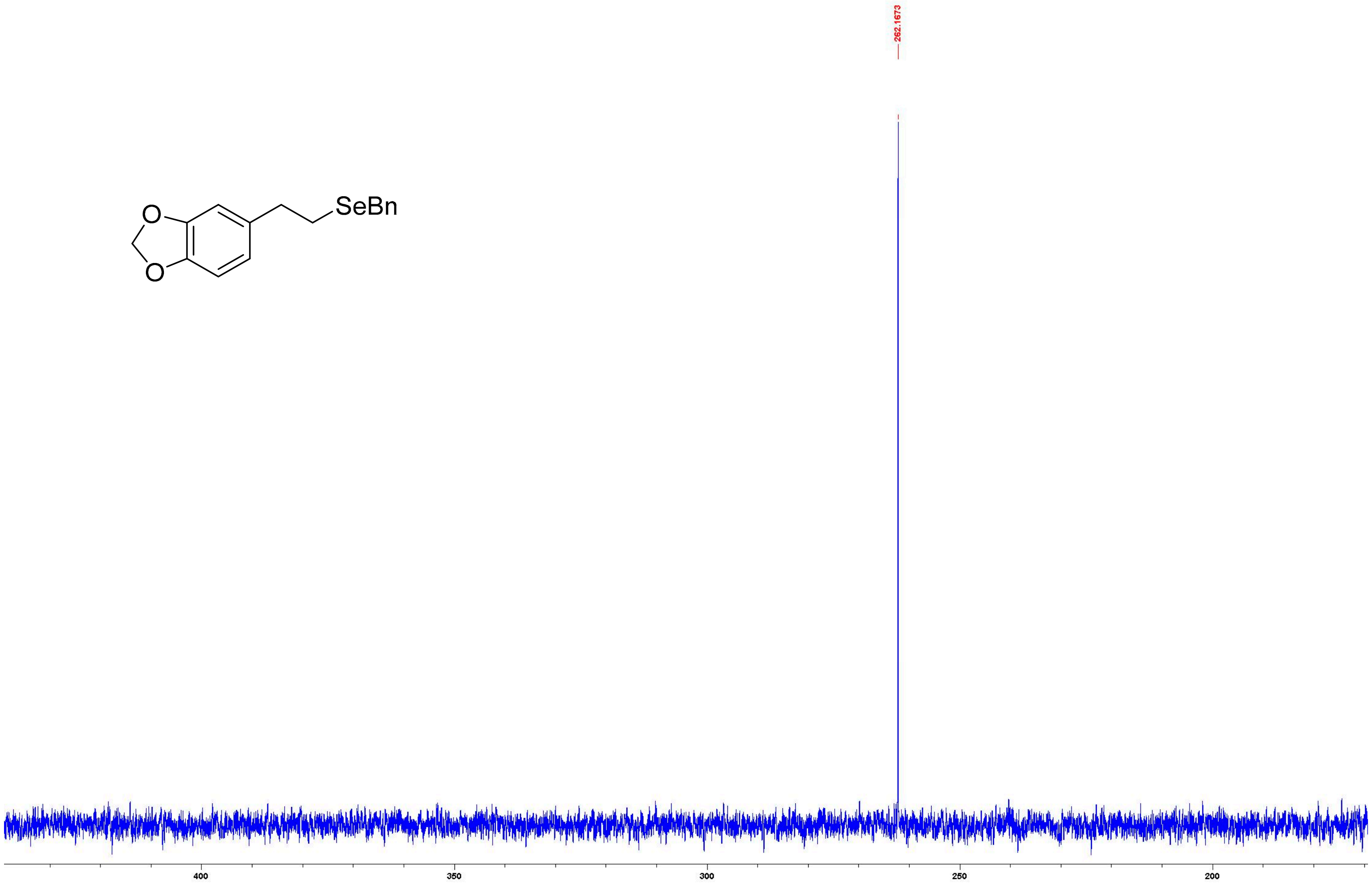

^{77}Se- NMR (95 MHz, $CDCl_3$) of **38**

C:\Users\jesus\Desktop\BG_150923_SBSPP34 09/24/15 10:36:13 SBS PP34 PM=320 C16H16O2Se

BG_150923_SBSPP34 #49-63 RT: 0.26-0.34 AV: 15 NL: 7.74E6
T: FTMS + c ESI Full ms [60.00-900.00]

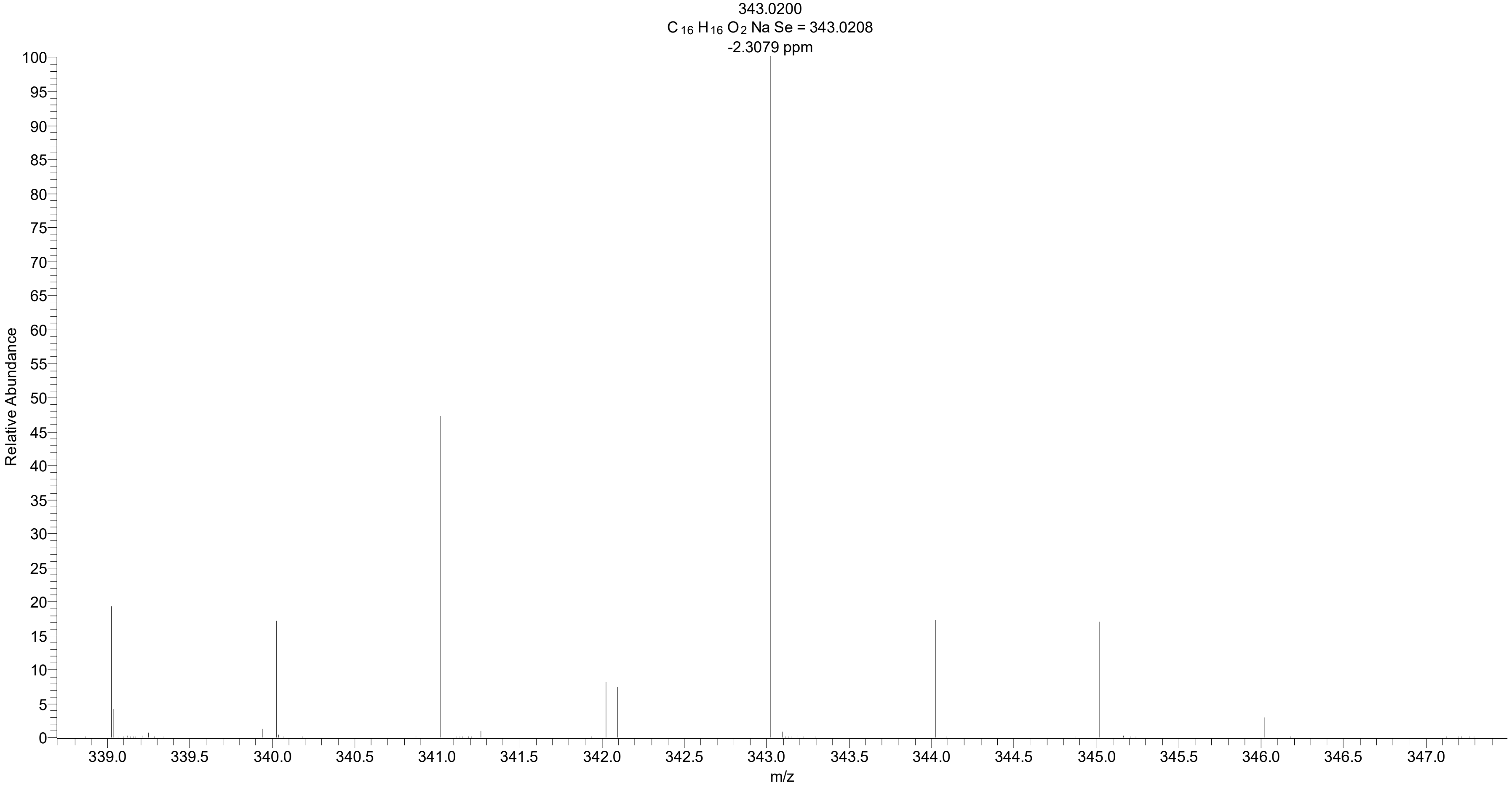

HRESI-MS spectrum of **38**

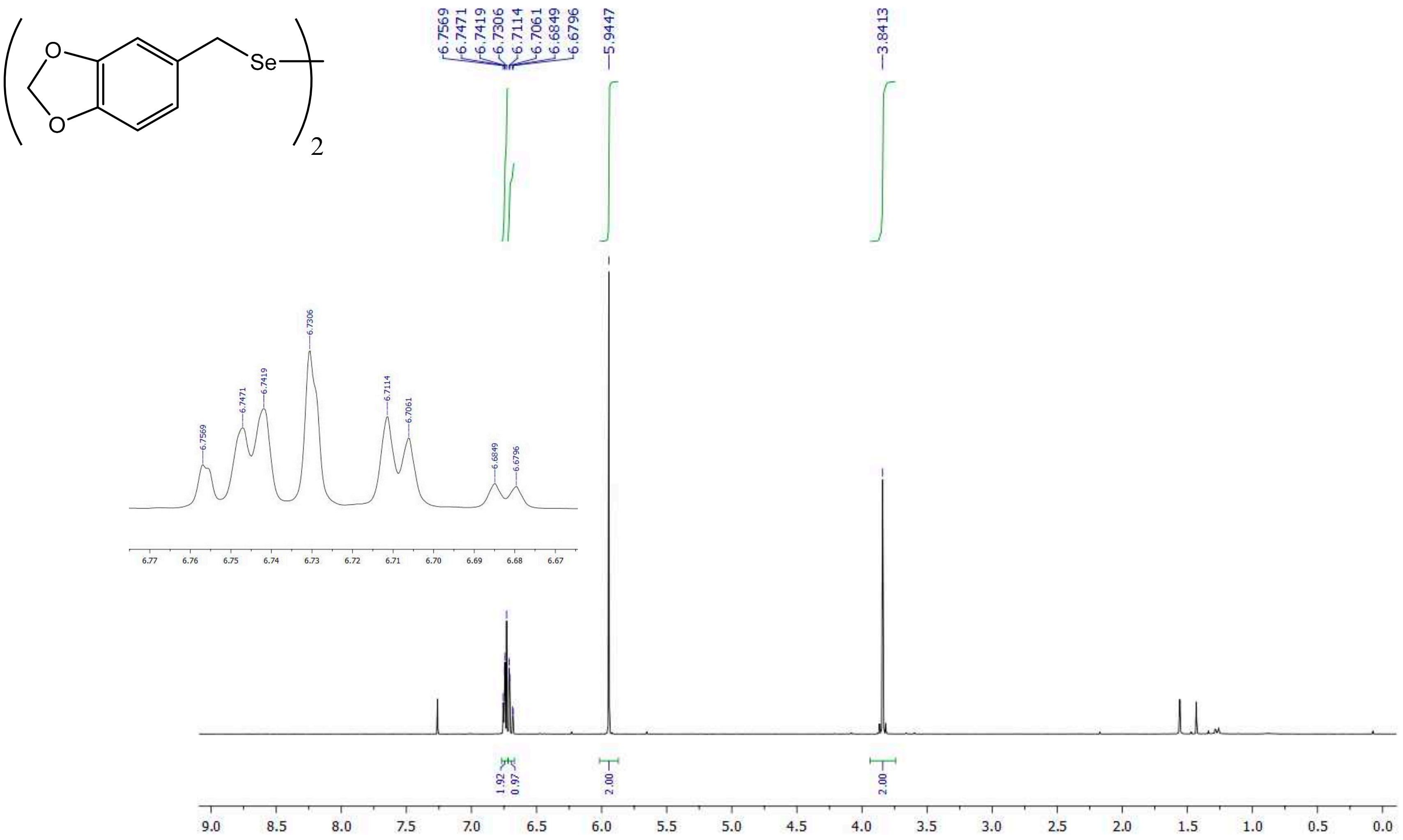

^{1}H- NMR (300 MHz, $CDCl_3$) of **39**

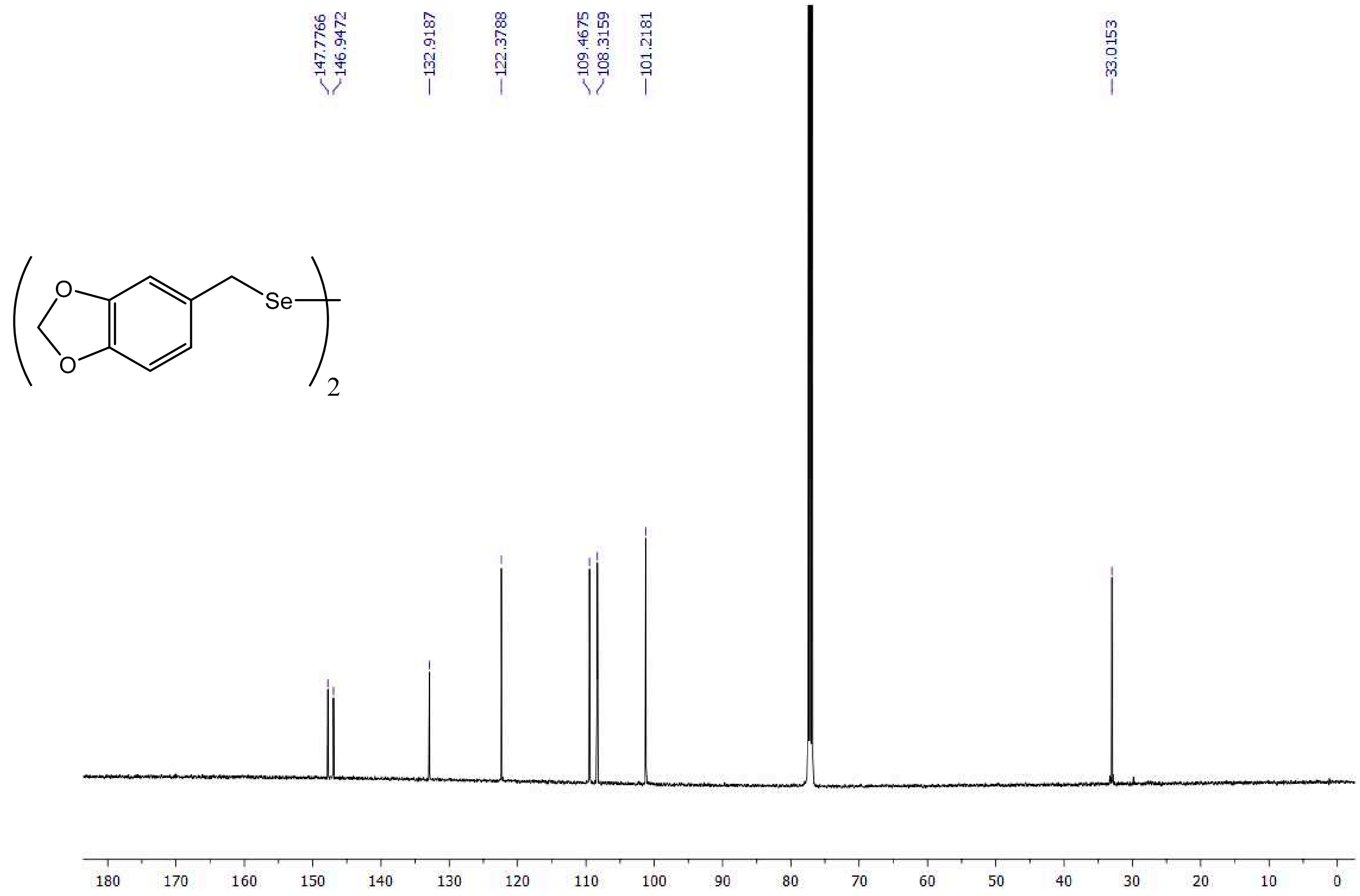

^{13}C- NMR (125.7 MHz, $CDCl_3$) of **39**

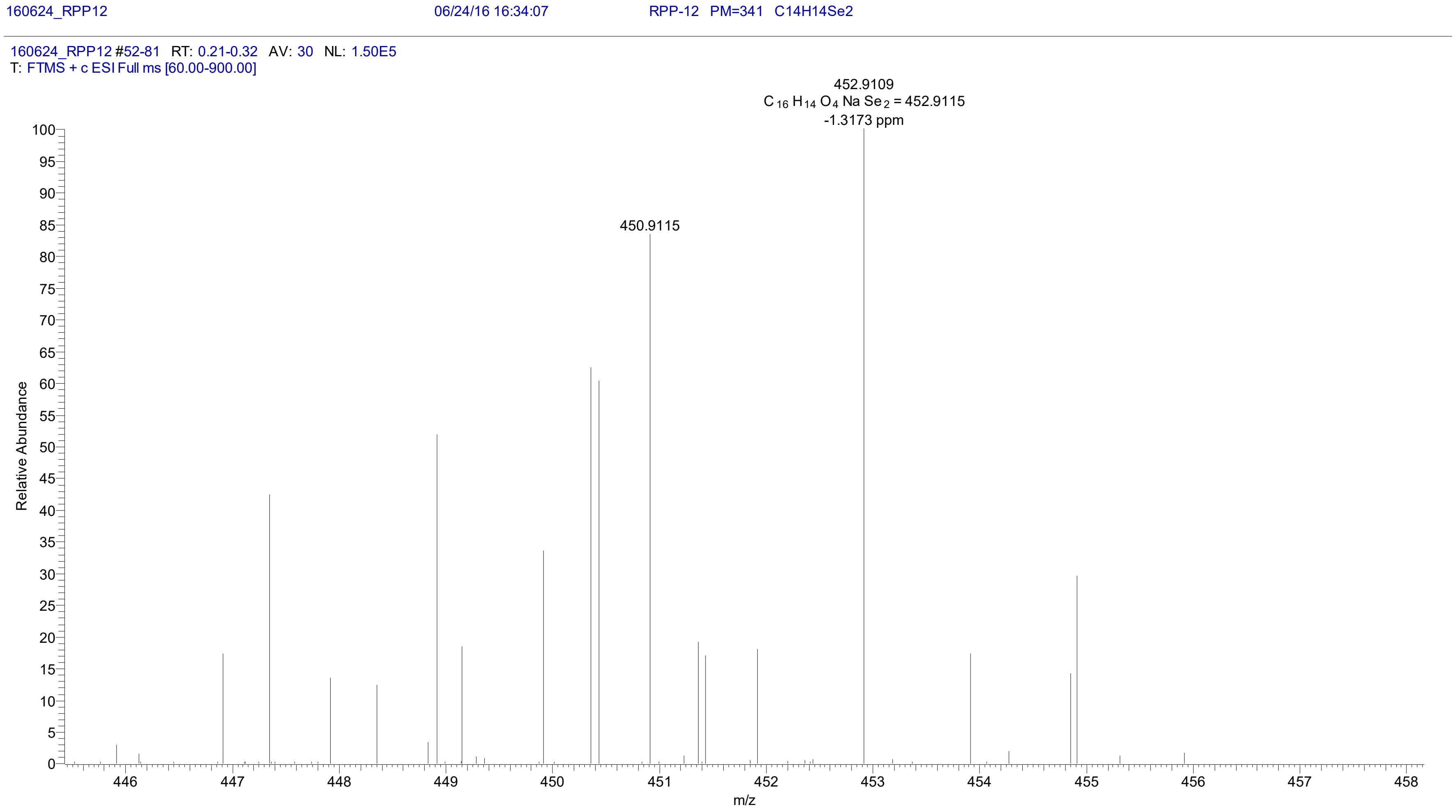

HRESI-MS spectrum of **39**

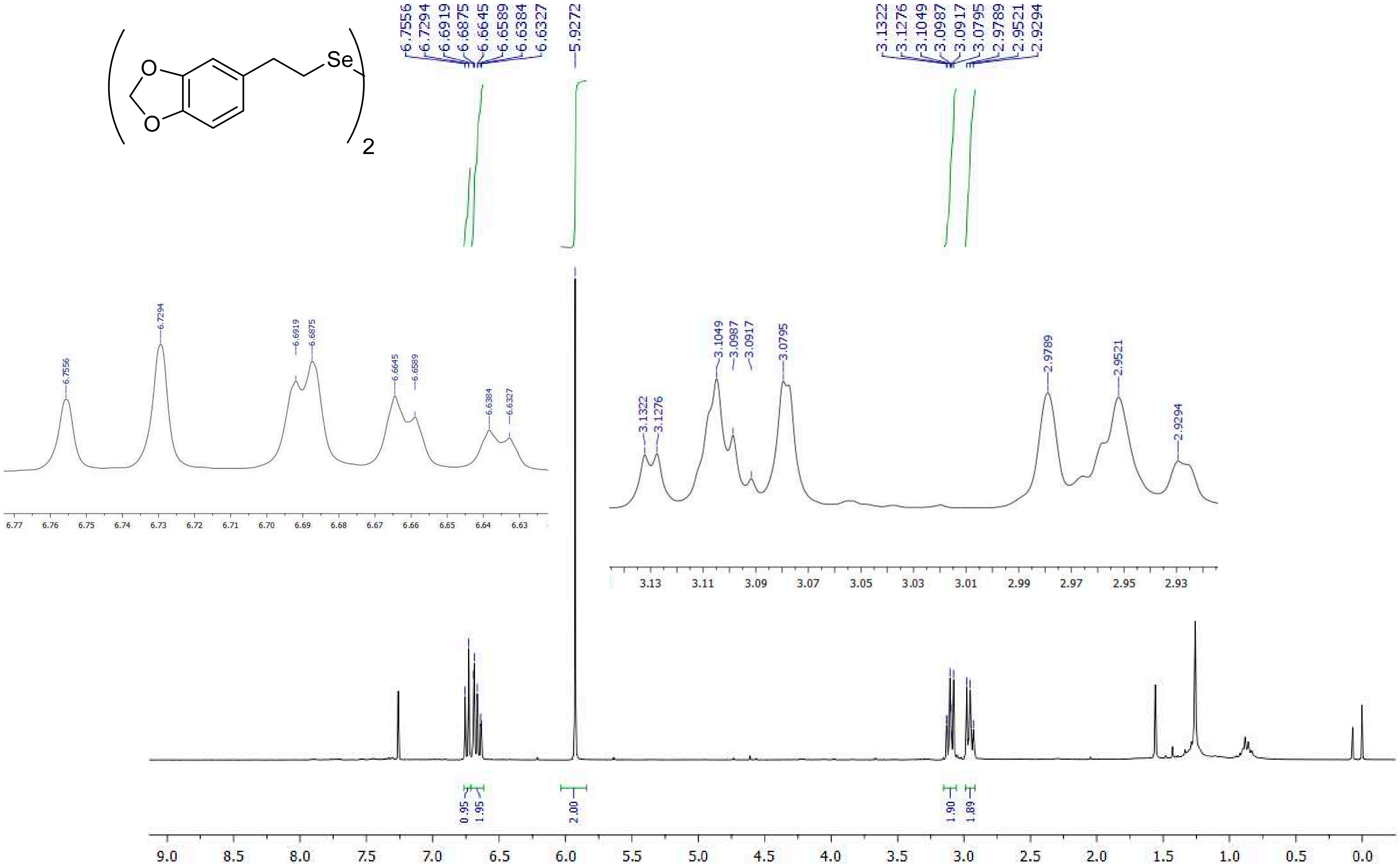

^{1}H- NMR (300 MHz, CDCl3) of **40**

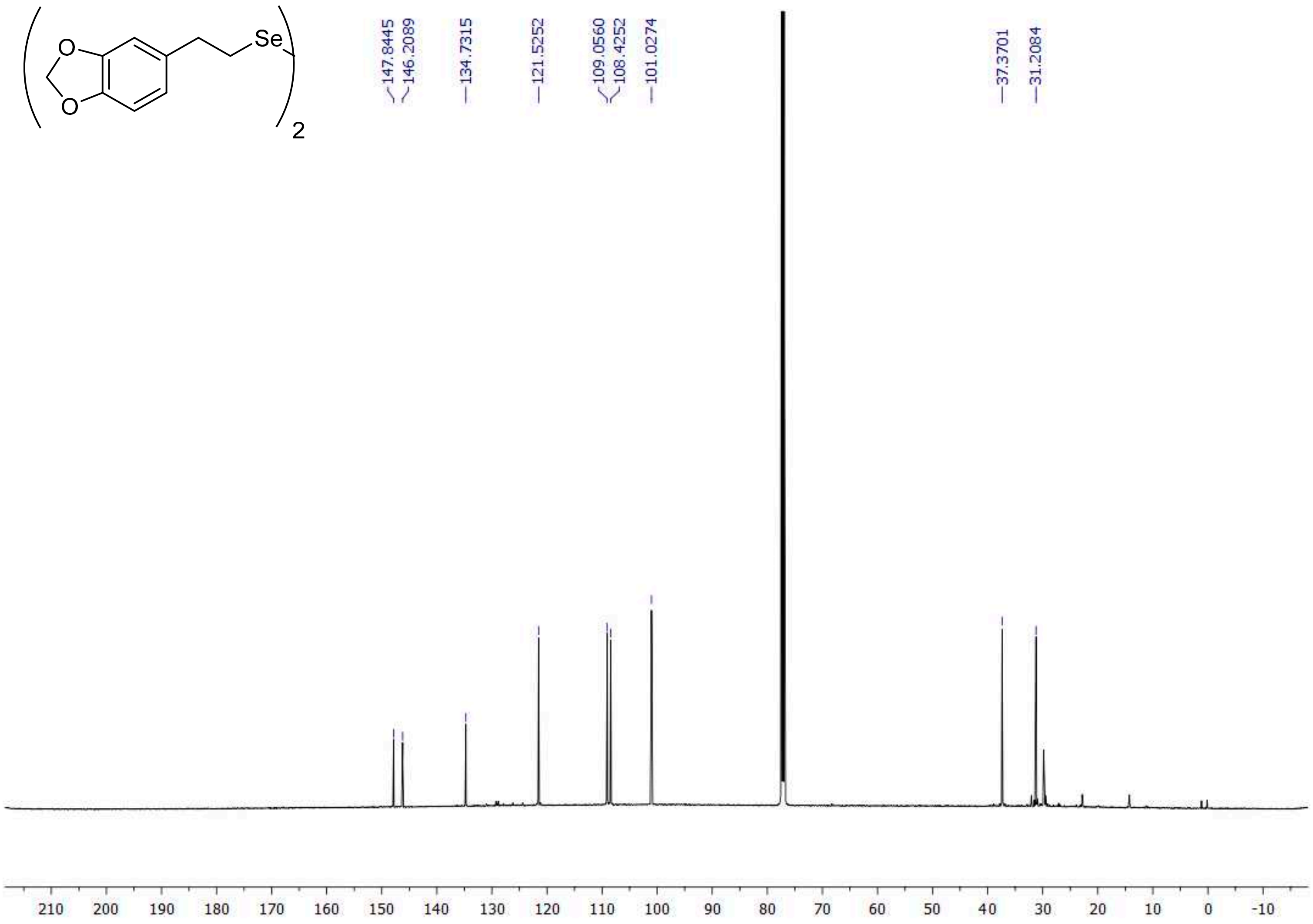

^{13}C- NMR (125.7 MHz, $CDCl_3$) of **40**

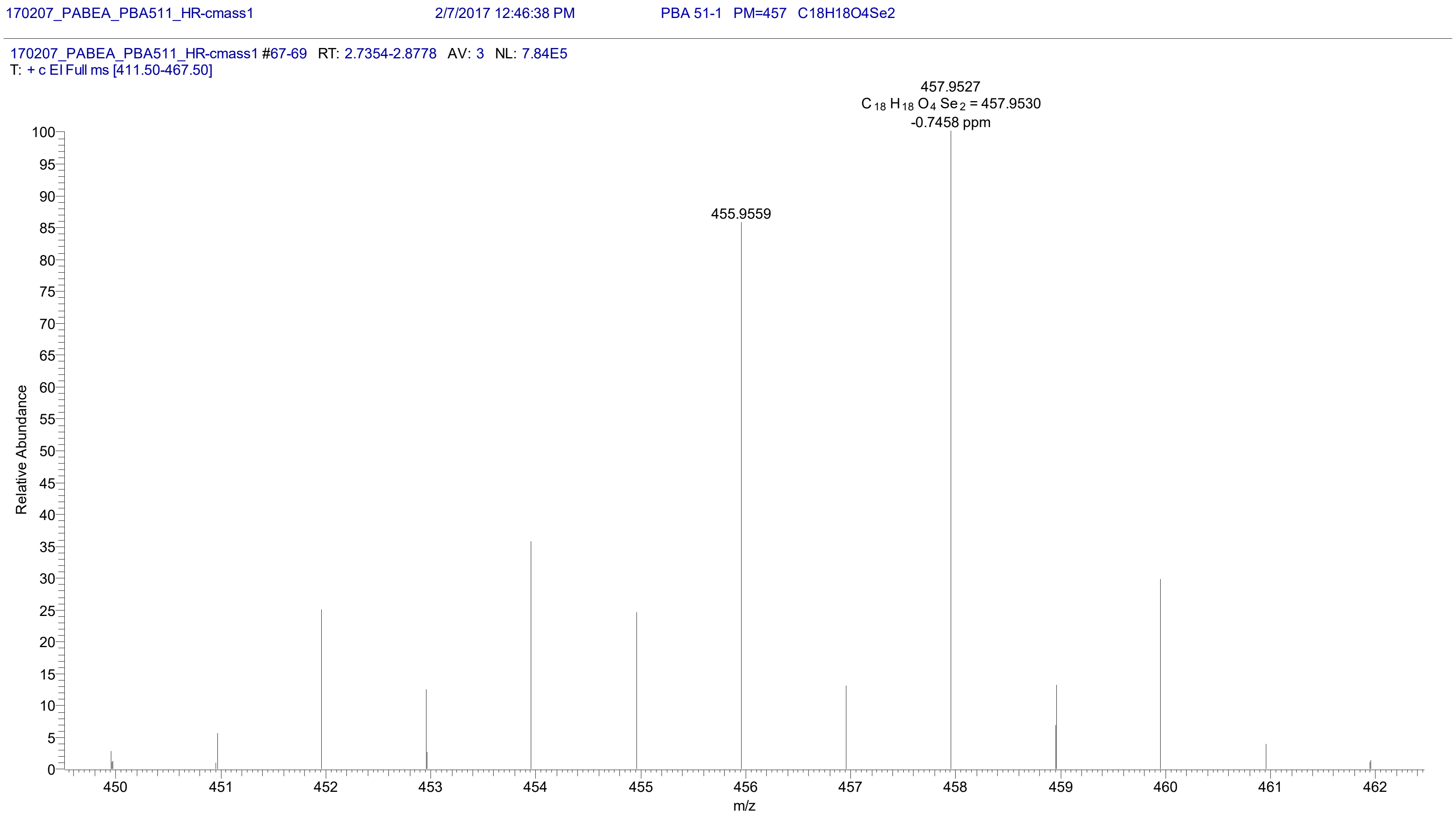

HRESI-MS spectrum of **40**

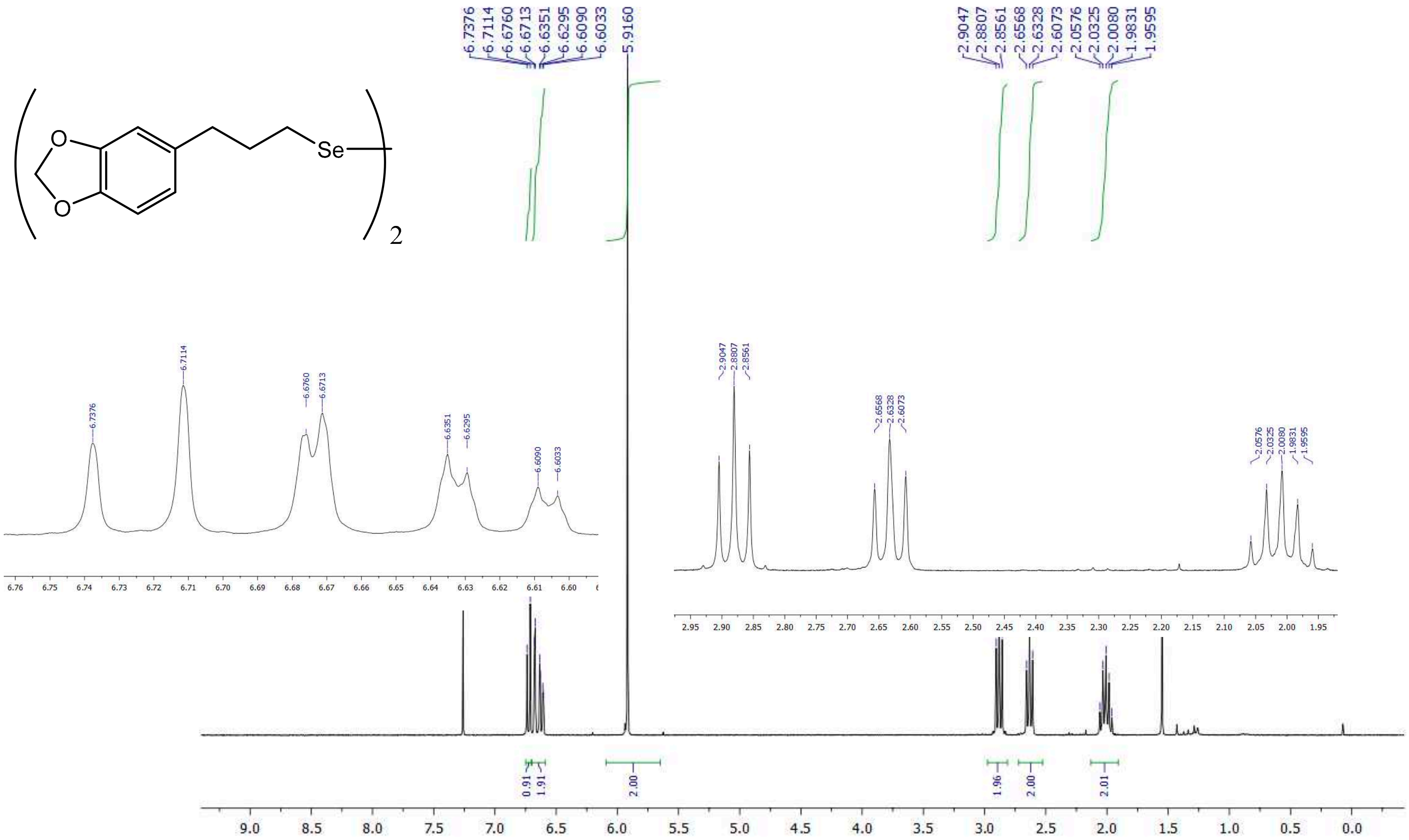

^{1}H- NMR (300 MHz, $CDCl_3$) of **41**

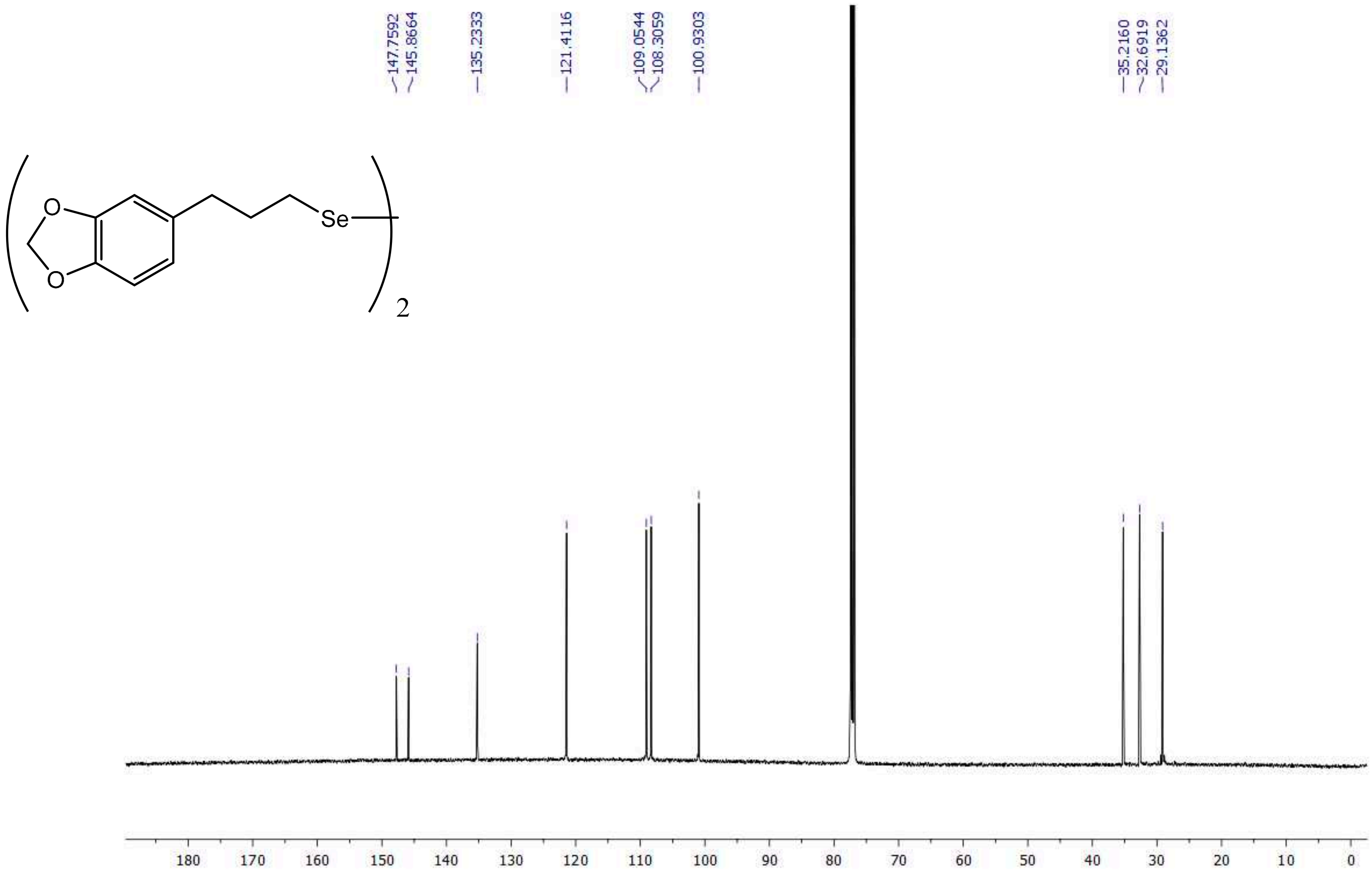

^{13}C- NMR (125.7 MHz, $CDCl_3$) of **41**

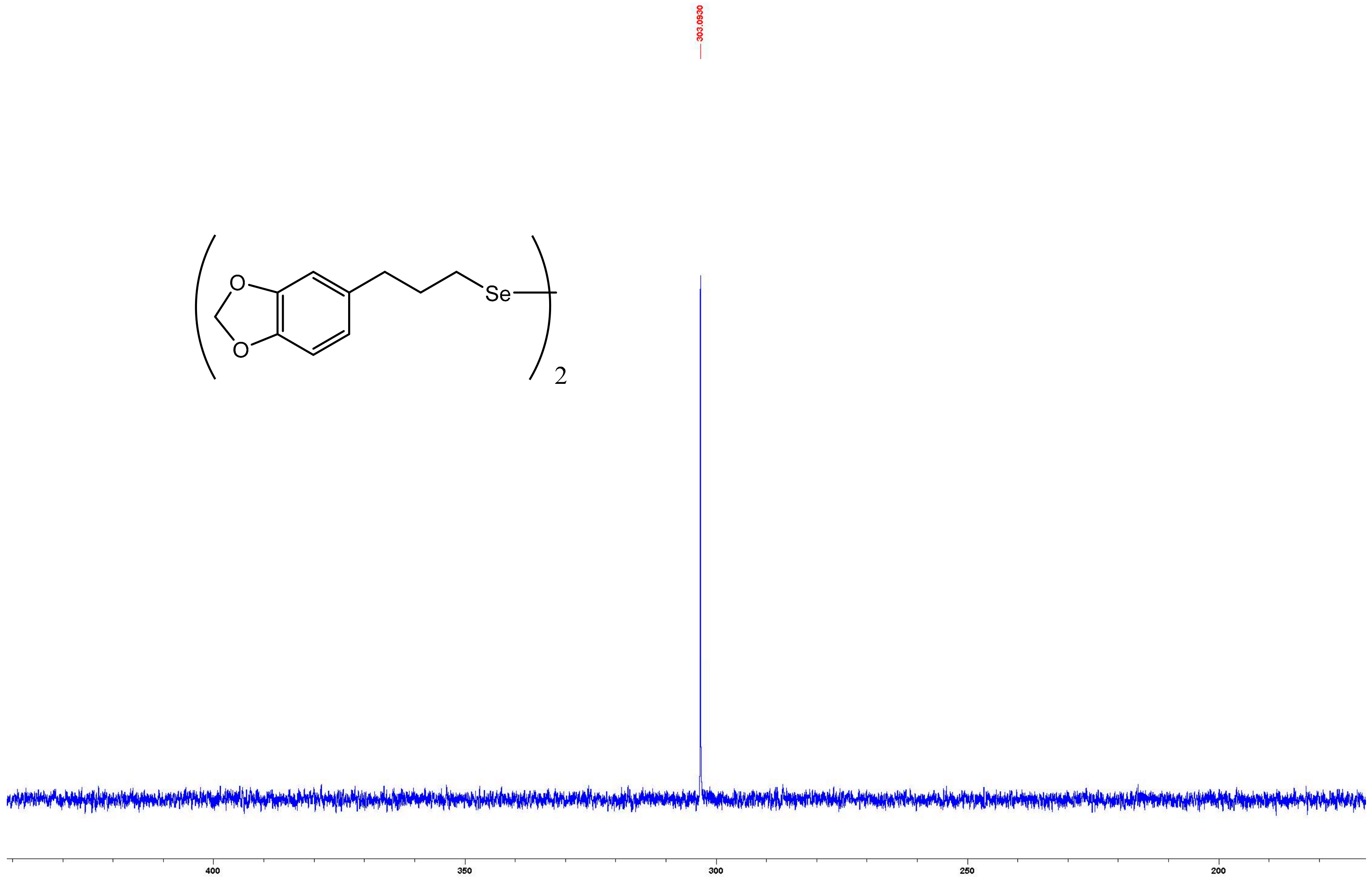

^{77}Se- NMR (95 MHz, $CDCl_3$) of **41**

160624_RPP6_ACN 06/24/16 17:02:27 RPP-6 PM=485 C20H22O4Se2

160624_RPP6_ACN #84-107 RT: 0.34-0.44 AV: 24 SB: 140 1.28-1.85 NL: 2.67E5
T: FTMS + c ESI Full ms [60.00-900.00]

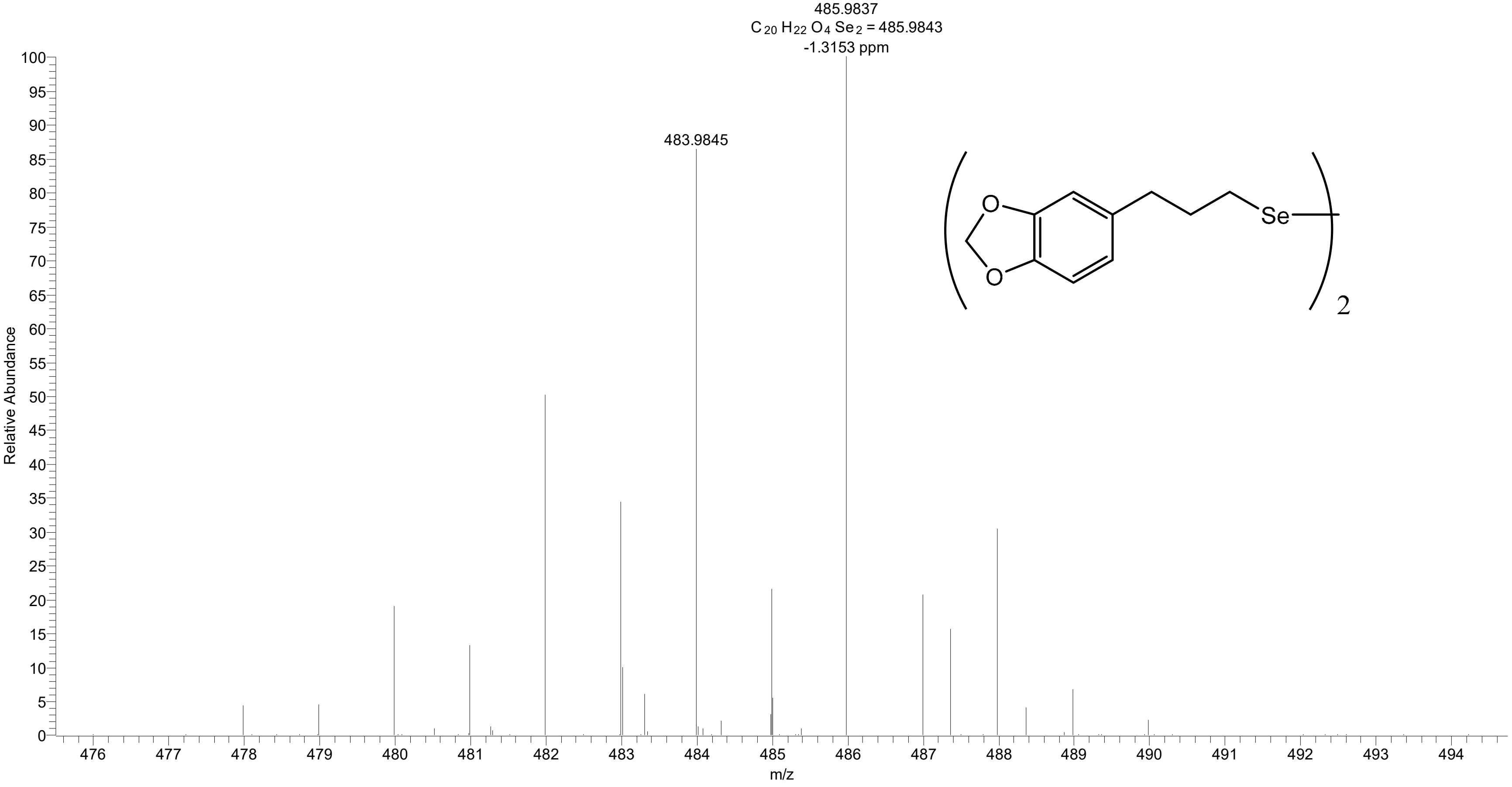

HRESI-MS spectrum of **41**

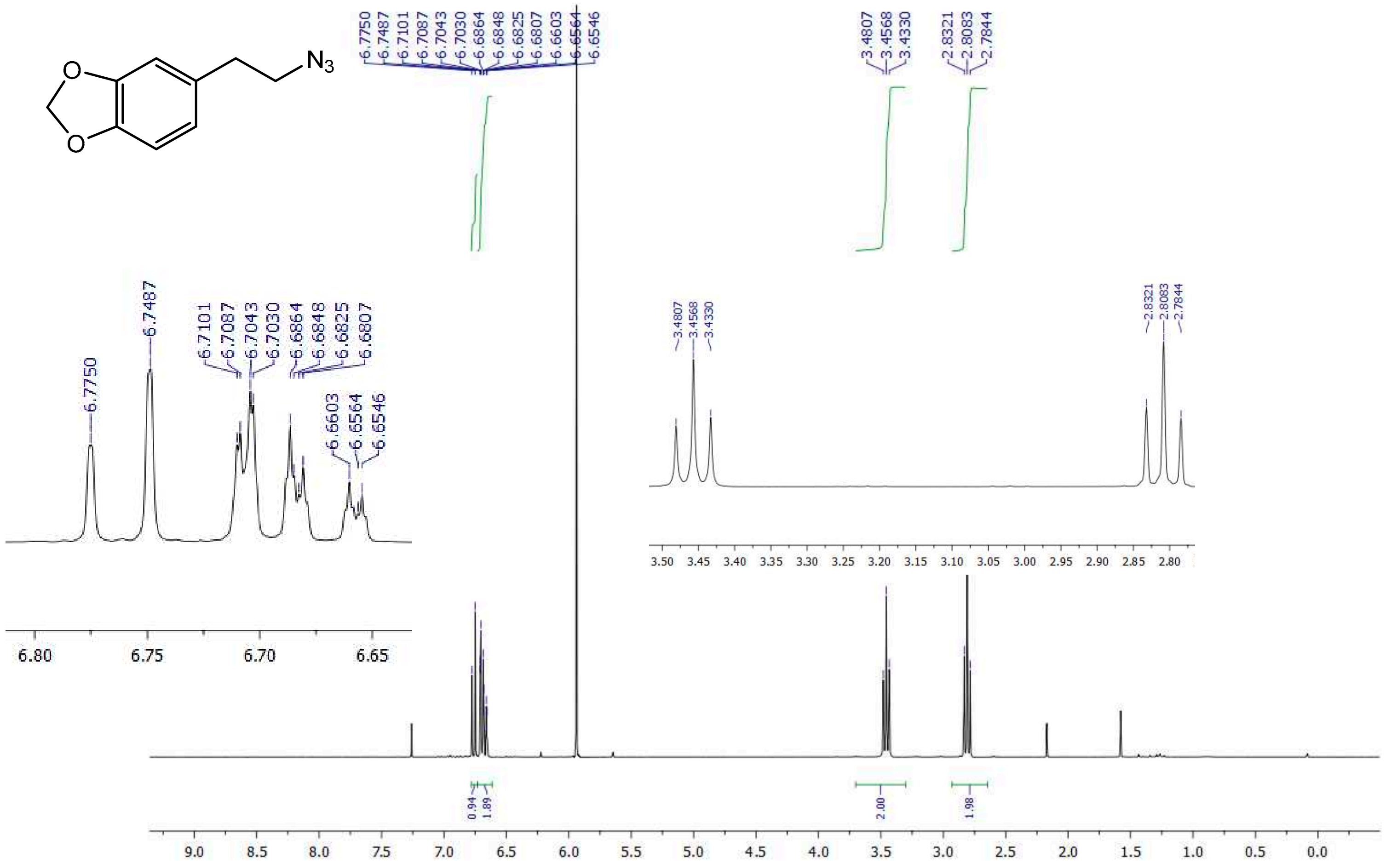

^{1}H- NMR (300 MHz, $CDCl_3$) of **42**

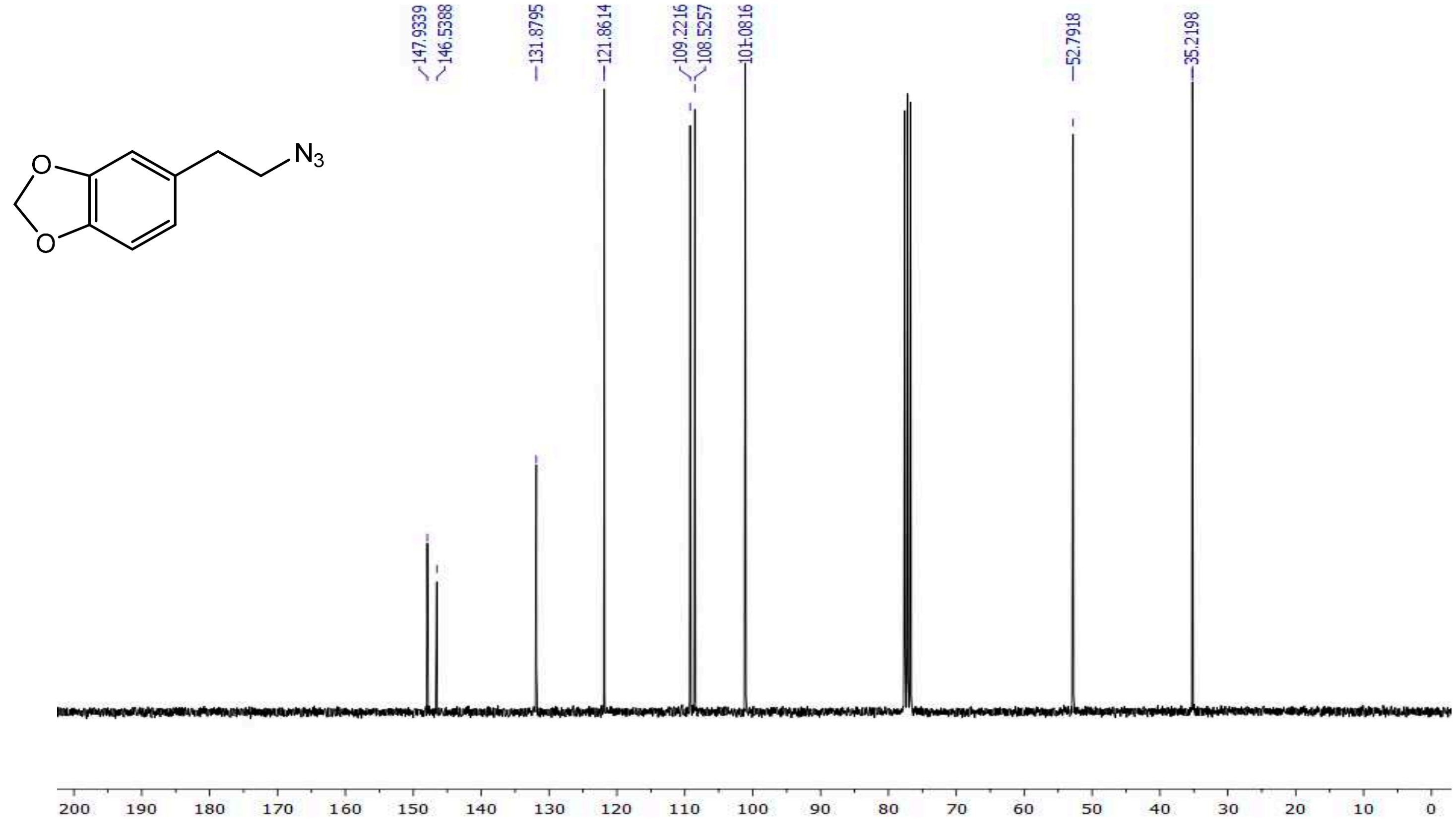

^{13}C- NMR (75.5 MHz, $CDCl_3$) of **42**

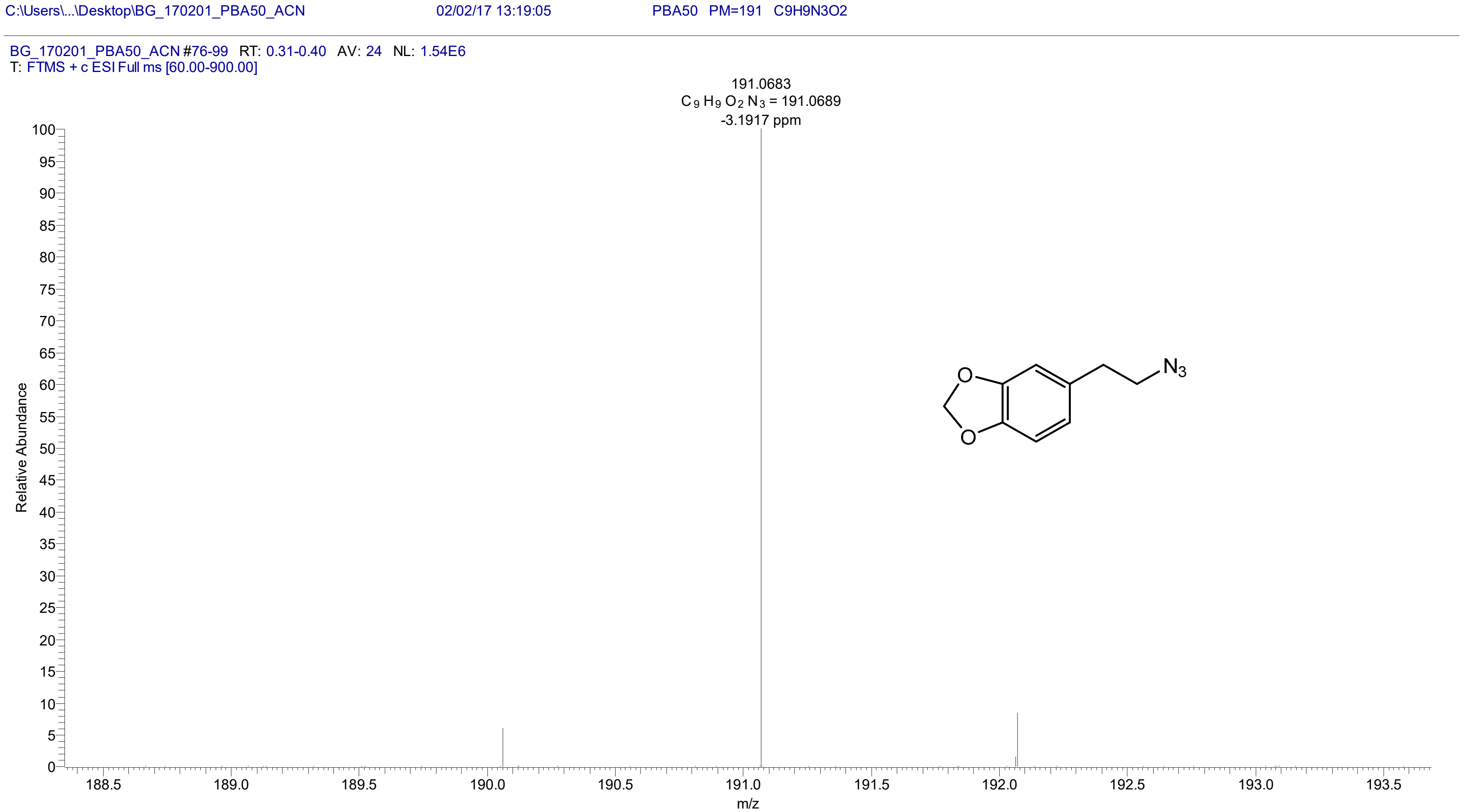

HRESI-MS spectrum of **42**

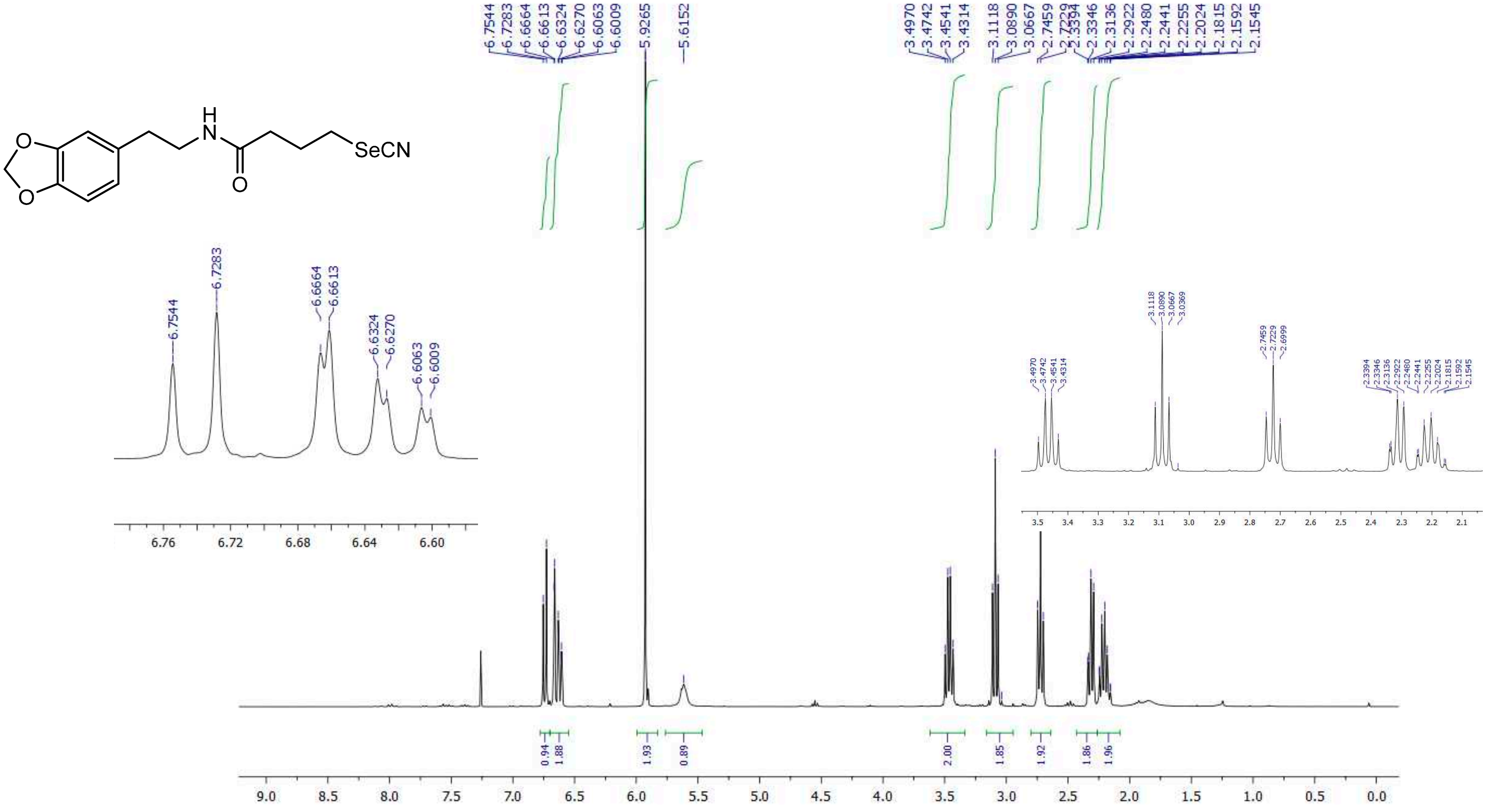

^{1}H- NMR (300 MHz, $CDCl_3$) of **45**

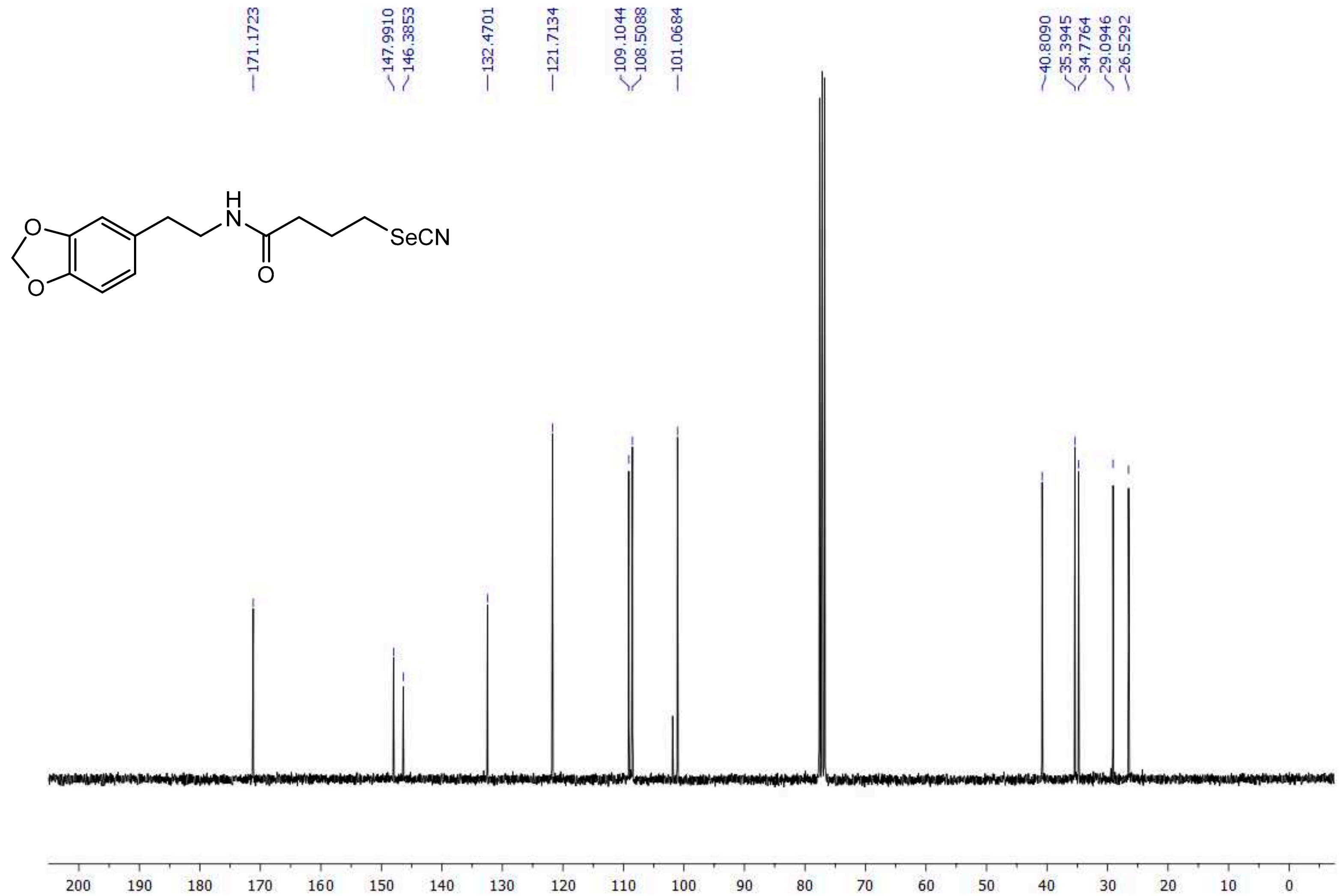

^{13}C- NMR (75.5 MHz, $CDCl_3$) of **45**

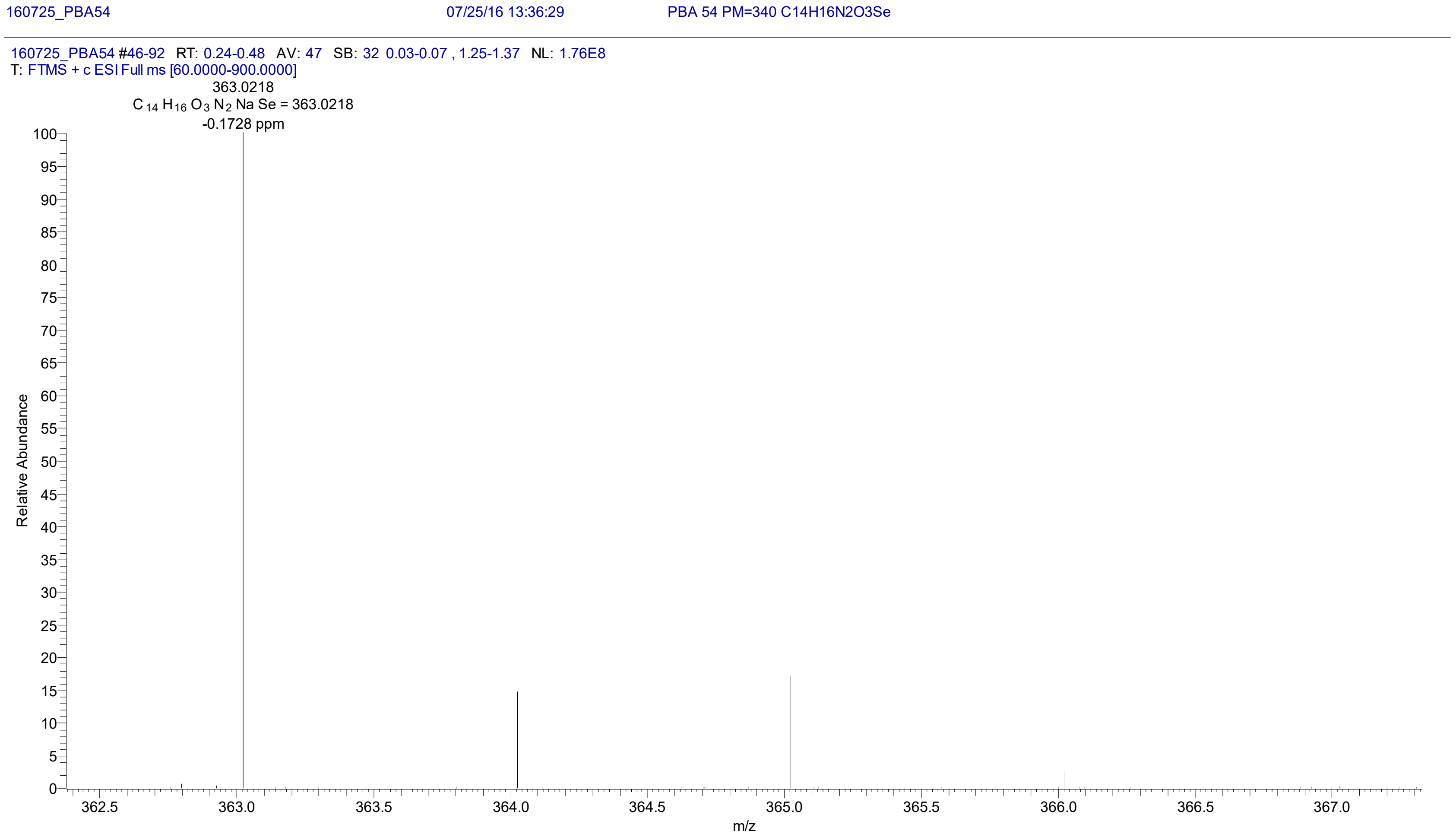

HRESI-MS spectrum of **45**

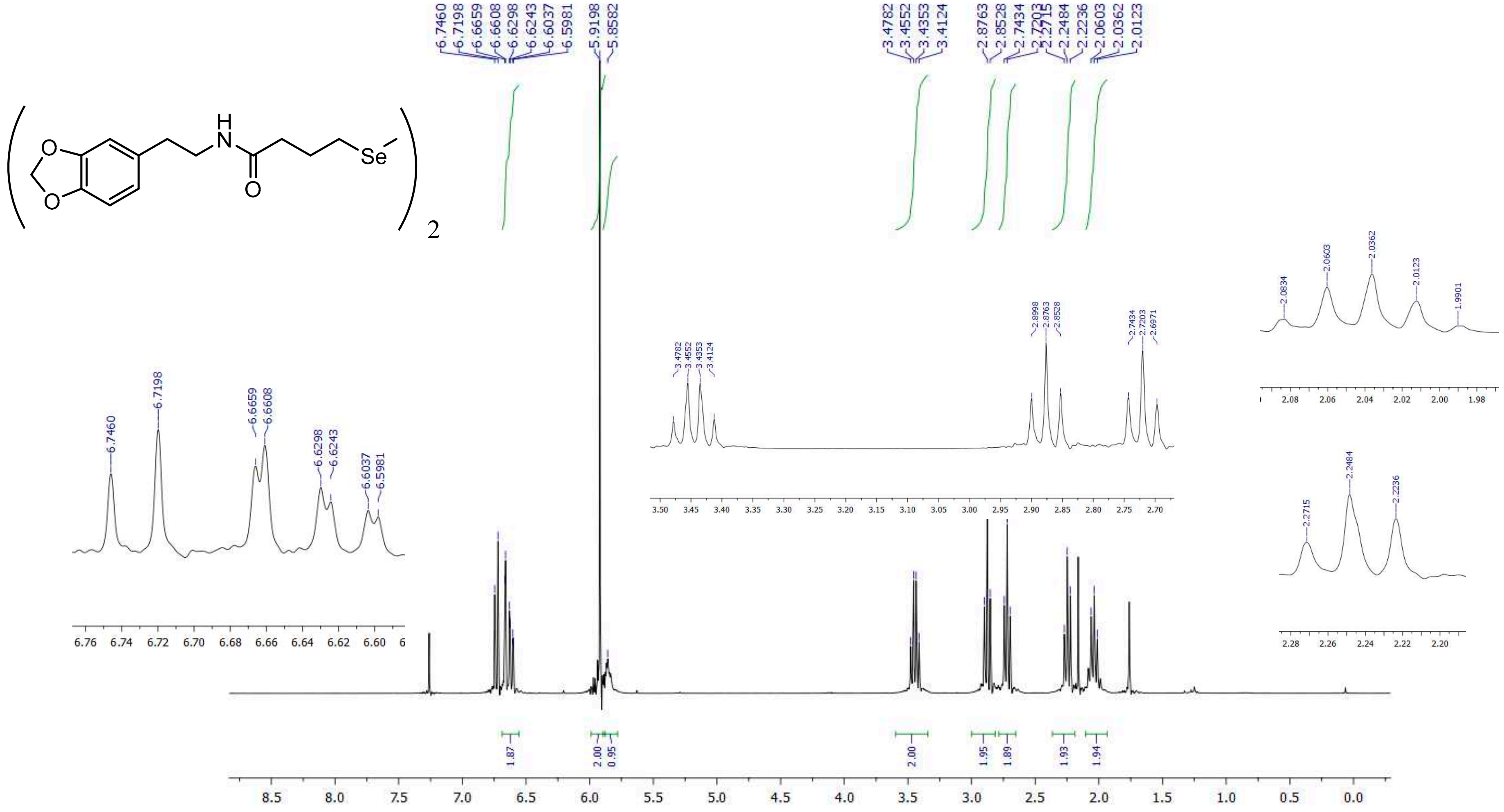

^{1}H- NMR (300 MHz, $CDCl_3$) of **46**

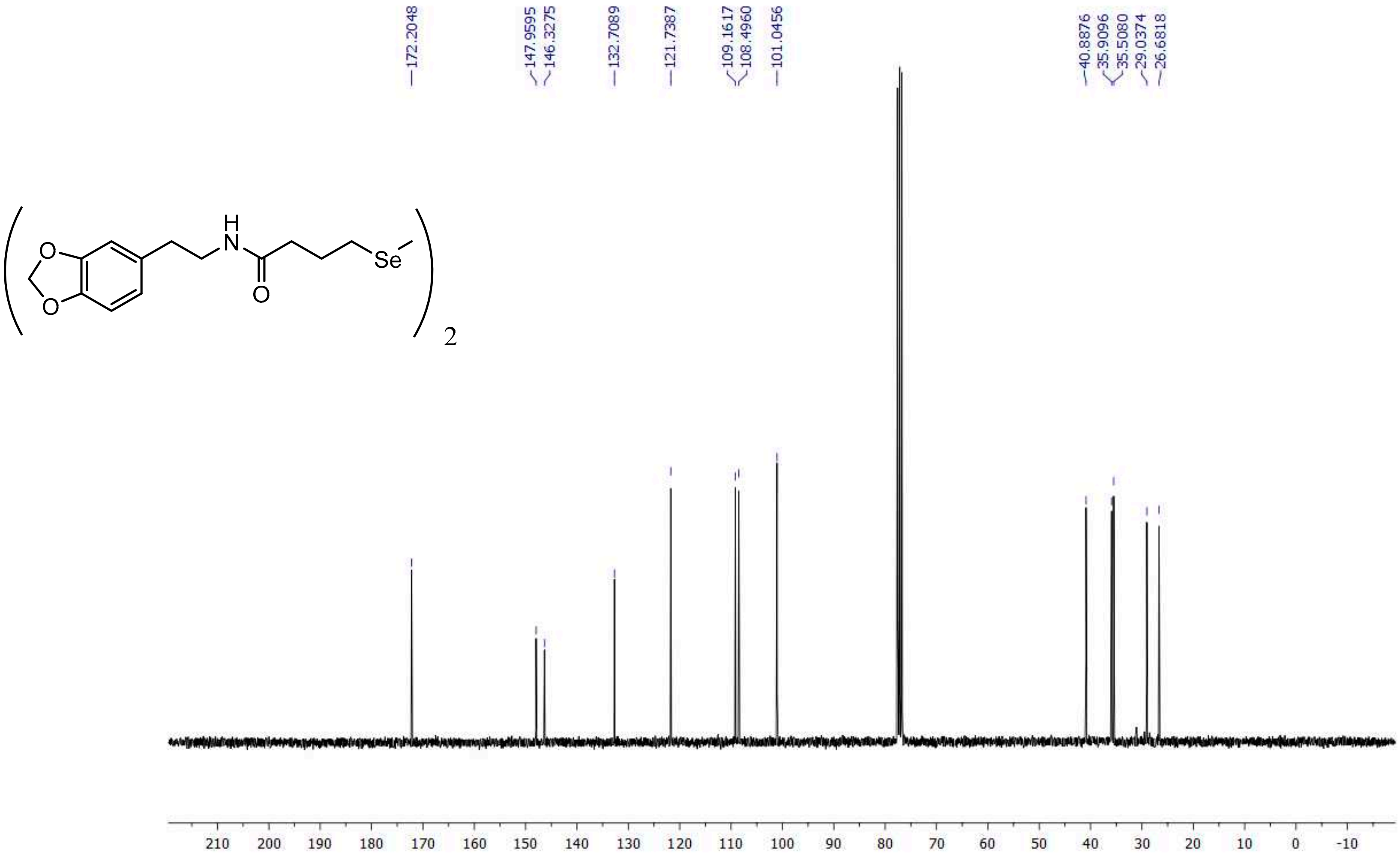

^{13}C- NMR (75.5 MHz, $CDCl_3$) of **46**

160727_PBA541_ACN 07/27/16 12:40:40 PBA 54-1 PM=600 C26H36N2O4Se2

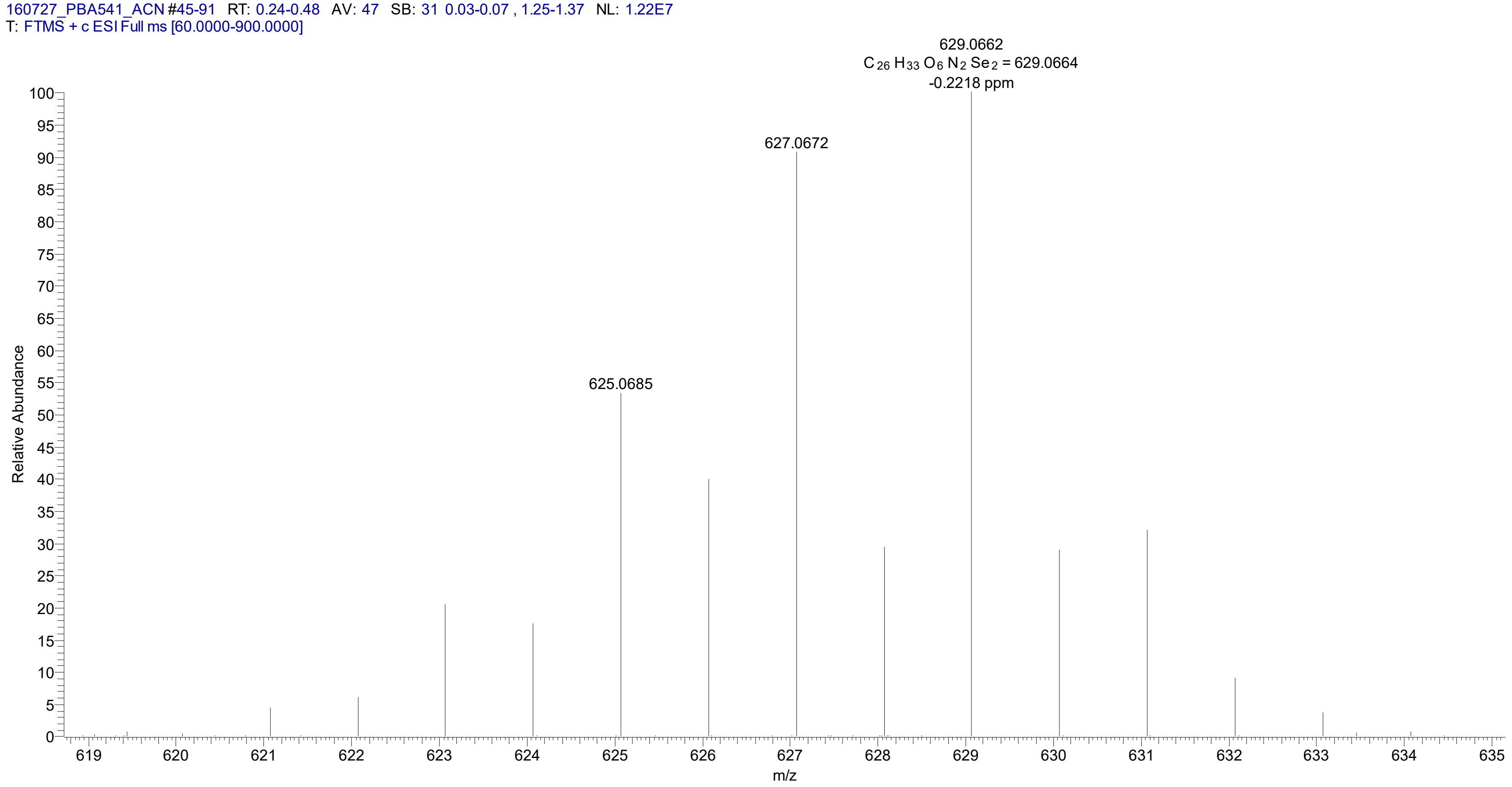

HRESI-MS spectrum of **46**
